# Single-cell transcriptomics reveals differences between chorionic and basal plate cytotrophoblasts and 2D-cultured trophoblast stem cells

**DOI:** 10.1038/s42003-026-10460-0

**Published:** 2026-06-10

**Authors:** Robert Morey, Francesca Soncin, Sampada Kallol, Nirvay Sah, Zoe Manalo, Tony Anh Bui, Jaroslav Slamecka, Virginia Chu Cheung, Donald Pizzo, Daniela F. Requena, Ching-Wen Chang, Omar Farah, Ryan Kittle, Kelly Lam, Morgan Meads, Mariko Horii, Kathleen M. Fisch, Mana M. Parast

**Affiliations:** 1 Department of Pathology, School of Medicine, University of California San Diego, La Jolla, CA USA; 2 Sanford Consortium for Regenerative Medicine, La Jolla, CA USA; 3 Center for Perinatal Discovery, University of California San Diego, La Jolla, CA USA; 4 Department of Obstetrics, Gynecology, and Reproductive Sciences, School of Medicine, University of California San Diego, La Jolla, CA USA

**Keywords:** Developmental biology, Multipotent stem cells, Stem cells, Multipotent stem cells

## Abstract

Cytotrophoblast (CTB) of the early gestation human placenta are bipotent progenitor epithelial cells, which can differentiate into invasive extravillous trophoblast (EVT) and multinucleated syncytiotrophoblast (STB). Trophoblast stem cells (TSC), derived from early first trimester placentas, have also been shown to be bipotential; however, their cell-of-origin has not been identified. In this study, we set out to probe the transcriptional diversity of early and late first trimester villous CTB (vCTB) and compare these to TSC. To this end, we performed single-cell RNA sequencing (scRNA-seq) on placental villous tissue from early (6-8 weeks) and late (12-14 weeks) first trimester placentas; we also evaluated CTB within basal (maternal) and chorionic (fetal) regions of early first trimester placenta, both by scRNA-seq and GeoMx-based spatial transcriptomics. Finally, we performed scRNA-seq on three TSC lines, derived from 6-8 week gestation placentas, as well as on early first trimester CTB at several timepoints during TSC derivation. We observed differences within CTB clusters associated with gestational age, further influenced by location near the basal or chorionic plates. We identified trophoblast states representing “initial state” vCTB (in vivo CTB progenitors), as well as additional CTB subtypes, precursor STB, and precursor and mature EVT. CTB progenitors appeared enriched in early first trimester placentas at the basal plate; additionally, basal plate CTB showed transcriptional features consistent with EVT bias, whereas chorionic plate CTB showed features associated with STB precursors. Clustering and trajectory inference analysis indicated that TSC were most like EVT precursor cells. In fact, vCTB lost their in vivo “initial state” markers, including PAGE4, as they transitioned to TSC during in vitro culture. This was confirmed by flow cytometric analysis of 6 different TSC lines, which showed uniform expression of proximal column markers ITGA2 and ITGA5. Additionally, we found that ITGA5^+^ CTB could be plated in 2D, readily differentiating only into EVT in the absence of lineage-directed cues, but failed to form self-renewing organoids; conversely, ITGA5^-^ CTB could not be plated in 2D, but readily formed organoids. Our findings suggest that distinct CTB states exist in different regions of the placenta as early as six weeks gestation and that current TSC lines most closely resemble ITGA5^+^ proximal column trophoblast, biased toward the EVT lineage.

## Abstract (web summary)

Single-cell transcriptomics reveals distinct cytotrophoblast states in the first trimester placenta and shows that 2D trophoblast stem cells resemble EVT-biased proximal column cells rather than bipotential villous progenitors.

## Introduction

The development of the human placenta has been a black box, particularly early in gestation, when the organ is difficult to probe during an ongoing pregnancy^1–3^. Following implantation, the cytotrophoblast (CTB) shell expands, and invaginations soon give rise to primary and secondary chorionic villi^4,5^. Villous CTB (vCTB) progenitor cells differentiate into two main mature trophoblast types: syncytiotrophoblast (STB), which arise by CTB fusion and serve at the nutrient/gas exchange interface of the floating chorionic villi; and extravillous trophoblast (EVT), which arise through epithelial-mesenchymal transition within the anchoring villi, ultimately invading through decidua and the upper third of the uterine myometrium, remodeling maternal spiral arterioles in order to access oxygenated blood for continuous growth and development of the fetus^6^. In 2018, trophoblast stem cells (TSC) were derived from early gestation vCTB, and media formulations developed to allow these cells to either self-renew or differentiate into both EVT and STB^7^. This work significantly advanced our ability to study this early “black box” period of placental development, spawning a wealth of publications on factors and pathways that regulate human trophoblast differentiation^8–13^. However, while these cells are derived from ITGA6^+^ vCTB, it is unclear how well they represent this bipotential cell population, particularly given their lack of undirected differentiation into STB.

Recently, Sheridan et al.^14^ compared TSCs to trophoblast organoids (T-Org), which can also be derived from early gestation placentas, and are composed of an outer layer of proliferative CTB and an inner layer of spontaneously-formed STB^15,16^. They found that TSCs have a distinct transcriptome, expressing several markers of cells in the proximal column of anchoring villi, regions which harbor cells transitioning into EVT^14^. Subsequently, Shannon et al. applied single cell RNA-sequencing (scRNA-seq) to compare T-Org and TSC grown in 3D as organoids (TSC-Org) to primary trophoblast within the early gestation placenta and found that TSC-Org show features of both CTB and EVT^17,18^. Here, we set out to evaluate CTB heterogeneity in first trimester human placenta at the single cell level, first comparing early (6–8 weeks) to late (12–14 weeks) first trimester CTB, and subsequently, identify a bipotential “initial state” CTB, which we find to be enriched within the basal plate (BP, maternal surface), rather than the chorionic plate (CP, fetal surface) of early first trimester placentas. We then use a combination of spatial transcriptomics and scRNA-seq to identify important differences in differentiation potential between CTB in the BP vs. CP regions of first trimester placenta. We then superimpose single cell transcriptomics from TSC, cultured in 2D, onto our in vivo dataset, and determine that these cells are distinct from the “initial state” villous CTB (vCTB), and most closely resemble, not vCTB, but proximal column trophoblast, on their way to differentiating into EVTs. Using scRNA-seq and flow cytometry, we note the loss of initial state CTB and gain of EVT progenitor state features during the TSC derivation process. Finally, we confirm by flow cytometry that TSCs uniformly express proximal column trophoblast/EVT progenitor markers, ITGA5 and ITGA2, and show that similar cells isolated from first trimester placentas exclusively differentiate into EVT. We conclude that TSCs, cultured in 2D, represent a unique cell type, resembling only rare cells in vivo, and are distinct from bipotential villous CTB in early gestation placentas.

## Results

### Transcriptional topography of first trimester trophoblast at single cell resolution revealed heterogeneity of cytotrophoblast based on gestational age

**Fig. 1:**
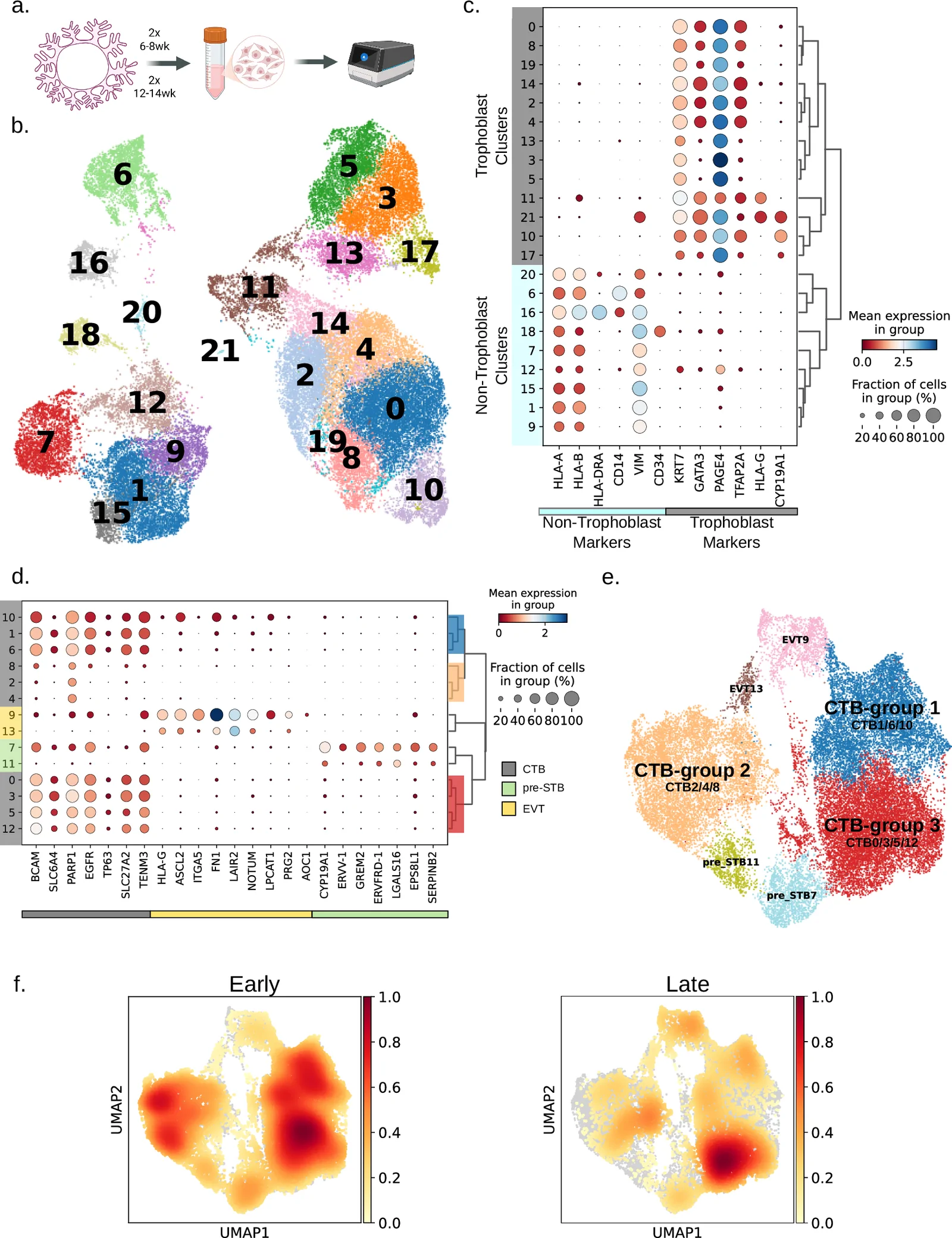
Integrated placental single cell RNA-seq data, showing distinct CTB groups in early vs. late first trimester. **a** Schematic showing experimental setup (scRNA-seq of four whole placentas, 2 early and 2 late first trimester, following dissociation). Created in BioRender. Soncin, F. (2026) https://BioRender.com/a1v76tu. **b** UMAP of all placental cells following quality control filtering, integration, and clustering. **c** Dot plot and hierarchical clustering of cell clusters using trophoblast and non-trophoblast specific gene expression. **d** Dot plot and hierarchical clustering of cell clusters using cell type specific gene expression. Dendrogram is colored based on the CTB group color in the UMAP in part (**e**). **e** UMAP of trophoblast cells annotated based on cell type specific gene expression shown in part (**d**). Groups were formed based on hierarchical clustering seen in part (**d**) and in dendrogram in Supplementary Fig. S2d. **f** UMAPs depicting the cell density in early (left) and late (right) first trimester placentas.

To better understand the transcriptional landscape of first trimester human trophoblast, we performed single cell RNA-seq on four first trimester normal placentas, two early (6–8 weeks gestational age/GA) and two late (12–14 weeks GA). Only the gestational sac and associated villous tissue (no decidual tissues) were dissociated into single cells. In total, ~60,000 cells were captured using the 10X Genomics platform and sequenced (Fig. 1a, Supplementary Data 1). The samples underwent ambient RNA removal, doublet identification and filtering, as well as cell and gene quality control and filtering (See Methods), resulting in ~44,000 cells that were then clustered, integrated, and visualized using uniform manifold approximation and projection (UMAP)^19–22^ (Supplementary Fig. S1a, b). Leiden clustering resulted in 22 clusters, and based on marker genes, the non-trophoblast cells generally clustered on the left, and the trophoblast cells generally clustered on the right side of the UMAP (Fig. 1b). Clusters were annotated as either trophoblast or non-trophoblast in origin using the expression of trophoblast specific genes (such as *KRT7, GATA3, PAGE4, TFAP2A, HLA-G, CYP19A1*) and non-trophoblast genes (such as *HLA-A, HLA-B, HLA-DRA, CD14, VIM, CD34*) (Fig. 1c, Supplementary Data 2). We were interested exclusively in the trophoblast clusters (0, 2, 3, 4, 5, 8, 10, 11, 13, 14, 17, 19) and removed all non-trophoblast clusters (Supplementary Fig. S2a). After the removal of the non-trophoblast clusters, we reclustered the remaining trophoblast cells into 14 clusters and recalculated the marker genes for each cluster (Supplementary Fig. S2b, c, Supplementary Data 3).

Next, we annotated the clusters using a panel of curated marker genes, into three broad trophoblast cell types: 1) cytotrophoblast (CTB) (*BCAM*, *SLC6A4*, *PARP1*, *EGFR*, *TP63*, *SLC27A2*, *TENM3*); 2) extravillous trophoblast (EVT) (*HLA-G*, *ASCL2*, *ITGA5*, *FN1*, *LAIR2*, *NOTUM*, *PRG2*, *AOC1*); and 3) pre-syncytiotrophoblast (pre-STB) (*CYP19A1*, *ERVV-1*, *GREM2*, *ERVFRD-1*, *LGALS16*, *EPS8L1*, *SERPINB2*)^23^. We designated cells expressing canonical syncytiotrophoblast (STB) specific genes as pre-STB because STB are large multinucleated cells that presumably would not be able to be captured by the 10X Genomics Chromium platform. The resulting 14 clusters included ten different CTB clusters, two EVT clusters, and two pre-STB clusters (Fig. 1d, Supplementary Fig. S2b, Supplementary Data 3, S4). Based on hierarchical clustering, the CTB clusters could be combined into three groups (Fig. 1e, Supplementary Fig. S2d): CTB-group 1 (containing clusters CTB1, CTB6, and CTB10) and CTB-group 2 (containing CTB2, CTB4, and CTB8) were transcriptionally similar, and distinct from CTB-group 3 (containing clusters CTB0, CTB3, CTB5, and CTB12) (Fig. 1e, Supplementary Fig. S2d, Supplementary Data 5). To determine how our CTB groups align with previously reported trophoblast subtypes, we used the top 200 differentially expressed genes from each CTB group to score the dataset from Arutyunyan et al.^8^. All three groups corresponded to cells annotated as subtypes of villous cytotrophoblast (Supplementary Fig. S2e). We noted that our CTB-group 2 cells had lower expression of our designated CTB markers such as *EGFR* compared to groups 1 and 3; however, they showed high expression of *VGLL1*, a marker that, within the placenta, is specific to trophoblast and most highly expressed in villous CTB, as previously shown by Vento-Tormo et al. (Supplementary Fig. S2f)^24^.

To better understand the differences between the cells comprising each CTB group, we determined the cell density of the early and late first trimester cells (Fig. 1f, Supplementary Data 4) and noted that, while all CTB groups contained early first trimester cells, CTB-group 1 and CTB-group 2 contained only a small number of late first trimester cells. We found that our late first trimester-enriched CTB-group 3 expressed 10 genes significantly higher than groups1 and 2. These enriched genes (*OLR1, VSIR, SLC40A1, SLC22A11, FLT1, CSF3R, KDM5B, FBN1, MALAT1, CCDC18-AS1*) play regulatory roles in the placenta, particularly in angiogenesis, immune regulation, structural integrity, and cellular differentiation, all of which are vital for successful pregnancy and fetal health (Supplementary Data 6). To independently validate early and late first trimester CTB differences, we reanalyzed published scRNA-seq data from early (mean gestational age 6.4 weeks) and late (mean gestational age 11.3 weeks) first-trimester placental samples^25^. The seven samples were integrated, clustered, and annotated using the same analytical workflow applied to our dataset (Supplementary Fig. S3a–d). Differential expression analysis was then performed between CTB clusters predominantly composed of early versus late first-trimester cells (Supplementary Fig. S3e). Consistent with our findings, all genes identified in our late first trimester–enriched CTB-group 3, with the exception of *KDM5B* and the lncRNA *CCDC18-AS1*, showed similar expression patterns in this independent dataset (Supplementary Data 6).

Next, we determined the differences between the CTB groups that contained relatively few late first trimester cells (CTB groups1 and 2). Using genes enriched in CTB-group 1 by at least a log2 fold change of 1.5, we found that the top five MSigDB Hallmark pathways significantly (adjusted *p*-value < 0.05) enriched were pathways related to cell proliferation and differentiation (G2-M checkpoint, E2F targets, Myc targets v1, Epithelial Mesenchymal transition/EMT, and Apical junction) (Supplementary Data 7). Genes enriched in CTB-group 2 were significantly enriched for pathways relating to KRAS signaling and immune response (Allograft rejection, Complement, and Inflammatory Response) (Supplementary Data 8). Notably, although CTB-group 2 did not express any EVT or pre-STB markers and clustered with the other CTB clusters, only a small fraction of cells expressed mature cytotrophoblast markers such as *EGFR, TP63*, and *BCAM*, compared to the other CTB groups (Fig. 1d).

### Trophoblast trajectory inference identifies a common progenitor CTB cluster

**Fig. 2:**
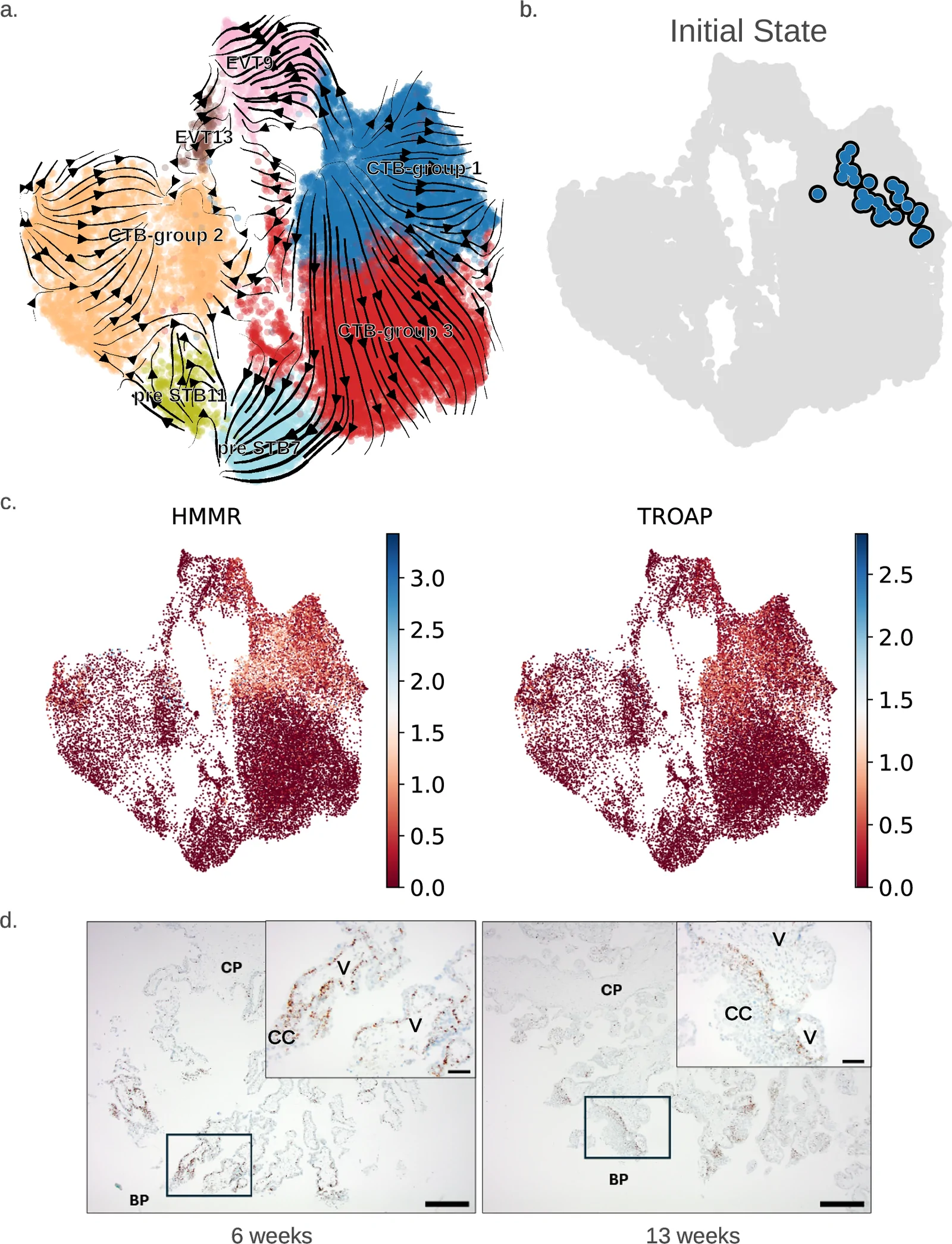
Identification of “initial state” CTB within early first trimester placentas. **a** RNA velocity projected as streamlines on the integrated first trimester trophoblast UMAP. CTB names refer to CTB groups after combining CTB clusters in Fig. 1d. **b** Initial state cells (*n* = 30) as calculated by CellRank. **c** Expression of the initial state markers *HMMR* and *TROAP* on the integrated first trimester trophoblast UMAP. **d** Representative in-situ hybridization of *HMMR* expression in early and late first trimester placenta (*n* = 5 per group). Low-power micrographs show chorionic (CP) and basal (BP) plate regions (scale bar = 625 μm); inset is a magnified micrograph of the BP region (scale bar = 125 μm), with villous stroma (v) and trophoblast cell column (cc) further annotated. Note that overall signal (brown) is higher at 6-week gestation and at/near the basal plate.

Next, to study the cellular dynamics and relationships between cells and clusters, we performed trajectory inference on our integrated trophoblast cells using RNA velocity^26^. In agreement with previous results, our analysis suggested a common CTB initial state (CTB-group 1) with two differentiation trajectories; one to a terminal EVT (EVT9) cell population and one to a pre-STB (pre-STB7) cluster (Fig. 2a). Through ranking genes based on their cluster-specific differential velocity expression (significantly higher or lower compared to the remaining clusters in the population), we identified genes that best explained the RNA velocity vector field (Supplementary Data 9). We found that the rank velocity genes (r^2^ > 0.5 & ρ > 0.3) in CTB-group 1 were significantly enriched in the epithelial mesenchymal transition (EMT) hallmark pathway (adj. *p*-value < 0.1 × 10^−8^) as well as the Reactome regulation of IGF binding protein pathway (adj. *p*-value < 0.0005). Additionally, we were interested in marker genes that were differentially expressed in the initial state, which was calculated using CellRank^27^^,28^ and included only early first trimester cells (Fig. 2b). We therefore looked for genes that were differentially enriched (adj. *p*-value < 0.05 & log fold change > 1) in the initial state cells (*n* = 30) compared to the rest of the trophoblast cells in our integrated trophoblast dataset and were part of the CTB1 rank velocity genes (Supplementary Data 9). These differentially-enriched initial state genes included the cell division- and cell motility-linked gene *HMMR*, the mitotic spindle- and implantation-linked gene *TROAP* (Fig. 2c), and several transcriptional regulators such as *MXD3, CENPA, ZNF302, ZNF165, ZNF701, TET1, KMT2A, KLF6*, and *TEAD1* (Supplementary Fig. S4a, b, Supplementary Data 10). We scored Arutyunyan et al.’s^8^ snRNA-seq data with our differentially-enriched (adj. *p*-value < 0.05 & log fold change > 2) initial state genes and noted that these are most enriched in their proliferative villous CTB cluster (Supplementary Fig. S4c). To identify the location of these initial state cells within the placenta, we performed in-situ hybridization for *HMMR* and *TROAP*, in five early (6 week) and five late (13 week) first trimester placental tissues. We confirmed that these cells appeared to be more numerous in early first trimester placentas at the basal, compared to chorionic plate (Fig. 2d).

### Spatial transcriptome analysis reveals differences between basal and chorionic plate CTB

The chorionic (CP) and basal (BP) plate constitute the fetal and maternal surfaces of the placenta, respectively, with the chorionic villi emanating from the former and being anchored within the latter. These two surfaces are functionally different, but the CTB composition of these two regions has yet to be systematically evaluated. As expected, our initial state cells were predominantly from early first trimester placentas (Figs. 1f and 2b, c), and more abundant at the basal plate (Fig. 2d). To better understand the composition of the cell types that made up our early first trimester CTB, we used the GeoMx digital spatial profiler, and applied its whole transcriptome atlas (WTA) panel to 3 additional 6-week placentas (Supplementary Data 1), selecting regions of interest (ROI’s) at/near the CP (sac) or BP (anchoring villi) of these placentas, and segmenting on CTB by EGFR immunostaining (Supplementary Fig. S5a, b). We next performed differential expression analysis (adj. *p*-value < 0.05 & log fold change >1) between the EGFR^+^ CTB in BP vs. CP ROIs (Supplementary Fig. S5c, Supplementary Data 11). We then asked what cells in our integrated early and late first trimester placental scRNA-seq dataset had gene expression profiles that were most similar to our BP vs. CP CTB cells. To do this, we scored our scRNA-seq cells using the significantly enriched BP and CP genes from our spatial dataset. We found that the BP-CTB-enriched genes were more highly expressed in the EVT clusters, and the CP-CTB-enriched genes were more highly expressed in our pre-STB clusters (Wilcoxon rank-sum *p*-value < 0.001) (Supplementary Fig. S5d). These data suggest that there may be important differences in differentiation potential between EGFR^+^ CTB in the BP and CP regions of first trimester placenta.

**Fig. 3:**
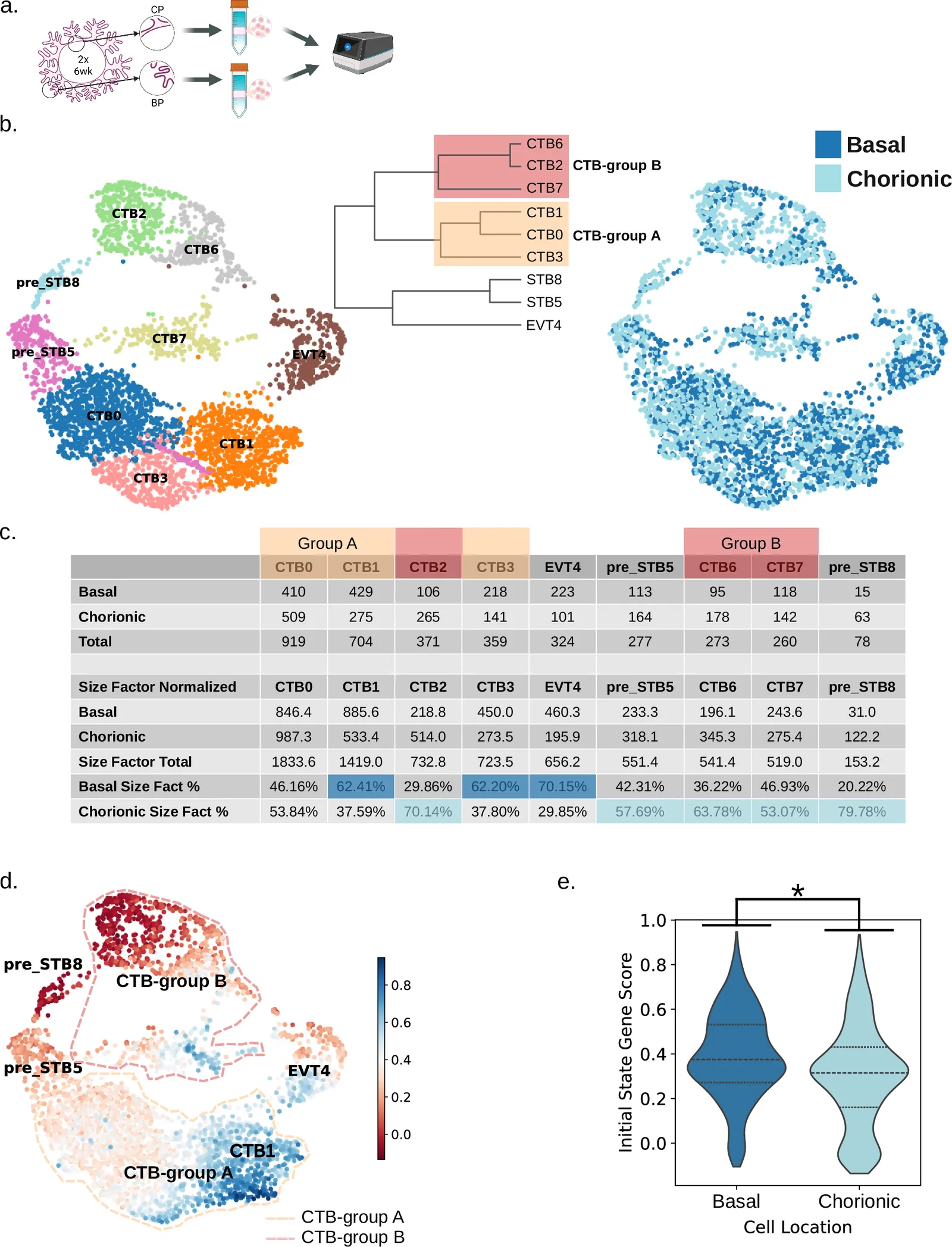
Identification of distinct CTB groups within basal and chorionic plate regions of early first trimester placenta. **a** Schematic showing separation of chorionic plate (CP) and basal plate (BP) regions of 2 early first trimester placentas, followed by CTB isolation using Percoll gradient centrifugation, and scRNA-seq. Created in BioRender. Soncin, F. (2026) https://BioRender.com/a1v76tu. **b** UMAP of annotated and integrated basal and chorionic plate trophoblast from two early first trimester placentas, showing individual clusters (left) and placental region (dark blue for basal plate and light blue for chorionic plate) (right). Dendrogram (middle) shows transcriptomic similarity (Pearson correlation) between UMAP clusters. **c** Table showing the number of cells comprising each cluster, including both raw numbers and size factor-normalized, originating from either the basal or chorionic plate. **d** Expression of the initial state markers (Supplementary Data 10), in the basal and chorionic plate UMAP. Dashed lines mark approximate CTB groups, as annotated in part (**b** and **c**). **e** Violin plot showing the initial state score difference between all the basal and chorionic plate cells (Wilcoxon rank-sum test, *p*-value = 1.67e−33 indicated by *). Dashed lines show quartiles.

To better understand the differences between cells in the basal and chorionic plate we dissected the CP (sac) and BP (anchoring villous tips) regions of two additional early first trimester placentas, isolated CTB from each of these regions using Percoll gradient centrifugation, and performed single cell RNA-seq on the resulting four samples (Fig. 3a, Supplementary Data 1). The capturing of the two compartments was confirmed by enrichment of *CDX2* and *ASCL2* in the CP and BP cells, respectively, by qPCR^29–31^ (Supplementary Fig. S6a). Following integration and removal of non-trophoblast cells (Supplementary Fig. S6b), the data were re-clustered (Fig. 3b), broadly annotated using the same marker genes as with the early and late first trimester whole placenta data (Supplementary Fig. S6c), and the composition source of each cluster was calculated (Fig. 3c). The CTB clusters separated into two groups: CTB-group A was composed of clusters CTB0/1/3, and CTB-group B was composed of clusters CTB2/6/7 (Fig. 3b). Not surprisingly, the EVT cluster contained a higher percentage of cells from the basal plate, and the pre-STB clusters a higher percentage of cells from the chorionic plate (Fig. 3c). Interestingly, after adjusting for the total amounts of basal and chorionic plate cells in the dataset, CTB-group B contained a higher proportion of chorionic plate cells, whereas CTB-group A contained more basal cells (Fig. 3b, c). We next verified that the genes enriched in BP- and CP-CTB in our spatial transcriptomic dataset were similarly expressed in our basal and chorionic plate scRNA-seq dataset. In fact, we observed that the basal and chorionic plate CTB in our scRNA-seq dataset exhibited higher BP- or CP-CTB gene score, respectively, although the difference was more pronounced between the cells with the CP-CTB score (Wilcoxon rank-sum *p*-value < 0.001) (Supplementary Fig. S7a, b).

To identify the location of initial state cells in this BP/CP dataset, we scored the dataset using the initial state genes from our early and late first trimester dataset (Supplementary Data 10). We discovered that the differentially expressed genes representing the initial state exhibited the highest expression levels within the CTB1 cluster, which was primarily (62%) composed of basal plate cells (Fig. 3c, d). Upon comparing all basal and chorionic plate cells using these initial state genes, the basal plate cells exhibited significantly higher expression levels of these genes, compared to the chorionic plate cells (Wilcoxon rank-sum *p*-value < 1.7 ×10^−23^) (Fig. 3e). Additionally, we evaluated expression of our initial state markers, *HMMR* and *TROAP*, as well as the transcriptional regulators *MXD3* and *CENPA*, shown to be enriched in our initial state cells (Supplementary Fig. S4), in the BP/CP dataset and noted that these genes were, again, highly expressed in the CTB1 cluster (Supplementary Fig. S8a, b). We looked more closely at the localization of the initial state genes, *HMMR* and *TROAP*, using in situ hybridization of early first trimester placentas, and confirmed enriched expression of these genes in proximal column trophoblast at the basal plate, but also in some CTB at or near the chorionic plate (Supplementary Fig. S8c). These results suggest that initial state CTB are enriched at/near the basal plate, though some also reside at/near the chorionic plate.

### TSC show some similarities to initial state CTB but have unique gene expression

**Fig. 4:**
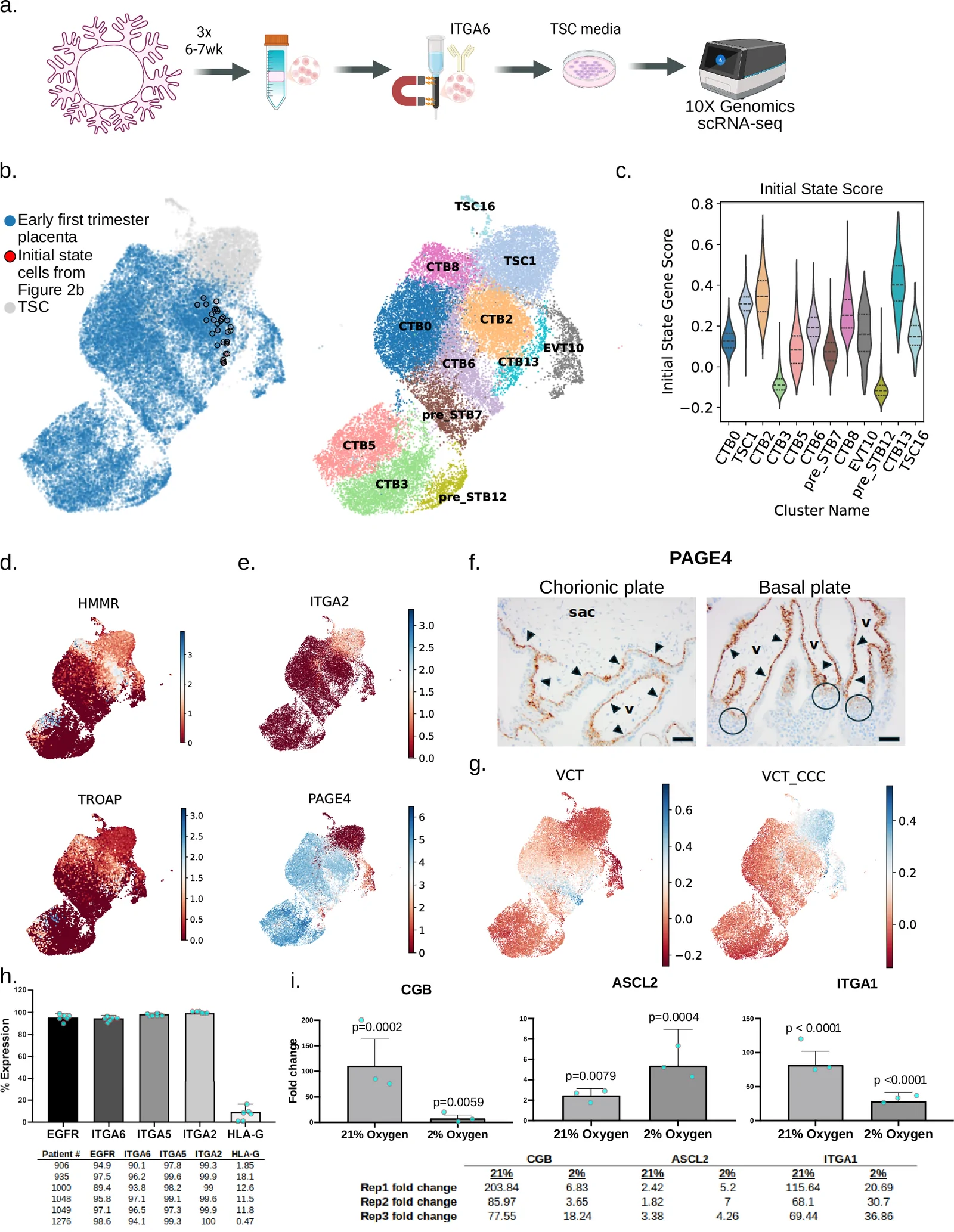
Comparison of TSC to early first trimester trophoblast identifies differences between TSC and initial state CTB. **a** Schematic showing derivation of TSC from three, early first trimester placentas, following CTB isolation by Percoll gradient centrifugation and ITGA6 MACS purification, and passaged in TSC media, then subjected to scRNA-seq. Created in BioRender. Soncin, F. (2026) https://BioRender.com/a1v76tu. **b** UMAP of integrated first trimester trophoblast and TSC (right), annotated based on cell type specific gene expression, as shown in Supplementary Fig. S9d. Location of initial state cells (*n* = 30) determined in Fig. 2b (left) is highlighted using red color and black outline. **c** Violin plot showing the initial state score by cluster, based on expression of genes significantly enriched in initial state cells (Fig. 2b, Supplementary Data 10). Dashed lines show quartiles. **d** UMAPs showing expression of initial state markers *HMMR* (top) and *TROAP* (bottom). **e** UMAPs showing expression of pcEVT marker *ITGA2* (top) and vCTB marker *PAGE4* (bottom). **f** In-situ hybridization of *PAGE4* in a 6-week placenta, showing uniform expression in villous CTB (arrowheads) within the gestational sac (“sac”), as well as in chorionic villi (“v”) near the chorionic and basal plates, but excluded from proximal column trophoblast (circles). Scale bar=125 μm. **g** UMAP visualizations depicting the scores of villous cytotrophoblast (VCT) and cytotrophoblast cell columns derived from VCT (VCT-CCC) cell types based on the top 50 uniquely expressed genes as outlined by Arutyunyan et al.^8^. **h** Percent expression of villous CTB (EGFR and ITGA6), proximal column EVT (ITGA5 and ITGA2), and pan-EVT (HLA-G) markers in undifferentiated TSCs by flow cytometry. Data are reported as mean ± standard deviation of 6 distinct TSC lines. **i** Quantitative PCR of STB marker CGB, and EVT markers ASCL2 and ITGA1, in TSC differentiated in an undirected manner over four days in 21% oxygen or 2% oxygen. Data are presented as fold change over undifferentiated TSC, reported as mean ± standard deviation (*n* = 3, technical replicates), with *p*-values calculated using one-way ANOVA. Table shows fold change values for each replicate.

In 2018, Okae et al. derived trophoblast stem cells (TSC) from ITGA6^+^ CTB fraction of early first trimester (6-8 week gestational age) placentas, and demonstrated their capacity to differentiate into both EVT and STB^7^. Since then, many groups, including ours, have used these or other similarly-derived cell lines, to study various aspects of trophoblast differentiation^32^. We therefore sought to compare these TSCs to various subgroups of early first trimester CTB in order to identify where these stem cells fall along the developmental pseudotemporal order. We used three TSC lines, derived in our lab from early gestation (6–7 week) placentas, and performed scRNA-seq (Supplementary Data 1, Fig. 4a, Supplementary Fig. S9a). Following integration with our early first trimester (6–8 week) placental scRNA-seq data (Supplementary Fig. S9b), we performed clustering and annotated the clusters based on the same markers used to annotate the placental trophoblast clusters (Supplementary Fig. S9c, d, Supplementary Data 12). As TSCs are thought to be similar in function to an initial state CTB, we next identified where in our integrated early first trimester/TSC dataset the previously-determined initial state cells (Fig. 2b) were located (Fig. 4b). Additionally, we scored the cells in our dataset using the initial state marker genes (Supplementary Data 10) and also specifically examined the expression of the previously-identified initial state marker genes *HMMR* and *TROAP*. Our analysis revealed that clusters TSC1 and TSC16, which were predominantly (>90%) comprised of cells from TSC lines, expressed many CTB markers (Supplementary Fig. S9d), had a moderate initial state gene score (Fig. 4c), and did exhibit some expression of initial state markers (*HMMR* and *TROAP*) (Fig. 4d). However, particularly in the case of TSC1 (the larger of the two TSC clusters), our TSC clusters also expressed many EVT markers (Supplementary Fig. S9d); in fact, clusters TSC1 and TSC16 had gene expression profiles most similar to the EVT10 cluster (Supplementary Fig. S9e). To validate the transcriptional identity of our TSC lines, we combined bulk RNA-seq data from four TSC lines (TSCT_1, TSCT_2, Tsblast_1, and Tsblast_2), derived by Okae et al.^7^, with our single-cell dataset. For each bulk sample, we calculated Spearman correlations with the average expression profile of each single-cell cluster and assessed significance using 1000 random gene-label permutations. Three of the four Okae TSC lines showed their strongest correlation with the TSC-enriched cluster TSC1 (r = 0.486–0.555; all permutation *p* < 0.001). The remaining line, TSCT_2, ranked the closely related CTB13 cluster marginally higher than TSC1 (r = 0.489 vs. 0.483; all permutation *p* < 0.001). In UMAP space, the four bulk profiles localized nearest to TSC1 cells based on minimum and median distances, with substantially greater distances to non-TSC clusters (Supplementary Data 13). Together, these findings demonstrate that our independently-derived human TSC lines occupy the same transcriptional neighborhood as the original Okae TSC, reinforcing the conclusion that TSC are transcriptionally distinct from early first trimester CTB.

Differential expression analysis between our TSC clusters and the initial state cells revealed striking differences (Supplementary Data 14). For example, the noncanonical genes *SOX15, SOX9*, and *CD9* were significantly more highly expressed (adj. *p*-value < 1 × 10^−6^ & log fold change > 4) in the TSC clusters relative to our initial state cells (Supplementary Fig. S10a), as were the proximal column trophoblast/EVT progenitor associated genes *ITGA2* and *ITGA5* (Fig. 4e, Supplementary Fig. S10a; adj. *p*-value < 1 × 10^−4^ & log fold change >26). In contrast, the widely-expressed CTB markers, *PAGE4, BCAM, FOXP1, FOXO4, PBX1, PEG3, PEG10, SERPINF1*, and *GPX3*, several of which were identified by Shannon et al. as controllers of CTB-specific regulons, were not expressed in our TSC clusters (Fig. 4e, Supplementary Fig. S10b)^18^. We confirmed by immunostaining that, within first trimester trophoblast, ITGA2 and ITGA5 are confined to HLA-G^+^ cells of trophoblast cell columns within anchoring villi, with rare ITGA2^+^ cells identified only within the proximal column, but absent in villous CTB and STB (CYP19A1^+^) (Supplementary Fig. S10c, d) as previously reported^18,33^. We also performed dual in-situ hybridization for ITGA5 and ITGA2 in first trimester placentas, and confirmed that rare dual-positive cells are only present within the proximal column (Supplementary Fig. S10e). Additionally, we performed in-situ hybridization with a PAGE4-specific probe and validated our scRNA-seq data, showing that this gene has the converse expression pattern as ITGA2 and ITGA5, with expression present only in villous CTB and completely absent in trophoblast cell columns (Fig. 4f).

To identify the placental cell type with the closest transcriptional resemblance to our TSC, we utilized the top 50 cell type marker genes from Arutyunyan et al.^8^ to analyze our integrated early first trimester/TSC dataset. Our analysis revealed that the TSC exhibited the highest similarity to the VCT-CCC cluster (Fig. 4g). These cells, described by Arutyunyan et al.^8^ as a proliferative cell type likely originating from the villous cytotrophoblast (VCT) and giving rise to extravillous trophoblasts (EVTs), are otherwise known as proximal column EVT (pcEVT)^32^. These results suggest that TSCs contain characteristics of progenitor state CTB but have distinct transcriptional programs and may lack the same STB differentiation ability when compared to their villous CTB counterparts.

To more closely evaluate the identity and differentiation potential of our TSCs, we next evaluated the expression of markers of villous CTB (EGFR and ITGA6), pcEVT (ITGA5 and ITGA2), and pan-EVT (HLA-G) in TSCs. We used six TSC lines derived in our lab from 6-8 week gestation placental tissues (including the 3 subjected to scRNA-seq above), performing flow cytometry for all 5 of these markers, and found these cells to almost uniformly express EGFR (95.5 ± 3%), ITGA6 (94.6 ± 2.4%), ITGA5 (98.6 ± 0.8%), and ITGA2 (99.6 ± 0.4%), with a small fraction co-expressing HLA-G (9.4 ± 6.2%) (Fig. 4h). To test whether these markers are retained in TSCs following differentiation, we first performed *directed* differentiation of these cells into EVT or STB, using protocols described by Okae et al.^7^ and performed immunofluorescent staining for ITGA5, along with other markers of differentiation (HLA-G for EVT, and SDC1 and CYP19A1 for STB). We noted that ITGA5 expression was lost following STB differentiation and retained when TSCs were differentiated into EVT (Supplementary Fig. S11a, b). We also evaluated the undirected differentiation potential of TSCs. We had previously shown that culture of first trimester CTB, plated on fibronectin in DMEM/F12 + 10% FBS media, allowed for differentiation of cells into either STB or EVT over 4 days, depending on oxygen tension (21% vs. 2%, respectively)^34^. Therefore, we similarly cultured TSCs for 4 days, and found that, by qPCR, they significantly upregulated STB markers, such as CGA and CGB, in 21% oxygen and EVT markers, such as ASCL2 and HLA-G, in 2% oxygen (Fig. 4i, Supplementary Fig. S11c); interestingly, however, the mature EVT marker, ITGA1, was highest when the cells were cultured in 21% oxygen (Fig. 4i). By morphology and staining, the TSCs formed large SDC1^+^ multinucleated cells in 21% oxygen, but preferentially formed HLA-G^+^ mononuclear cells in 2% oxygen (Supplementary Fig. S11d). These data suggest that, while first-trimester TSCs most closely resemble pcEVT, they do display bipotentiality during directed differentiation (using lineage-specific cues), although their bipotentiality is questionable following undirected differentiation.

### Temporal analysis of TSC derivation shows gradual loss of CTB markers and selection for proximal column EVT markers

**Fig. 5:**
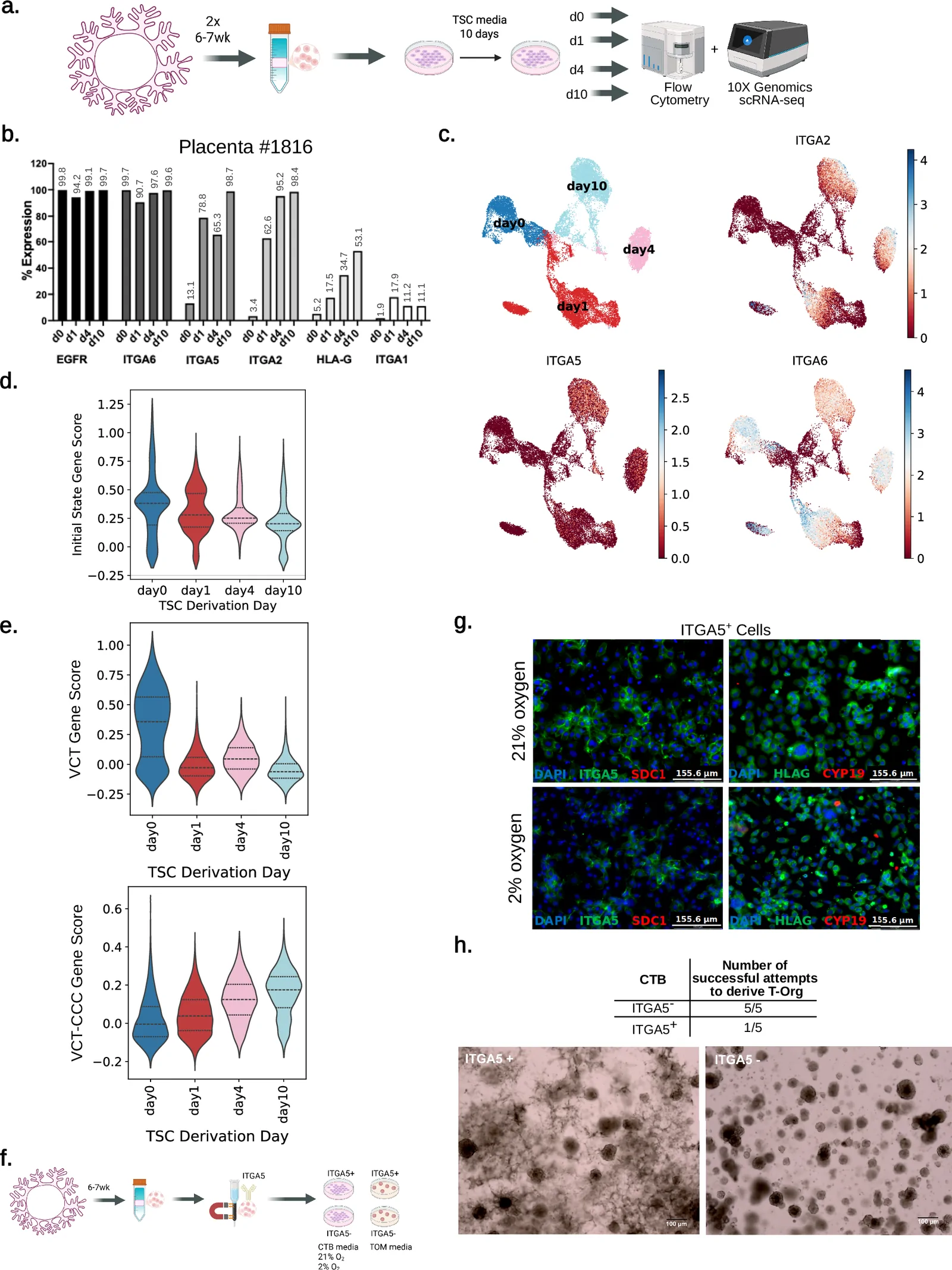
TSC diverge from vCTB and toward a pcEVT phenotype during in vitro derivation. **a** Schematic for data in (**b**) through (**e**), showing early steps of TSC derivation from two first trimester placentas, starting with CTB isolation using Percoll gradient centrifugation, and plating in TSC media. The cells were evaluated throughout a ten-day derivation process in 2D (at isolation on day 0, and at days 1, 4, and 10, the last timepoint being 3 days after the first passage) using both scRNA-seq and flow cytometry. Created in BioRender. Soncin, F. (2026) https://BioRender.com/a1v76tu. **b** Percent of early gestation first trimester CTB expressing markers of CTB (EGFR, ITGA6), pcEVT (ITGA5, ITGA2), pan-EVT (HLA-G) and mature EVT (ITGA1), by flow cytometry, at 4 timepoints during TSC derivation. **c** UMAPs colored by day in TSC medium (top left) and expression of *ITGA2/5/6* in the same placenta as (**b**). **d** Violin plot showing initial state scoring of cells grouped by day of derivation in placenta #1816. Scoring was done using the significantly enriched genes in initial state cells in first trimester placenta (Fig. 2b, Supplementary Data 10). Dashed lines represent quartiles. **e** Violin plots, grouped by day of derivation, scoring cells from placenta #1816 based on the top 50 uniquely expressed genes as outlined by Arutyunyan et al.^8^ for the cell types villous cytotrophoblast (VCT) and cytotrophoblast cell columns derived from VCT (VCT-CCC). Dashed lines represent quartiles. **f** Schematic for data in (**g**) and (**h**), showing first trimester CTB, isolated using Percoll gradient centrifugation, followed by ITGA5 MACS purification. Both the ITGA5^-^ and ITGA5^+^ cells were plated in standard CTB culture media (DMEM/F12 with 10% FBS), cultured in either 21% or 2% oxygen, or in trophoblast organoid media (TOM), cultured under 21% oxygen. Created in BioRender. Soncin, F. (2026) https://BioRender.com/a1v76tu. **g** Primary first trimester CTB, MACS-sorted based on ITGA5 expression, allowed to differentiate undirected over 4 days in either 21% oxygen or 2% oxygen, then fixed and stained for pcEVT marker ITGA5, the EVT marker HLA-G, STB markers SDC1, CYP19A1, and DAPI. Only ITGA5^+^ cells are shown, as ITGA5^-^ cells did not adhere to any substrate in 2D. **h** Organoid (T-Org) formation using first trimester CTB sorted for ITGA5. Table shows number of successful attempts to derive and culture organoids beyond 5 passages. Brightfield images of representative T-Org, derived from ITGA5^-^ and ITGA5^+^ CTB.

To determine how culture conditions affect the expression of CTB and pcEVT markers during TSC derivation, we isolated CTB from first-trimester placental tissues (Supplementary Data 1) and evaluated them throughout a ten-day TSC derivation process in 2D (at isolation on day 0, and at days 1, 4, and 10, the last being 3 days after the first passage) using scRNA-seq and flow cytometry (Fig. 5a). We noted a fast rise in ITGA2 and ITGA5 expression by flow cytometry by day 1 of culture in TSC media (Fig. 5b); this was similarly noted at the RNA level for both of these genes, though it was more subtle for *ITGA5* (Fig. 5c). CTB markers, including *EGFR* and *ITGA6*, remained the same (Fig. 5b, c). These results were confirmed in an additional placental sample using both scRNA-seq and flow cytometry (Supplementary Fig. S12a, b). Next, to determine if our TSC derivation changed in initial state potential throughout the derivation process, we scored each day using the initial state enriched genes from our integrated first trimester placentas. In both patient samples, the initial state score decreased over time and was lowest by day 10 of the derivation process (Fig. 5d, Supplementary Fig. S12c). To identify the placental cell type most closely resembling cells emerging during TSC derivation, we once more evaluated both patient datasets using the top 50 cell type marker genes identified by Arutyunyan et al.^8^. Our data revealed a trend where exposure to TSC culture media corresponded to a decrease in VCT-associated gene expression and an increase in pcEVT-associated (VCT-CCC) gene expression (Fig. 5e, Supplementary Fig. S12d). These results suggest that TSCs derived from the CTB fraction of early first trimester (6–8 week gestational age) placentas quickly lose initial state CTB features and become more similar to EVT progenitor cells.

To define the kinetics and regulatory architecture underlying this transition, we integrated sequential differential expression, pseudotime correlation, transcription factor dynamics, and pathway enrichment analyses. Differential expression between sequential timepoints (adj. *p*-value < 0.05 and log fold change > |1|) demonstrated that the majority of transcriptional remodeling occurred within the first 24 h of culture adaptation. The day 0 → 1 transition yielded 585 differentially expressed genes (334 upregulated, 251 downregulated), compared to 228 genes between day 1 → 4 (72 upregulated, 156 downregulated) and only 46 genes between day 4 → 10 (14 upregulated, 32 downregulated) (Supplementary Data 15). Diffusion pseudotime analysis identified 20 genes negatively correlated with pseudotime (r < –0.3), representing early-state programs that progressively decreased during differentiation, and 126 genes positively correlated with pseudotime (r > 0.3), representing late-state programs that increased over time (Supplementary Data 16). Negatively correlated genes were highly expressed at day 0 and diminished thereafter, whereas positively correlated genes were initially low and increased across differentiation, consistent with a directional transcriptional shift. Transcription factor analysis revealed coordinated regulatory changes along this trajectory. Factors changing early in the CTB-to-TSC transition included AP-1 components (FOS, FOSB, JUN), DNMT1, and PEG3, which were negatively correlated with pseudotime (r < −0.2), while those changing later during the transition included GATA2, GATA3, HES4, ARID3A, and multiple zinc finger transcription factors, which were positively correlated (r > 0.2) (Supplementary Fig. S12e, Supplementary Data 17). KEGG pathway enrichment demonstrated stage-specific functional programs, with early transitions enriched for signaling-related pathways and later stages enriched for trophoblast differentiation-associated processes (Supplementary Data 18). Together, these multilayered analyses demonstrate that TSC derivation is characterized by rapid and coordinated transcriptional reprogramming that aligns with the acquisition of a pcEVT-like gene expression profile.

In order to compare TSCs to their most equivalent counterpart in vivo, we isolated CTB and MACS-sorted for ITGA5 in order to evaluate their differentiation potential. We used this marker instead of ITGA2, since, compared to ITGA2^+^ cells, ITGA5^+^ CTB comprised a larger proportion of isolated mononuclear trophoblast, thus providing sufficient cell numbers from one placenta for one experiment. We first plated the cells in 2D, and found that only ITGA5^+^ cells adhered to the tested substrates (Collagen IV or fibronectin), in both oxygen tensions tested (21% or 2%), in DMEMF12/FBS media, while very few ITGA5^-^ cells did the same (Supplementary Fig. S12f, Fig. 5f). In both oxygen tensions, after 4 days in culture, ITGA5^+^ cells upregulated the EVT marker, HLA-G, but not the STB markers, SDC1 or CYP19A1, while the few adhered ITGA5^-^ cells did not upregulate any markers (Fig. 5g). We next tested the ability of CTB, sorted for ITGA5, to be cultured in 3D and form organoids, and found that only ITGA5^-^ organoids showed the proper morphology and could be consistently maintained beyond 5 passages (Fig. 5h). The majority of the ITGA5^+^ organoids could not be maintained long-term and showed extensive outgrowth formation instead (Fig. 5h). These data show that ITGA5^+^ CTB lack bipotentiality and are unable to form organoids; instead, consistent with their EVT progenitor state, in the absence of lineage-directed cues, they undergo differentiation only into HLA-G^+^ EVT.

## Discussion

Defects in early placental development are thought to be at the root of common pregnancy disorders such as preeclampsia, fetal growth restriction, recurrent miscarriage, and stillbirth. Furthermore, through developmental programming, abnormal early placental development can impact the long-term health of the offspring^35^. Despite the critical role early placental development plays in both reproductive success and overall health, our understanding of the development of this transient but consequential organ remains elementary due to ethical constraints, the absence of comparable animal models, and, until recently, the lack of normal (non-transformed) in vitro cell models. The derivation of TSCs by Okae et al. in 2018 has truly transformed this field, allowing for probing of specific genes and pathways in trophoblast differentiation and function^7,36–41^. However, recently, this model has come under significant scrutiny, particularly in comparison to the 3D trophoblast organoid (T-Org) models^15,16^, with respect to their differentiation potential^18,36^. Here, we focused on 2D-cultured TSCs, derived from 6–8-week gestation placentas, and, using single-cell transcriptomics, comparing them to the heterogeneous population of CTB, in both early (6–8 week) and late (12–14 week) first trimester placentas, and within the basal (maternal) vs. chorionic (fetal) plates in order to identify the cell of origin for these stem cells.

We identified multiple CTB clusters in early and late first trimester placentas, which could be grouped into three broad CTB groups. Two of these (groups 1 and 2) primarily contained CTB from early first trimester, and one (group 3) contained a much higher proportion of late first trimester CTB. Group 3 showed enrichment in genes involved in vascular remodeling, immune regulation, structural roles, and trophoblast differentiation, suggesting a role for these CTB in chorionic villous development, including angiogenesis, during this time period. Of the two early CTB groups, group 2 cells lacked mature CTB markers and were enriched for immune response pathways, consistent with a protective barrier role for such primitive CTB in the implanting embryo^42,43^. The other early CTB group, group 1, appeared to have a gene expression profile more correlated to the late CTB group 3, and contained early bipotential progenitors. These “initial state” CTB in group 1 expressed transcriptional regulators known to regulate gene expression through epigenetic modification, such as *TET1* and *KMT2A*, as well as several cell division and motility-related genes, including *HMMR* and *TROAP*, and were enriched in the basal plate, near the maternal surface. Although functional studies are needed to further characterize this cell population, these data suggest that bipotential CTB exist predominantly in early first trimester (6–8 weeks gestational age) and are enriched at/near the basal plate.

To better understand the differences in CTB composition in the basal plate (BP) and chorionic plate (CP), we performed spatial transcriptomics on early first trimester placentas. These two surfaces are known to perform very different tasks in the placenta, but interestingly, both have been suggested to harbor bipotential trophoblast stem cells^33,44,45^. Not surprisingly, we found significant transcriptional differences between CTB in these two compartments. This suggested that there are important differences in differentiation potential between the CTB found within these two regions and prompted us to compare the CTB contained there at the single cell level. Interestingly, compared to CP-CTB, BP-CTB showed a higher initial-state cell score and had higher expression of the initial state markers *HMMR* and *TROAP*, verified using in situ hybridization. These data indicate that CP-CTB are primarily a source of STB precursors, whereas the BP-CTB are more likely to form EVT but still hold the potential to differentiate into STB. In fact, studies using first trimester villous explant cultures have demonstrated the existence of two distinct CTB subtypes, with CTB at the villous tips (or anchoring columns at the basal plate) representing a more robust progenitor cell (e.g. more resistant to cell death in vitro), and able to give rise to EVT for many days in culture^44^. To our knowledge, ours is the first study to directly compare CTB from the basal and chorionic plates in first trimester placenta, using both spatial and single cell transcriptomics. However, our study did not address the long-standing debate regarding the presence of one single bipotential TSC vs. two distinct (STB vs. EVT) progenitors in the placenta across gestation, nor the heterogeneity of distribution of such cells across different regions of the placenta^44–46^. Future studies, using more samples across both early and late gestational timepoints, are needed to address these important remaining questions.

Recently, several groups have compared TSC to T-Org and in vivo trophoblast^8,14,18,25^, with a focus on TSC grown in 3D. Similar to our results, these studies have also concluded that the TSC state shares transcriptional hallmarks of proximal column, and not villous, CTB, thus predisposing differentiation towards EVT, not STB. Interestingly, we found that compared to the initial state CTB in early first trimester, our TSCs shared significant transcriptional overlap, including expression of the initial state markers *HMMR* and *TROAP*, and the canonical villous CTB markers *EGFR* and *ITGA6*. However, we also found large transcriptional differences between initial state CTB and TSC, with the latter showing upregulation of the cell motility-associated *CD9*, the versatile stem cell-associated marker *SOX9*, as well as the pcEVT-associated genes *ITGA2, ITGA5*, and *SOX15*; of note, *CD9*, *SOX9*, and *ITGA2* were also previously highlighted by Shannon et al. as markers of the TSC state^18,25,47,48^. At the same time, TSCs also showed downregulation of numerous villous CTB genes, including *BCAM, GPX3*, and PEG10, as well as *FOXP1, PBX1*, and *FOXO4*, which Shannon et al. identified as transcription factors driving CTB-specific gene regulons^18^. Another such gene, *PAGE4*, which we confirmed as a vCTB-specific gene by in-situ hybridization, is a stress-response gene with known anti-apoptotic roles in the prostate^49^, but otherwise not well-characterized functionally. In fact, when we scored our integrated TSC and early first trimester trophoblast dataset, using markers from the recently published spatial transcriptomics study by Arutyunyan et al., we found that TSCs appeared to be most similar, not to vCTB, but to their proliferative EVT progenitor cell type VCT-CCC^8^, equivalent to pcEVT. To confirm our scRNA-seq data, we characterized TSC lines from 6 different placentas, and demonstrated that, in fact, all lines uniformly expressed the pcEVT markers *ITGA2* and *ITGA5*. Notably, our TSCs retained the expression of *ITGA5*, only upon differentiation to EVT, but lost it with forskolin-induced differentiation into STB. As noted in previous reports, unlike trophoblast organoids, TSCs do not form STB unless directed using forskolin^14,18^. In fact, when we allowed TSCs to differentiate (by culture in DMEM/F12 + FBS in 21% oxygen), the cells formed large syncytia that were positive for the STB marker SDC1, but also upregulated ITGA1, an EVT marker. ITGA1 has been noted to be increased in human pluripotent stem cell-derived syncytia, and considered to be more representative of invasive primitive syncytium^50^. We therefore suggest that a more detailed evaluation of these TSC-derived multinucleated cells is needed, preferably by single nucleus RNA-seq, in comparison to in vivo STB and primitive syncytium, in order to identify their true cell identity.

It is possible that, similar to first trimester explant culture, where villous CTB quickly undergo cell death while basal plate CTB continue to give rise to EVT outgrowths^44^, current TSC culture conditions favor the stabilization and expansion of an EVT progenitor-like cell type, instead of a more uniformly bipotential vCTB-like cell-type. In fact, in our hands, first trimester CTB cultured in TSC media rapidly and significantly increased the expression of both ITGA2 and ITGA5, markers of pcEVT by flow cytometry. Our single cell transcriptomic analysis of TSC derivation from early first trimester CTB furthers this hypothesis, and suggests that culture in TSC media leads CTB to lose their initial state/transcriptional bipotentiality signature and gain a pcEVT-associated VCT-CCC signature, within the first 10 days of the TSC derivation process. Our temporal transcriptomic analyses further support this model, revealing a rapid early transcriptional shift, marked by suppression of immediate-early AP-1 factors (FOS, FOSB, JUN) and activation of trophoblast lineage regulators (GATA2, GATA3), consistent with stabilization of a proximal column/EVT progenitor-like state under current TSC culture conditions. Furthermore, when we sorted our TSC based on ITGA5 expression, only ITGA5^+^ CTB adhered to matrix-coated plates in 2D, differentiating in an undirected fashion into only EVT-like cells, while ITGA5^-^ cells, could only be cultured in 3D, as T-Org. These data further support the hypotheses put forth above, that TSCs are not only biased toward the EVT lineage, but given their inability to generate bona fide STB in the absence of lineage-directed cues, may therefore not be an appropriate model for true STB differentiation. Instead, T-Org consist of cells that better model villous CTB and form an inner STB layer under standard organoid culture conditions^14,18^. Nevertheless, single nucleus RNA-seq analysis of multinucleated cells, derived from TSC (cultured in 2D or 3D) vs. T-Org, and comparison to in vivo STB and primitive syncytium, are needed, in order to characterize the identity of multinucleated cells across these models.

As with any study of this kind, several technical limitations should be noted. The 10X Chromium platform used in this study is limited in the size of cells it is able to capture. While differential recovery is not considered an issue for cells below 30 µm in this system, this has not been tested for cells above this size. This most likely excluded capture of mature multinucleated STB and trophoblast giant cells. Moreover, our dataset was comprised of cells from a relatively small number of presumably normal placentas, sequenced at a relatively shallow read-depth compared to bulk RNA-seq, potentially constraining our ability to find important but lowly-expressed genes. Finally, trajectory analysis using RNA velocity relies on static snapshots of cellular states at the moment of measurement, and is therefore reliant on only a small number of genes, which appear to obey the simple interpretable kinetics used by RNA velocity^51^. Future studies should use single nucleus RNA-seq, combined with metabolic labeling, and compare 2D and 3D models of trophoblast to multinucleated cells within normal tissue, in order to identify the optimal ways to model these mature cell types.

In conclusion, our findings offer an in-depth transcriptomic perspective of the first trimester placenta and provide insight into early placenta developmental, as well as considerations regarding the utility of TSC as a model system. The identification of distinct CTB populations, especially those located at the basal and chorionic plate, and their developmental trajectories provide a valuable resource for future studies of distinct subtypes of first trimester CTB. The integration of TSC data, with its similarities and differences to in vivo CTB, highlights the potential and limitations of TSCs in recapitulating early trophoblast differentiation. Finally, validation of scRNA-seq data, not just with spatial transcriptomics and in-situ hybridization, but also with primary cell isolation and culture, offers further insights into the actual differentiation potential and functions of distinct CTB progenitor states. Future studies should aim to further elucidate the factors influencing TSC culture and differentiation and develop conditions for culture and characterization of other CTB progenitors within early gestation human placentas.

## Methods

### Patient recruitment and tissue collection

Human placental tissue samples for this study were collected under a UC San Diego Institutional Review Board-approved protocol; all patients gave informed consent for collection and use of these tissues. All samples were collected from pregnant individuals, undergoing elective termination of pregnancy, providing written informed consent. All ethical regulations relevant to human research participants were followed.

Early first trimester placental tissues, including those that were used to derive TSCs and organoids, were taken from patients with gestational ages ranging from 5 weeks and 5 days to 8 weeks and 4 days. Late first trimester placental tissues (*n* = 2) were taken from patients with gestational ages of 12 weeks and 2 days and 13 weeks (Supplementary Data 1). Gestational age was determined based on crown-rump length, measured on ultrasound, and was stated in weeks/days from the first day of the last menstrual period. “Normal” sample was defined as a singleton pregnancy without any detectable fetal abnormalities on ultrasound.

The biological sex of the placental tissues used in this manuscript was not considered in the analyses presented here. The small sample size used in this study prohibited the authors from making meaningful inferences about biological sex differences. Sex determination can be inferred by those using the data in this study by expression of Y chromosome-linked genes.

### Trophoblast sample collection and preparation

Samples were processed within 2 h of collection according to our established protocol^34^. For whole placental samples, including TSC and organoid derivation, chorionic villi were minced and subjected to three sequential enzymatic digestions with Trypsin and DNase, followed by Percoll (Sigma-Aldrich) gradient separation. For single cell analysis, after filtration, cells were counted and loaded on the 10× Genomics chip. For basal and chorionic plate sample collection, we collected the villous tips (basal fraction) and the gestational sac (chorionic fraction) under a dissecting microscope, while the villi in the middle section were discarded. The two fractions were subjected to the same sequential enzymatic digestion and after filtration cells were separated on a Percoll (Sigma-Aldrich) gradient. Cell suspensions were loaded onto the 10× Genomics Chromium Controller for GEM generation. All single-cell libraries were constructed using the Chromium Next GEM Single Cell 3ʹ, Library & Gel Bead Kit v2, v3, and v4 (10× Genomics), following the manufacturer’s protocol for all steps. Single-cell libraries were sequenced on an Illumina NovaSeq 6000 instrument. Capture rate and sequencing depth can be found in Supplementary Data 1. scRNA-seq datasets are publicly available on the GEO repository (GSE270174). Leftover cells were lysed for RNA isolation using NucleoSpin kit (Macherey-Nagel) and proper basal vs. chorion separation was verified by enrichment of *CDX2* (chorionic) and *ASCL2* (basal) expression by qPCR. Libraries were prepared only from sample pairs showing correct marker enrichment.

### Patient-derived trophoblast stem cell establishment, culture, and differentiation to EVT and STB

Following Percoll (Sigma-Aldrich) separation, first trimester (GA 6–8 weeks; *n* = 8) CTB were MACS-purified with either a PE-conjugated anti-ITGA6 antibody (Biolegend #313610, final conc. 0.625 µg/mL) or an APC-conjugated anti-EGFR antibody (Biolegend #352906, final conc. 1 µl of 100 µg/mL per 1 million cells), except for the ten day TSC derivation experiment for which no MACS-purification was used. MACS-purified CTB were then plated for TSC derivation on 6-well plates that were coated with 5 μg/ml of collagen IV for at least 1 hour and cultured in the original Okae media^7^. Once cells lines were established, they were switched to a modified complete media, with addition of 100 ng/ml FGF2, 20 ng/ml Noggin, and 50 ng/ml HGF for maintenance and passaged using TrypLE^7,9,13,52^. Once cell morphology had stabilized (after 6–8 passages), TSCs were evaluated for expression of various surface markers, including EGFR, HLAG, and a panel of integrins by flow cytometry (see below).

Directed EVT differentiation was performed by plating 75,000 cells/well onto a 6-well plate pre-coated with 20 µg/ml fibronectin in EVT differentiation media as described by Okae et al.^7^. On day 3, EVT medium was replaced without NRG1, and Matrigel concentration was reduced to 0.5% until day 5. On day 5, cells were re-plated onto a 6-well plate pre-coated with 20 µg/ml fibronectin using the latter media (without NRG1 and with reduced Matrigel). For immunostaining, cells were fixed on day 6.

Directed STB differentiation was performed by plating 25,000 cells/well onto a 6-well plate pre-coated with 2.5 µg/ml collagen IV in the STB differentiation media as described by Okae et al.^7^. Cells were fixed on day 6 for immunostaining.

For undirected differentiation, two TSC lines (1000 P and 1048 P) were plated at a density of 100,000 cells/well in a 12-well plate coated with 20 µg/ml fibronectin and cultured in Dulbecco’s modified Eagle’s medium/F12 with 10% FBS under 21% O_2_ or 2% O_2_, using an XVIVO X3 Workstation. After 4 days, cells were fixed for immunostaining, or collected for RNA isolation and qRT-PCR.

### Isolation and plating of first trimester cytotrophoblasts

CTB were isolated from the first trimester placental tissues by first mincing chorionic villi, washing them in phosphate-buffered saline (PBS), and subjecting them to three sequential digestions, digestion I: 300 U/mL DNase I (Sigma-Aldrich, St. Louis, MO) and 0.125% trypsin (Gibco, Carlsbad, CA); digestions II and III: 0.25% trypsin (Gibco) and 300 U/mL DNase I. The pelleted cells from the second and third digests were pooled, resuspended in Hanks’ balanced salt solution, and separated on a Percoll gradient^53^. For the plating experiment, one million cells were plated per well in a 6-well plate, coated with 20 µg/ml fibronectin, and cultured in Dulbecco’s modified Eagle’s medium/F12 with 10% FBS, 1× penicillin/streptomycin, and 50 µg/ml gentamicin^53^.

In a separate set of experiments, CTB were sorted for ITGA5 by magnetic-activated cell sorting (MACS), following the manufacturer’s protocol (Miltenyi). ITGA5 positive and negative cells were then plated onto 20 µg/ml fibronectin-coated plates and cultured again in DMEM/F12 with 10% FBS and antibiotics under 21% O_2_ or 2% O_2_, using an XVIVO X3 Workstation. Cells were then fixed for immunostaining.

### Trophoblast organoid derivation and culture

CTBs were isolated from first trimester placental tissues using sequential digestion with Trypsin and DNase, followed by Percoll (Sigma-Aldrich) gradient separation as detailed in the section above^53^. Isolated trophoblast cells were sorted for ITGA5 by MACS with ITGA5-FITC antibody (Biolegend #328008, final conc. 5 µg/mL), following the manufacturer’s protocol (Miltenyi). ITGA5 positive and negative cells were counted and plated into Phenol Red free Matrigel (Corning) domes at 37,000 cells/15ul domes in trophoblast organoid media (TOM) comprised of Advanced DMEM/F12 (Life Technologies), 1× N2 and 1× B27 supplements (Life Technologies), 2 mM Glutamax (Life Technologies), 1.25 mM N-Acetyl-L-cysteine (Sigma), 500 nM A83-01 (Tocris), 1.5 μM CHIR99021 (Sigma-Aldrich), 80 ng/mL human R-spondin 1 (Peprotech), 50 ng/mL human EGF (R&D), 50 ng/mL human HGF (Stemcell Technologies), 2.5 μM prostaglandin E2 (PGE2) (Selleck Chemicals), 100 ng/mL human FGF2 (BioPioneer), and 2 μM Y-27632 (Selleck)^15^. Cultures were maintained in a 37 °C humidified incubator with 5% CO_2_ and media changed every 2–3 days. After 10–12 days, organoids were passaged following mechanical dissociation as described in Turco et al.^15^. After the first passage, organoids were maintained in TOM media and passaged with a combination of TryPLE Express (Thermo Scientific) treatment for 3–5 min. followed by mechanical dissociation every 10–14 days and re-seeded at 20,000 cells/20 μL in Matrigel dome^54^.

### Flow cytometry analysis

Isolated CTB and derived TSCs were stained, either individually with EGFR-APC (Biolegend #352906, final conc. 1 µl of 100 µg/mL per 1 million cells), HLAG-PE (ExBIO #1P-292-C100, final conc. 2.5 µg/mL), ITGA6-AF647 (Biolegend #313610, final conc. 0.625 µg/mL), ITGA5-FITC (Biolegend #328008, final conc. 5 µg/mL) and ITGA2-AF594 (R&D #FAB1233T, final conc. 5 µg/mL), or using a multi-color panel of EGFR-PE/Cy7 (Biolegend #352910, final conc. 2.5 µg/mL), HLAG-APC (ExBIO #1A-292-C100, final conc. 2.5 µg/mL), ITGA1-PE (Biolegend #328304, final conc. 5 µg/mL), ITGA2-AF700 (R&D #FAB1233N, final conc. 5 µg/mL), ITGA5-FITC (Biolegend #328008, final conc. 5 µg/mL) and ITGA6-BV421 (Biolegend #313624, final conc. 1 µl of 25 µg/mL per 1 million cells), with relative isotype control antibodies, for 1 hour in flow cytometer wash buffer (1% FBS, 2%BSA and 0.03% Sodium Azide in PBS) in the dark. After staining, cells were washed three times with wash buffer and fixed with 2% PFA, and samples were acquired on either a FortessaX20 or Fortessa (BDLSRFortessa) flow cytometer (BD Biosciences). Data analysis was performed using FlowJo and data were represented as bar graphs using GraphPad Prism.

### Immunofluorescent staining of cells and tissues

Cells were fixed with 4% paraformaldehyde (PFA) in PBS for 10 min, washed once with PBS, then permeabilized with 0.1%Triton X for 10 min at room temperature (RT), and washed three times with PBS. Cells were blocked with 5% Goat serum and 0.05% Triton X in PBS for 1 h at RT. Cells were stained overnight at 4 °C with primary antibodies, including mouse anti-SDC1 (0.5 µg/ml, Abcam # ab34164), rabbit anti-ITGA5 (1.1 µg/ml, Abcam, #ab150361), and mouse anti-CYP19A1 (1.0 µg/ml, Novus Biologicals, #NBP3-07826), and Rabbit anti-HLAG (0.5 µg/ml, Abcam #ab283260), followed by secondary antibodies (Alexa Fluor-conjugated goat anti-rabbit or anti-mouse antibodies, Thermo Fisher Scientific) for 1 h at RT in the dark. Nuclei were stained with DAPI, and images were taken on a Leica fluorescence microscope.

Immunofluorescent staining was also performed on formalin-fixed paraffin-embedded (FFPE) placental tissues. 5-mm serial sections were cut, dried, and deparaffinized and rehydrated using xylene and ethanol. Sections were permeabilized in a 0.1% triton solution in 1× TBS for 10 min, then subjected to antigen retrieval in EDTA at 100 °C for 15 min. Tissue sections were blocked using 10% normal goat serum (Jackson ImmunoResearch, Cat# 005-000-001) and 5% BSA (Gemini BioProducts, Cat#700-110) in TBST for 30 min at 37 °C. Serial sections were stained with mouse anti-HLAG (10 µg/mL, Abcam, #ab52455), rabbit anti-ITGA2 (1.0 µg/mL, Bioss, #bsm-52613R) and rabbit anti-ITGA5 (1.1 µg/ml, Abcam, #ab150361) and incubated at 4 °C overnight. The following day, slides were washed with 1× TBS then incubated with Alexa Fluor 488 and 594 conjugated goat anti-rabbit and goat anti-mouse (Thermo Fisher) for 1 h at room temperature covered from light. Slides were then washed in a 1:5000 dilution of DAPI in 1× TBS for 7 min, washed in 1× TBS, then mounted using Prolong Gold (Invitrogen). Images were acquired using a Leica fluorescence microscope and individual channel photos were merged using ImageJ.

### In situ hybridization

First trimester placental tissue samples were fixed in a neutral-buffered formalin and embedded in paraffin wax. Five sections were subjected to in-situ hybridization on a Ventana Discovery Ultra automated stainer (Ventana Medical Systems) at the UC San Diego Advanced Tissue Technology Core laboratory. RNAscope probes specific to human *PAGE4, HMMR, TROAP, ITGA5*, and *ITGA2* were purchased from ACD-Bio. Following amplification steps, the probes were visualized using an HRP-based reaction, visualizing single probes (*PAGE4, HMMR*, and *TROAP*) in brown and dual probes in teal (*ITGA5*) and red (*ITGA2*); slides were counterstained with hematoxylin. Slides were visualized by conventional light microscopy on an Olympus BX43 microscope (Olympus).

### GeoMx digital spatial profiler whole transcriptome assay

For spatial transcriptomic analysis, three 6-week gestation placentas, previously formalin-fixed and paraffin-embedded, were selected from the Center for Perinatal Discovery’s biorepository (Supplementary Data 1). Samples containing a visible, relatively intact embryonic sac were selected so that the basal and chorionic areas were easily identifiable. 5μm sections were mounted on SuperFrostTM Plus slides and used within 2 weeks. Sections were deparaffinized and incubated overnight with the Whole Transcriptomic Assay UV-cleavable biological probes according to manufacturer’s instructions. Samples were stained with CONFIRM® anti-EGFR (5B7) Rabbit Monoclonal Primary Antibody (Ventana #790-4347, 1:20 dilution) and anti-HLA-G (4H48) mouse primary antibody (Abcam #ab52455, 1:1000 dilution) for 2 h followed by secondary staining for 1 h with Alexa Fluor anti-rabbit-647 (Life Technologies, 1:500 dilution), Alexa Fluor anti-mouse-594 (Life Technologies, 1:500 dilution), and SYTO13 (Nanostring, 1:10 dilution) for nuclei staining. Slides were scanned on a GeoMx digital spatial profiler (Nanostring). Based on the morphology markers, regions of interest (ROIs) containing at least 100 EGFR^+^/HLA-G^-^ nuclei were manually selected. In total, we identified 29 ROIs, 14 from the basal plate and 15 from the chorionic plate. Samples were collected and libraries prepared according to the manufacturer’s instructions. Libraries were pair-end sequenced on a NovaSeq 6000 in the UCSD IGM core at 3 million reads per sample. Fastq files were converted into DCC files following the GeoMx NGS pipeline (v.2.0.0.16). Data QC was performed on the GeoMx DSP software according to standard parameters (minimum 100 nuclei per ROI, area size at 1000µm2, negative probe count to 1, sequencing saturation at 30, Q3 normalization) and then exported for further downstream analysis. Genes were considered differentially expressed between the basal and chorionic plate using Mann–Whitney U test (adj. *p*-value < 0.05 & log fold change >1), (Supplementary Data 11). Data are publicly available on the GEO repository (GSE270174).

### RNA isolation and qRT-PCR

On day 4, cells were collected, and RNA isolated using NucleoSpin® (Macherey-Nagel, USA) kit. 300 ng of RNA was reverse transcribed to prepare cDNA using PrimeScript™ RT reagent kit (TAKARA, USA) following the manufacturer’s instructions. qRT-PCR was performed using Power SYBR® Green RT-PCR Reagents Kit (Applied Biosystems, USA) using the following primer pairs in a 5’ to 3’ orientation: ASCL2 forward CACTGCTGGCAAACGGAGAC, ASCL2 reverse AAAACTCCAGATAGTGGGGGC; CGB forward ACCCTGGCTGTGGAGAAGG, CGB reverse ATGGACTCGAAGCGCACA; ITGA1 forward CTGGACATAGTCATAGTGCTGGA, ITGA1 reverse ACCTGTGTCTGTTTAGGACCA; L19 forward AAAACAAGCGGATTCTCATGGA, L19 reverse TGCGTGCTTCCTTGGTCTTAG. HLA-G forward ACTGAGTGGCAAGTCCCTTT, HLA-G reverse TGGGGAAGGAATGCAGTTCAG; CGA forward CAACCGCCCTGAACACATCC, CGA reverse CAGCAAGTGGACTCTGAGGTG. Data were normalized to L19, analyzed according to the delta delta Ct method and shown as fold-change over undifferentiated TSC. GraphPad Prism was used to perform statistical analysis. Ordinary One-way ANOVA with multiple comparison was used to calculate the statistical analysis related to with undifferentiated TSCs. Data is expressed as mean ± standard deviation of 2^-ddCt values. The level of statistical significance was set at *p* < 0.05.

### Trophoblast scRNA-seq library construction and sequencing

Cells were run on the 10X Genomics platform with the Chromium Next GEM Single Cell 3’ kit. Samples and libraries were prepared following the manufacturer’s protocol to attain 5000–10,000 cells per reaction. Libraries were pooled and sequenced using the Illumina NovaSeq 6000 sequencer. Capture rate and sequencing depth can be found in Supplementary Data 1.

### Single-cell RNA-seq data analysis: data mapping and technical artifact removal

All scRNA-seq data was mapped using the STARsolo function of STAR (v.2.7.10a)^55^. STARsolo performed error correction and demultiplexing of cell barcodes using user-input whitelist (3M-3pgex-may-2023.txt for version 4, 3M-february-2018.txt for version 3, and 737K-august-2016.txt for version 2 of the 10X Genomics Chromium System), mapped reads to the reference genome (GRCh38, version 32, Ensembl 98), deduplicated and error corrected UMIs, quantified per-cell gene expression, and spliced and unspliced reads similar to Velocyto. The following are the CellRanger mimicking commands used: --outFilterScoreMin 30 --readFilesCommand zcat --soloCBmatchWLtype 1MM_multi_Nbase_pseudocounts --soloUMIfiltering MultiGeneUMI_CR --soloUMIdedup 1MM_CR --soloCellFilter EmptyDrops_CR --soloUMIlen 12 --soloType CB_UMI_Simple --soloCBwhitelist 3M-february-2018.txt --clipAdapterType CellRanger4 --outFileNamePrefix name --outSAMattributes All --outSAMtype BAM SortedByCoordinate --quantMode GeneCounts --soloCBlen 16 --soloCBstart 1 --soloUMIstart 17 --soloFeatures Gene GeneFull SJ Velocyto. Following STARsolo quantification, the data was run through CellBender’s (v. 0.3.0) ‘remove-background’ command^19^ to remove systematic biases and background noise due to ambient RNA molecules and random barcode swapping.

### Single-cell RNA-seq data analysis: data pre-processing, filtering, integration, and quality control

The data was then loaded into Scanpy (v. 1.10.1)^22^. for preprocessing, filtering, and quality control. Doublet removal was performed by Scrublet^20^. with an expected doublet rate of 0.076. Following doublet filtering, cells with less than 200 genes expressed were removed and genes detected in less than 3 cells were removed. Next, cells with greater than 20% mitochondrial DNA content were removed as well as cells with greater than 5% ribosomal gene expression or 15% hemoglobin gene expression. The data was then normalized, and the cell cycle was regressed out using the cell cycle genes from Macosko et al.^56^, and highly variable genes were selected. If data was specified as being integrated, it was done using scvi-tools (v. 1.1.2)^21^, specifically using the scVI (single-cell Variational Inference) model with “batch” and “cell_source” as the categorical covariate keys. Integration was performed before clustering but after combining all datasets being analyzed and performing the above quality control steps.

### Single-cell RNA-seq data analysis: clustering and clustering annotation

Integrated datasets were clustered using Scanpy’s Leiden clustering algorithm, an improved version of the Louvain algorithm^57^, with a resolution of 0.75 unless otherwise specified. Marker genes were calculated using default parameters and clusters were annotated as trophoblast if they expressed all or some of the genes *KRT7, GATA3, PAGE4, TFAP2A, HLA-G*, or *CYP19A1* and lacked all or most of the expression of the genes *HLA-A, HLA-B, HLA-DRA, CD14, VIM*, or *CD34*. The non-trophoblast clusters were then removed from downstream analyses and the remaining trophoblast clusters were re-clustered using the same resolution after the nearest neighbors distance was re-calculated and embedded using UMAP. Trophoblast clusters were annotated using the following gene expression: CTB (*BCAM, SLC6A4, PARP1, EGFR, TP63, SLC27A2*, and *TENM3*); EVT (*HLA-G, ASCL2, ITGA5, FN1, LAIR2, NOTUM, LPCAT1, PRG2*, and *AOC1*); Pre-STB (*CYP19A1, ERVV-1, GREM2, ERVFRD-1, LGALS16, EPS8L1*, and *SERPINB2*). Hierarchical clustering using Pearson correlation method with complete linkage was used to assess the relative distance between clusters. Differential expression between two groups was performed using ‘rank_genes_groups’ and setting one cluster as the reference and the other as the ‘group’. Genes were considered up- or downregulated using a log2 fold change of 1.5 unless otherwise stated. To compare the number of cells from 6-week and 13-week placentas in each cluster, a size factor calculation was performed by dividing the total number of 6-week or 13-week cells by the total number of cells. The number of cells in each cluster for each GA were then multiplied by this size factor.

### Single-cell RNA-seq data analysis: reanalysis of previously published scRNA-seq from early and late first trimester placenta and bulk TSC RNA-seq

Processed scRNA-seq datasets from seven (four early and three late first trimester) placentas from Shannon et al.^25^ were downloaded from NCBI’s GEO database (GSE174481). The data was filtered, processed, clustered, and annotated using the same methods and filtering cutoffs stated above for our own scRNAseq data. The data were integrated in the same manner, and differential expression was performed between clusters CTB4 (early) and CTB6 (late), as stated above.

Raw bulk RNA-seq counts from four human TSC lines (TSCT_1, TSCT_2, Tsblast_1, and Tsblast_2; JGA: JGA00000000117 and JGA00000000122) were downloaded from Okae et al.^7^ and imported into AnnData format. Genes were intersected with those present in our single-cell dataset, and analyses were restricted to highly variable genes identified in the single-cell data. Both datasets were normalized to a total count of 10,000 per sample and log-transformed (log1p). The normalized matrices were concatenated, scaled, and subjected to PCA, neighborhood graph construction, and UMAP embedding. For similarity analysis, average expression profiles were computed for each Leiden cluster from the single-cell data. Spearman correlations were calculated between each bulk sample and each cluster-average profile. Significance was assessed using a one-tailed permutation test (1000 permutations), in which bulk gene expression values were randomly permuted to generate a null distribution of correlation coefficients. Empirical p-values were calculated as the fraction of permuted correlations greater than or equal to the observed value. In addition, Euclidean distances between bulk samples and individual single cells were computed in UMAP space to assess spatial proximity to specific clusters.

### Single-cell RNA-seq data analysis: trajectory inference and initial state determination

Trajectory inference was performed using scVelo (v. 0.3.2), an RNA velocity^58^ analysis toolkit for single cells that leverages splicing kinetics to recover directed dynamic information using an expectation-maximization framework^26^. or a deep generative model^59^. A function from https://github.com/JBreunig was used to put together all the spliced and unspliced data before preprocessing and moments calculations were performed. Velocities were estimated in a gene-specific manner using the stochastic mode for all trajectories except for the individual patient trajectories, which used the dynamical mode.

To compute the initial state, we used CellRank2 (v. 2.0.4)^27^, a Markov state modeling framework for cellular dynamics, and the velocity kernel, which computes a transition matrix based on RNA velocity, along with the Generalized Perron Cluster Analysis algorithm (GPCCA estimator)^28^. When computing the initial state for the dataset containing the combined early- and late placental samples, first the velocity kernel and connectivity kernel were combined in a 0.8/0.2 ratio. Then using the GPCCA estimator, the terminal states were set to the EVT and pre-STB clusters and then the number of states was selected to be four. From these four states, the initial state was predicted.

### Single-cell RNA-seq data analysis: temporal transcriptomic analysis of TSC derivation using scRNA-seq

Single-cell RNA-seq data from TSC derivation timepoints from both patient samples (patients 1815 and 1816) was filtered, preprocessed, and clustered as discussed above using Scanpy (v1.9.3)^22^. Differential gene expression analyses between sequential timepoints (day 0 vs day 1, day 1 vs day 4, and day 4 vs day 10) were conducted using sc.tl.rank_genes_groups, grouping by day and using the adjacent timepoint as reference. Genes with adjusted *p*-value < 0.05 and log fold change > |1| were considered significantly differentially expressed. Log fold-change thresholds (|logFC|>0.3) were applied for temporal classification analyses. To model continuous differentiation dynamics, diffusion maps were computed using sc.tl.diffmap (15 components). Diffusion pseudotime (DPT) was calculated using sc.tl.dpt with 10 diffusion components, designating a day 0 cell as the root of the trajectory. For each gene, Spearman correlation between normalized gene expression and diffusion pseudotime values was calculated using scipy.stats.spearmanr. Genes with correlation < –0.3 were classified as early (decreasing over pseudotime), and genes with correlation > 0.3 were classified as late (increasing over pseudotime). A curated list of human transcription factors (HumanTF database; http://humantfs.ccbr.utoronto.ca) was intersected with expressed genes in the dataset. Spearman correlations between transcription factor expression and pseudotime were computed as described above to identify temporally regulated regulatory factors. Transcription factors with |correlation|> 0.2 were considered dynamically associated with differentiation. Functional enrichment analysis was performed using GSEApy (v1.1.11) (Enrichr interface). Gene lists (up to 500 genes per category) derived from pseudotime-defined early and late groups, as well as discrete timepoint-specific gene sets, were tested against the KEGG 2021 Human gene set library. Pathways with adjusted *p*-value < 0.05 were considered significantly enriched. To integrate discrete and continuous analyses, genes identified from pairwise differential expression and pseudotime correlation were combined into a unified dataset. Genes were further classified as immediate (day 0 → 1), mid (day 1 → 4), or late (day 4 → 10) responders based on statistical significance (adjusted *p* < 0.05) and |logFC| > 0.3.

### Statistics and reproducibility

Data are reported as mean ± standard deviation. Statistical analyses were performed using GraphPad Prism. Ordinary one-way ANOVA and multiple comparison corrections were used to calculate the statistical significance compared to undifferentiated TSC. qPCR data were analyzed following the ddCt method: data were normalized on the housekeeping gene L19 and expressed as fold change (2^-ddCt^) over undifferentiated TSC. The level of statistical significance was set at *p* < 0.05. The total number of replicates for each flow cytometry and qPCR experiment was between one and six biological replicates and is listed in the accompanied figure legend. Replicates represent independent biological replicates, separate cell lines or patient samples. For qPCR experiments, each reaction was performed in technical triplicate. For scRNA-seq statistical analyses, please refer to the applicable section in the methods details.

### Reporting summary

Further information on research design is available in the Nature Portfolio Reporting Summary linked to this article.

## Supplementary information

Transparent Peer Review file

Supplementary Information

Description of Additional Supplementary Files

Supplementary Data 1-20

Reporting summary

## Data Availability

Single-cell RNA-seq data and GeoMx digital spatial profiler data have been deposited at GEO and are publicly available as of the date of publication. Accession numbers are listed in the reporting summary (GSE270174). Processed scRNA-seq datasets from Shannon et al.^25^ were downloaded from NCBI’s GEO database (GSE174481) and processed scRNA-seq from Arutyunyan et al.^8^ were downloaded from https://www.reproductivecellatlas.org/mfi.html for donor P13. Microscopy data reported in this paper will be shared by the lead contact upon request. This paper does not report original code. The code used for data processing and analysis has been deposited at https://github.com/remorey/Placental_Single_Cell_Analyses and is publicly available as of the date of publication. Source data underlying graphs in figures are either shown below graph, available in Supplementary Data 19, or in the case of scRNA-seq, GeoMx, and Flow Cytometry gating plots, have been deposited at https://github.com/remorey/Placental_Single_Cell_Analyses and is publicly available as of the date of publication. Details of all antibodies used, including manufacturer, catalog number, and concentration, are provided in Supplementary Data 20. Any additional information required to reanalyze the data reported in this paper is available from the lead contact upon request. This study did not generate new unique reagents. Requests for further information, reagents, and human trophoblast stem cell lines used in this study should be directed and will be fulfilled by the Lead contact, Mana Parast (mparast@health.ucsd.edu).
