# Supplementary material for: Single-cell transcriptomics reveals differences between chorionic and basal plate cytotrophoblasts and 2D-cultured trophoblast stem cells
Source: Commun Biol. 2026 Jun 10;9:1234. doi: 10.1038/s42003-026-10460-0 (PMC13594312; doi:10.1038/s42003-026-10460-0)
Supplement: Supplementary file 1 [file 42003_2026_10460_MOESM1_ESM.pdf]

nature portfolio

Peer Review File

Single-cell transcriptomics reveal differences between chorionic and basal plate cytotrophoblasts and 2D-cultured trophoblast stem cells

Corresponding Author: Dr Mana Parast

This file contains all reviewer reports in order by version, followed by all author rebuttals in order by version.

Version 1:

Reviewer comments:

Reviewer #1

(Remarks to the Author)
The authors have addressed all of my concerns through their explantation in the rebuttal letter and by adding additional supplementary figures. I do not have any major concern about the study.

Reviewer #2

(Remarks to the Author)
Point # 2 In my earlier review I commented on the use of the phrase "spontaneous differentiation". The authors have responded to this and they give what they think is an analogy and they state, "as an analogy, mouse TSC, upon removal of the maintenance factors FGF4 and MEF-conditioned media (or activin A), spontaneously differentiate into both labyrinthine and junctional zone trophoblast." This is in fact a perfect example that the use of the word spontaneous is incorrect. The authors acknowledge that the differentiation from stem cells requires the removal of FGF4 and conditioned media. Hence it is not spontaneous - it is induced by the intervention of removing these factors. Therefore, my comment in my earlier review remains - it is inappropriate to use the word "spontaneous" when the observed change requires the addition or removal of a factor or stimulus. Hence the wording still needs to be changed.

Points # 3,4 and 5 I agree, the revised wording clarifies this.

Point #6 This was my major concern – the very low number of samples analysed and the strength of the conclusions drawn. The authors have added new analyses of other data sets to support their work. These are useful additions and are reassuring as they do broadly support the conclusions drawn. Nonetheless I would still like the conclusions to be asserted more cautiously. For example these phrases in the abstract and discussion need tempering: "We found notable distinctions" (line 31); CTB progenitors were enriched…. basal plate CTB were biased toward EVT…" (line35-6); "in-depth transcriptomic perspective….. have significant implications….." Line 521-2);

Point #7 Simply pointing out that n=5 does not address this. Lines 190-1 explicitly state, "We confirmed that these cells were more numerous…." To conclude they were "more numerous" you need to count them in several samples (5 would probably be sufficient) and then asses the apparent difference statistically. I suspect the authors have not done this so softening the hard conclusion to "appeared to be more numerous" would solve the problem – but of course make the conclusion less interesting.

Point #8 ok

Point #9 The use of the phrase "transcriptionally similar". My point is somewhat pedantic and just because other papers use the phrase somewhat loosely is not a justification for propagating the error. Precision matters in science and RNAseq does not measure transcription – it measures the level of transcripts in a sample (or cell). Clearly this level critically depends on transcription but this is not measured – only inferred. I appreciate it that the authors have revised the wording accordingly.

Reviewer #3

(Remarks to the Author)
The authors have addressed most of the concerns. However, several minor issues still need to be further clarified or corrected.

1.Figures 1a, 3a, and 4a have not been cited in the main text.
2.In Tables S5 and S9, does “CTB1–3” refer to CTB groups 1–3 or clusters 1–3? The terminology is currently confusing.
3.In lines 184–185, TET1, KMT2A, and CENPA are not transcription factors. Similarly, in Figure S12e, several listed genes are also not transcription factors.
4.In Figure S4b, what does “CTB-Clade” refer to?
5.The placements of panel label “b” in Figure 3 and panel labels “c”, “g”, and “h” in Figure 5 are not appropriate.
6.In line 352, “Figure S11b” may be incorrect. Should this instead refer to Figure 12b?

Open Access This Peer Review File is licensed under a Creative Commons Attribution 4.0 International License, which permits use, sharing, adaptation, distribution and reproduction in any medium or format, as long as you give appropriate credit to the original author(s) and the source, provide a link to the Creative Commons license, and indicate if changes were made.
In cases where reviewers are anonymous, credit should be given to 'Anonymous Referee' and the source.
The images or other third party material in this Peer Review File are included in the article’s Creative Commons license, unless indicated otherwise in a credit line to the material. If material is not included in the article’s Creative Commons license and your intended use is not permitted by statutory regulation or exceeds the permitted use, you will need to obtain permission directly from the copyright holder.
To view a copy of this license, visit https://creativecommons.org/licenses/by/4.0/

Reviewers' comments:

Reviewer #1 (Remarks to the Author):

The manuscript by Robert Morey and Coauthors focused on single-cell genomics to identify heterogeneity in gene expression patterns in Cytotrophoblast (CTB) cells of the early gestation human placenta and human trophoblast stem cells. Authors made the following major findings (1) Gene expression program in CTBs varies with gestation, (2) CTB progenitors in basal plate have gene expression bias toward EVT precursors, and CTB progenitors in chorionic plate have gene expression bias toward STB precursors and (3) Human TSCs have gene expression pattern more similar to VCT-CCC (the proximal column EVT precursors). Authors also indicated that ITGA5 positive CTBs could be directly differentiated towards EVTs in 2D culture, whereas ITGA5(-) CTBs fail to do so. The manuscript suggest that distinct CTB states exist in different regions of the placenta as early as six weeks gestation and that established TSC lines most closely resemble ITGA5+ CTB, biased toward the EVT lineage. The study has reiterated findings from several prior studies and seems very descriptive. There are major gaps in the manuscript and some of the data is confusing. Following comments should help the manuscript.

1. The manuscript at its present form is descriptive and many of the information are reiteration of earlier findings, such as different subtypes of CTBs exist is well known from other studies. It is also well known that CTBs differ in their gene expression patterns across gestation. Thus, the advancement to our knowledge is only incremental. More mechanistic questions, such as why early first-trimester (6 weeks) vs. late first trimester CTBs (12-13 weeks) differ significantly in gene expression programs (such as loss of mitotic genes) or how they differ in epigenetic programming could have generated more interest to the field.

Response: This is the first study to compare, not just CTB populations between early and late first trimester, but also CTB in different regions of the placenta (chorionic vs. basal plate) at the single cell level, using both 10X and spatial transcriptomics. The impetus for this comparison was to compare TSC and their cells-of-origin, namely, CTB from early first trimester (<10 week gestation) placentas. Therefore, we set out to compare the CTB subpopulations at extremes of this gestational age range (6-8 weeks vs. 12-14 weeks) and identified a late first trimester CTB cell population that contained genes known to play regulatory roles in the placenta such as angiogenesis, immune regulation, structural integrity, and cellular differentiation as well as two early first trimester CTB populations that were enriched for pathways related to cell proliferation, differentiation, KRAS signaling, and immune response. Additionally, unlike previous studies that have focused on TSC cultured in 3D (as trophoblast organoids), this is the first study to compare TSC ***cultured in 2D*** to *in vivo* CTB at the single cell level; as such, we have changed the title to further reflect this distinguishing feature of our study.

2. Authors indicated that a fraction of initial stage CTBs (more in 6 weeks and less in 13 weeks) express mitotic markers and they are mostly confined at the Basal Plate (BP) region. The whole claim is based on studies with only one gene, HMMR. How about looking at mitosis itself? They should perform additional analyses (like phosphohistone H3 and other cell cycle markers) to define heterogeneity of mitotically active/proliferating CTB populations. It is also surprising to see so less number of CTBs expressing HMMR around the chorionic plate (CP) region. Is the CP region CTBs not proliferating at 6 weeks? The possible causes of heterogeneity of distribution of initial stage CTBs should have been included in the discussion.

Response: It is well-known that proliferative CTB decrease in number through gestation (Lee et al. Hum Pathol. 2007; PMID: 17397906) and are enriched at the basal plate (Lee et al. Development. 2018; PMID: 29540503). Although stem cells are by definition proliferative, our study was focused on identifying ***"initial state" CTB***, as a possible source of TSC, using computational methods, and not simply

mitotically-active CTB. Our analysis led to the identification of a CTB subcluster (of 30 cells); we then sought to identify the genes most specifically expressed in these 30 cells, compared to other primary CTB (Supp. Table 10) and our TSC (Supp. Table 13), and found that these cells were defined by a group of genes, including HMMR and TROAP. Moreover, we also show the expression of the transcription factors MXD3 and CENPA in this initial state cell population (Figure S3A). We have now further annotated the ISH figures for HMMR and TROAP (Supp Fig 7c), which are consistently enriched in early first trimester samples, and at the basal plate. It is also important to point out that HMMR and TROAP have roles beyond the cell cycle: they are also known to be involved in cell motility and cell transformation/metastasis (PMID: 1376732, 24367099, 21940500, 36750558, 39990663). HMMR is known to play a significant role in cancer stem cells, where it promotes self-renewal and differentiation through EMT and activating signaling pathways like TGFβ/Smad2 (PMID: 30802150, 24710409). Additionally, TROAP is also highly expressed in cancer stem cells and has been shown to promote not just proliferation but also migration and invasion (PMID: 34077623, 33500384). We now also mention the heterogeneity of distribution of these "initial state" CTB in the discussion.

3. The region of interest for spatial transcriptomics for basal plate (BP) region is biased as authors mostly selected anchoring villi regions (Fig. S4B), which contain a lot of proximal column CTBs. Authors should also select regions beyond anchoring villi. Otherwise the results that the BP region contains more initial stage CTBs as well as it contains more EVT precursors are contradictory claims. The comparison of spatial transcriptomics with scRNA-seq is also biased as it only considers the selectively enriched genes.

<u>Response</u>: Within a dilation & curettage (D&C) specimen, which describes all of our first trimester placenta samples, the only way to recognize the basal plate is based on morphology of the anchoring villi, in the same way that the recognition of the chorionic plate depended on identification of the sac. However, for spatial transcriptomics, we used EGFR immunostaining for segmentation: in other words, within the "regions of interest" or ROI, we selected only the EGFR+ cells in the anchoring villi, in order to profile the CTB, and not proximal column trophoblast, from these regions. Our spatial data revealed that there are differences between EGFR+ CTB in the BP and CP regions of the placenta, showing that genes enriched in the BP-CTB were more highly expressed in the EVT cluster (EVT9) of our whole placenta single cell data (early and late first trimester placentas; Figure S4d).

The fact that we found differences between BP and CP in our spatial data prompted us to perform scRNA-seq on CTB isolated from dissected CP (sac) and BP (anchoring villous tips) regions of two early first trimester placentas, using Percoll gradient centrifugation. We then used the initial state CTB gene expression profile from our combined early and late first trimester whole placenta dataset to locate cells with a similar gene expression profile in our BP/CP dataset, and found that the initial state signature genes were most highly expressed in the CTB1 cluster, which is enriched in basal plate cells (Figure 3c-d). Thus, the conclusion that the BP region contains both more EVT precursors as well as more initial state cells are based on distinct analyses, and not contradictory.

4. The findings that CP region CTBs express more EMT markers is surprising as the general concept is EMT markers are induced in EVT precursors and not in STB-precursors. Authors should further verify that CP regions have more enrichment of EMT markers and BP region have enrichment for hypoxia and mTOR signaling.

<u>Response</u>: There are numerous novel findings in our single cell analysis, not all of which can be verified in one manuscript. It is already well-known that BP regions are enriched for hypoxia genes (PMID: 28167044 ; 31871275, 30042384). In order to focus the manuscript on "initial state" CTB and comparison to in vitro-derived TSC, we have removed discussion of these pathways from the manuscript (but left in the data in Table S11).

5. The data regarding TSCs is extremely confusing and a major concern. The original paper from Okae et al (supplementary data) clearly showed that the established TSC lines) from CTBs highly express genes such as BCAM, HMMR, TROAP (reported in original paper's RNA-seq data). Thus, the claim from the authors seems biased only based on their own TSC lines. It is absolutely important to culture TSCs properly (as gene expression pattern varies if they are overgrown, passaged more number of times and varies from one line to other). Authors should extensively characterize the TSC lines (chromosome 19 micro RNA expression and extent of other stem markers). Also, there is no (phase contrast/bright-field) images of colonies, which should be included. It is also recommended to perform bulk RNA-seq in those lines to better characterize gene expression program as the scRNA-seq data could be biased upon supervised clustering of marker genes.

Response: Our lab has extensive experience with derivation, culture, and differentiation of TSC lines (PMID: 35594858, 34454392, 34540826, 37787612), some of which have been characterized by bulk RNA-seq (PMID: 35594858, 34454392, 34540826). We routinely culture the cells in a sub-confluent fashion and apply strict criteria for determination of "healthy" TSC, which include surface expression of EGFR and ITGA6 at >90% and HLAG <10% (as noted in Figure 4h). We have now also included phase contrast/bright-field images of TSC colonies in Figure S8a. It is also important to point out that the original Okae et al. manuscript did not discuss BCAM, HMMR, or TROAP specifically; in fact, these three genes only appear within Supplementary Table 1, where expression of all ~24,000 human genes are noted. BCAM has since been highlighted as a "primitive CTB" marker (Shannon et al. Development, 2022; PMID: 35020896); however, this gene is widely expressed in all CTB clusters as well as in TSC. In fact, our data suggest that these (and other "initial state") markers are not entirely lacking in TSC; rather, they are simply less enriched (Figure 4d and Figure S9b).

6. The studies on spontaneous differentiation in 2% vs. 21% oxygen are problematic. The cells need better characterization and more extensive gene expression analyses. Authors should show TSC colony images, expression of various markers in those colonies and more extensive gene expression analyses (RNA-seq) to support their claim. It is important as authors themselves found deviation in gene expression programs (ITGA1). Further, the recent concept in the field that terminal EVT differentiation is inefficient in 2% oxygen. It is also important to note whether the Hypoxia effect is similar in acute (One passage in 2% oxygen after initial culture in 21%) vs. adapted (multiple passages in 2% oxygen) conditions.

Response: The studies comparing culture in 2% vs. 21% oxygen are based on our previously-published study (Wakeland et al. Am J Pathol. 2017; PMID: 28167044), as noted in the manuscript. In that study, we cultured CTB, directly following isolation from first trimester placenta, in DMEM/10% FBS, and found that culture in 21% oxygen caused spontaneous differentiation toward STB, while culture in 2% oxygen caused spontaneous differentiation toward EVT, based on a combination of marker expression and functional studies. In the current manuscript, we wanted to compare TSC to first trimester CTB, the cells from which they originate; thus, we applied the same culture conditions (as well as the same assays) to TSC in this manuscript. We have now added qPCR of additional STB and EVT markers (Figure S10c). Our findings, based on these limited assays (qPCR and immunostaining) suggest that TSC in fact behave differently: that while, overall, STB markers (CGA and CGB) are higher at 21% oxygen and EVT markers (ASCL2 and HLAG) at 2% oxygen, other markers are discrepant (EVT marker ITGA1 is increased in 21% oxygen). It is in fact possible, as the reviewer states (and the recent conference presentations from Dr. Beristain's lab regarding the role of high oxygen tension in EVT maturation, indicate), that TSC, being more similar to EVT precursors, in fact increase mature EVT markers (such as ITGA1) under 21% oxygen. Regardless, our data are consistent with the hypothesis (presented in our manuscript) that TSC are distinct from first trimester CTB, in that they lack the potential to spontaneously differentiate into bona fide STB in 2D.

7. Authors should characterize ITGA2 expression more carefully using in situ hybridization. The IF data is reliant on antibody efficiency.

<u>Response</u>: This was already included in the manuscript (see Supp Fig S9e).

8. How did authors ensure that all the ITGA5 (+) cells (in Fig. 5e and supplementary Fig. S11e) are CTBs? MACS shorting always have contamination from other cells (Fig. 5e shows many cells without ITGA5 expression and the cell morphology in panel 5e varies with panel S11e). It is necessary to test expression of non-trophoblast cell (such as endothelial or stromal cells) markers to eliminate possibility of contamination and more extensive marker analyses (RNA-seq) is necessary to support claim.

<u>Response</u>: We first isolated CTB using Percoll gradient centrifugation, then subjected these further to MACS using an antibody against ITGA5 (see Methods). We know based on flow cytometry for EGFR and HLAG that >99% of these cells are trophoblast. To clarify what was done for each experiment, we have now added a cartoon illustration of the methods for each figure.

9. The change in gene expression from CTB-TSC transition is not surprising (like ICM to ES cell transition) but interesting. However, it is only descriptive and authors should test which component in TSC medium induces this EVT-biased gene expression program

<u>Response</u>: Thank you for this suggestion, but this is beyond the scope of the current manuscript.

10. The ITGA5 expression in hypoxia is interesting. Does low O_2 actively bias CTB progenitors toward EVT-like identity via ITGA5? Does TSC culture condition induces ITGA5 in ITGA5(-) CTBs (if they are plated with TSC medium with or without hypoxia). How does Hypoxia drive EVT-like fate? Is this dependent on HIF-ITGA5 axis? Authors should treat cells with hypoxia inhibitors (HIF1α inhibitor) and test the cell fate of CTB Progenitors and ITGA5 expression.

<u>Response</u>: We have previously shown that low oxygen (2%) biases CTB toward EVT identity and that this requires hypoxia-inducible factor (HIF) signaling (see ref# 31, Wakeland et al. Am J Pathol. 2017; PMID: 28167044). TSC culture condition selects for an ITGA5+ identity, as shown in Fig. 5b-c; however, it is unclear whether it can convert ITGA5- cells to ITGA5+ ones, as we were unable to culture the ITGA5- cells in 2D under any condition.

Reviewer #2 (Remarks to the Author):

1 The authors have analysed human placental samples obtained in the mid and late first trimester and cells isolated for different regions of the early placenta to characterise the stem cell population. Of particular interest is the identification of the small subset of cells that are the progenitors of the cells that can be propagated as trophoblast stem cells (TSCs) in vitro. This is important work as these cells (originally described by Okae et, citation #7) have been very widely adopted by many labs in the field. The delineation of similarities and differences between these cells to their in vivo counterparts would be of considerable value to the field. Hence, I am enthusiastic about the work described.

<u>Response</u>: We appreciate and share the reviewer's enthusiasm!

2 The authors state that that the villous cytotrophoblast derived trophoblast stem cells (sometimes referred to as "Okae TSCs") "……. lack of spontaneous differentiation into STB" (line 72). This is a recurring theme and in line 463 – in the context of "spontaneous differentiation" the authors state that when they culture TSCs in DMEM/F12+FBS they spontaneously differentiate down the EVT lineage. However, this

is not “spontaneous” as it requires the withdrawal of the factors present in the stem-cell media that promote or potentiate WNT activation. This stated lack of spontaneous syncytialization is not an accurate reflection of the literature. For example, Shannon et al (PMID38359834 cited here #18) state, “In both hTSC-derived organoids (TB S-Org) and freshly isolated primary trophoblast-derived organoids (TB P-Org), progenitor trophoblasts spontaneously fuse to generate multinucleated hormone-producing syncytiotrophoblast (SCT).” Thus the wording in the manuscript needs revision. If the authors wish to argue that the process or readiness of TSC differentiation into STB is aberrant that that is fine but given what Sannon et al report the wording in the current manuscript. If the authors are referring to differentiation in 2-D culture then they need to specify this more precisely.

<u>Response</u>: With the exception of Figure 5h, our manuscript is focused **entirely** on comparison of TSC and primary CTB, ***cultured in 2D***; this is now further emphasized throughout the manuscript, including in the title. With regard to spontaneous differentiation: As an analogy, mouse TSC, upon removal of the maintenance factors FGF4 and MEF-conditioned media (or Activin A), spontaneously differentiate into both labyrinthine and junctional zone trophoblast (Tanaka et al. Science, 1998, PMID: 9851926; Parast et al. PLoS One, 2009, PMID: 19956639). Similarly, primary CTB, isolated from first trimester placenta, and cultured in DMEM/F12 + 10%FBS, spontaneously differentiate into either STB (under 21% oxygen) or EVT (under 2% oxygen) (Wakeland et al. Am J Pathol. 2017, PMID: 28167044). However, upon withdrawal of stem cell factors, and culture in DMEM/F12, human TSC spontaneously differentiate into EVT, but not bona fide STB (cells co-express EVT marker ITGA1 in normoxia, Figure 4i). In fact, unlike primary CTB, TSC require forskolin treatment (induction of PKA pathway) to fuse in 2D—another point of distinction between TSC and their cells of origin.

3 Line 134: What is the justification for claiming that clade/groups are “…….were transcriptionally similar”. Cells that a segregated into the separate clusters on the UMAP contain sufficiently different RNA populations to be separable. Hence, they are not “similar” to argue that a group of clusters are more similar to another group of clusters requires more robust justification.

<u>Response</u>: Hierarchical clustering first calculates the dissimilarity/Euclidean distance between data points (in our case UMAP clusters of cells) and then merges the two closest clusters, and repeats this until there’s only one cluster remaining, creating a dendrogram where the height of the branches indicates the distance at which the clusters were merged or split. This is a built-in command in Scanpy because it is a common analysis practice. Figure S2D shows that certain UMAP clusters are transcriptionally more similar to each other. Likewise, the dendrogram also shows that groups of UMAP clusters are more similar to some UMAP cluster groups than others, based on the calculated Euclidean distance. Our hierarchical clustering shows that, within the whole placenta dataset (Figure 1), CTB clusters 1, 6, and 10 were transcriptionally most similar to each other and were therefore grouped together (CTB group 1) and that this group was most similar to CTB group 2, containing CTB clusters 2, 4 and 8. To make our analyses clearer to the reader, we have changed the naming scheme from Clade to hierarchical clustering group throughout the manuscript.

4 Describing a group of clusters as a “clade” is not helpful. “Clade” implies common ancestry which is meaningless in the context of cells that are all derived from the same zygote. I appreciate that this could be useful short hand but if this is to be used then some justification and caveats need to be provided.

<u>Response</u>: We are sorry for the confusion and are not implying common ancestry, simply using “clade” to refer to a group of clusters. As noted above, we have now changed the naming scheme from Clade to hierarchical clustering group.

5a. Line 208-212. The conclusions drawn about the basal and chorionic plate genes requires more explanation. The genes of interest are apparently upregulated in EVT or pre-STB clusters. However, it is

not clear what "up regulated" means – the number of a cells in each cluster varies so is the increase just due there being more cells, or a more homogeneous cluster?

<u>Response</u>: We have added statistical analysis to this claim as well as changed the wording. This line now reads: "We found that the BP-CTB genes were significantly more highly expressed in the EVT clusters (Wilcoxon rank-sum p-value < 0.001), and the CP-CTB genes were significantly more highly expressed (Wilcoxon rank-sum p-value < 0.001) in our pre-STB clusters (**Fig. S4d**)."

5b. The term "up-regulated" also implies more than the authors intend – it implies a detected increase – ie something was low and then when measured later it was higher. However, what the authors actually mean is that something was simply higher in one cell (or cluster) than another when measured at the same time point.

<u>Response</u>: We have replaced the word "upregulated" with "enriched" throughout the manuscript to more accurately indicate our meaning.

6. My major reservation about the data presented is due to the low number of placental samples analysed. In the core experiments n=2 although for some experiments it is slightly higher. This is problematic although I fully understand the challenge of analysing more samples – tissue availability and the time and cost of more single cell RNAseq are not insignificant. However, the authors are drawing firm conclusion that there is more of one cell type at a given gestational age (6-8 weeks vs 12-14 weeks) or location in the placenta (basal vs chorionic plate). There will be sample to sample variation and heterogeneity within a single placenta. When one only has 2 samples it is not possible to make strong statements about any observed differences. This is a major weakness of the manuscript.

<u>Response</u>: We understand the reviewer's reservations with our sample sizes. In our whole placenta analysis, we performed single cell RNA-seq on two samples from early first trimester placenta and two from late first trimester placenta, then used two additional placentas for our basal and chorionic plate analysis; however, for the basal and chorionic plate analysis, we also performed spatial transcriptomics on an additional three samples. Finally, we also performed scRNA-seq on three different TSC lines to compare to our primary placenta samples, then applied scRNA-seq analysis to four different timepoints of TSC derivation from two separate early first trimester placentas. Nevertheless, we discussed these sample size constraints in the limitations section of our discussion.

7. Lines 184-5: The histological evaluation of the proposed progenitor cells – statements such as "more numerous", or "more enriched" (or better, simply "enriched") require some numerical assessment. This will not be robust when only 2 samples are available at each gestational age.

<u>Response</u>: The histological evaluation in lines 184-185 was done in five samples from both the basal and chorionic group. To make this clearer to the reader, we have added this to the caption for Figure 2D.

8. Lines 324-7: The authors describe experiments performed at 21% and 2% oxygen and describe these as "normoxia" and "hypoxia" respectively. The authors need to be careful with these terms – the first trimester placenta in vivo is in a physiologically low oxygen environment – it's low but normal so this is "normoxia" (explicitly not hypoxic although some of the published literature does use this term). However, the in vitro work described in this manuscript is performed at ambient oxygen. The text needs amendment to clarify - it's likely simplest not to refer to normoxia or hypoxia in the manuscript as the terms will likely cause confusion.

Response: We have now changed these terms, from normoxia and hypoxia, to 21% oxygen and 2% oxygen, respectively.

9. Line 135 (and elsewhere). The level of RNAs in a cell and hence those that distinguish different clusters depends on transcription and this is different in different cells (and clusters). However, the scRNAseq evaluates the level and a fixed point in time so is not a measure of transcription so it is inappropriate to say that cells are "were transcriptionally similar". Clearly transcription is a critical regulatory process but is not assessed directly by scRNAseq.

Response: We respectfully submit that the terminology used in our manuscript is broadly used by other groups when describing scRNA-seq data. However, to make the manuscript as accurate as possible we have eliminated the use of the phrase "transcriptionally similar" where possible.

Minor points
Fig 1 b please use a single continuous colour scale to shoe expression level

Response: We respectfully submit that the two-color sequential color maps allow the reader to better visualize the expression levels.

Fig 2d is much much too small

Response: This figure shows both a low-power image (in order to capture both the chorionic and basal plate for comparison in the same image) as well as a high-power inset image (in order to better capture the signal in distinct cell types). We have added further annotation to the figure in order to clarify the different structures.

Line 227 and elsewhere: chorionic **plate** cells

Response: This has now been updated.

Reviewer #3 (Remarks to the Author):

This is a descriptive single cell RNA-seq paper. The authors profile the transcriptomes of placentas from 2 early (6-8 weeks) and 2 late (12-14 weeks) first trimester, with early placentas further separated into basal and chorionic fractions. They also measure the transcriptomes of TSC cultured in vitro in 2D. Based on these data, the authors perform comparisons between early and late placentas, between chorionic and basal plate, as well as between in vivo trophoblast cells and TSC.

Response: We in fact profile a total of six first trimester placentas by single cell RNA-seq: 2 early (6-8 week) and 2 late (12-14 week) first trimester placentas are profiled at the whole placenta level (Figure 1), while two additional early (6-8 week) first trimester placentas are used to isolate CTB from chorionic and basal plates, then subjected to single cell analysis (Figure 3). Three additional early (6-8 week) first trimester placentas were used for spatial transcriptomics (Supp Fig 4). We also profiled 3 previously-derived TSC lines, as well as two additional TSC lines during the process of derivation from early (6-8 week) first trimester placentas by single cell RNA-seq. Other than simply comparing in vitro TSC, cultured in 2D, to their cells-of-origin by computational methods, we also culture the cells, in both 2D and 3D, in order to further confirm the differences between TSC and CTB. For comparison, Arutyunyan et al.'s 2023 Nature paper (PMID: 36991123) was based on analysis of essentially ONE early gestation placenta sample, while Shannon et al.'s Dev Cell paper (PMID: 38359834) used samples from only 3 patients, with additional comparison to previously-published data.

Although they found differences from these comparisons and provided another resource for the field, it is not clear what is new and different from other similar studies. In particular, in Shannon Dev Cell 2024 and Arutyunyan Nature 2023, similar comparisons are performed and these papers have also looked at primary trophoblast organoids and TSC in 3D. Moreover, both the rationale for the studies performed and summaries of the findings need to be more succinctly and clearly explained. The paper is dense and difficult to read. One focus is on the identity of the trophoblast progenitor(s) but this is not resolved nor where they reside.

Response: As described above, our sample numbers exceeded those published by Arutyunyan et al. and Shannon et al. In addition, as now even further emphasized in the revised manuscript, this is the first study to: 1) compare chorionic vs. basal plate CTB, both by single cell RNA-seq and spatial transcriptomics; and 2) profile TSC, ***cultured in 2D***, by single cell RNA-seq, and compare them to their cells-of-origin. We have now clarified the methods performed in each figure using a cartoon summary, and have re-focused the manuscript on the two above, novel points.

Major concerns:
1. There are many 'clusters' and 'clades' mentioned in Figs. 1, 3, 4. The naming of these clusters (e.g., CTB1, CTB2) is confusing. This makes each cluster unclear and difficult to compare across different figures (for example, between Fig. 1d and Fig. 3a). How do the CTB subclusters align across the three figures (Fig. 1d, Fig. 3a, and Fig. 4a)? Consistent terminology and clearer alignment are needed. In addition, it is unclear how these clusters correspond to previously reported trophoblast subtypes, and what is new and different from previous characterizations.

Response: We have changed the term clades to "groups" and changed the CTB group names for the BP and CP scRNA-seq to CTB groups A and B, so as to distinguish them from the whole placenta CTB groups 1-3. Our CTB naming scheme was chosen in order to assign cells in an unbiased way, allow for the initial state cells to be computationally assigned, to find differences and similarities between early and late first trimester CTB, and to determine the similarity and differences between our TSC and initial state CTB. We scored the top 200 genes from each of our CTB clusters with the data published in Vento-Tormo et al's. 2018 Nature article (PMID: 30429548) as well with Arutyunyan et al's. 2023 Nature article (PMID: 36991123). We have shown the results in UMAPs below but decided not to include this data in the manuscript because we do not feel these comparisons add additional insights into these groups.
The Vento-Tormo et al. data are focused less on trophoblast cell types and therefore lack granularity for further assignment of our CTB subclusters. The Arutyunyan et al data is more focused on trophoblast cell types but comes primarily from a single placenta that is 9 weeks gestational age (GA), which is abnormally attached to the underlying uterus (e.g. part of a gravid hysterectomy for first trimester placenta accreta spectrum disorder). Our CTB group 3 cluster, which contains a high percentage of our late first trimester placenta and is therefore arguably most similar in GA to their data, not surprisingly corresponds to their VCT cluster. Our CTB group 1 cluster, the cluster that contains our initial state CTB and a high percentage of our early first trimester placenta cells, generally corresponds to their proliferating VCT cluster which they mention contains, "...known stem and progenitor cell markers". Of note, in their trajectory analysis, they manually set their initial state as their VCT cluster instead of computationally assigning it as we have done here. Our CTB group 2 cluster is split between several VCT clusters and although does not contain markers for non-trophoblast cells, EVT, or STB, it has lower expression of many canonical CTB markers possibly because it contains a high percentage of early first trimester placenta cells.

Vento-Tormo et al:

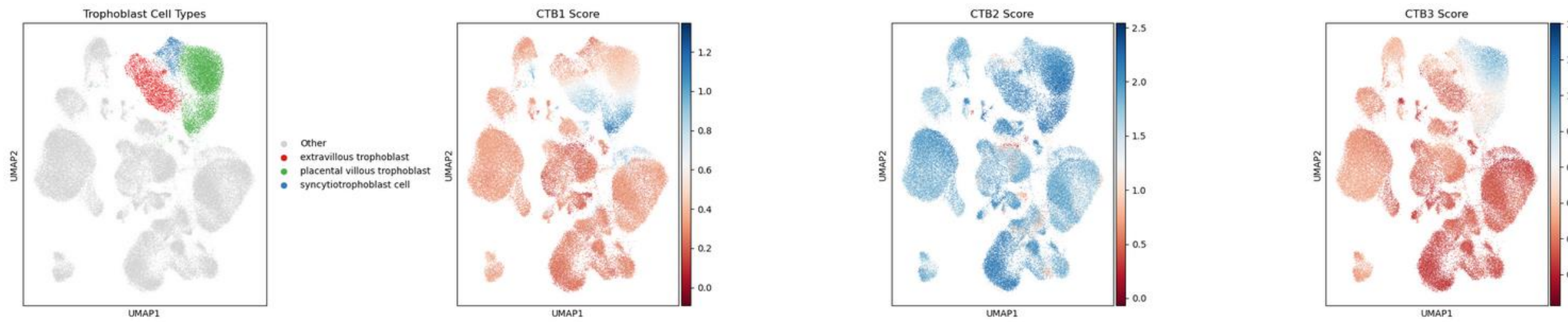

Arutyunyan et al:

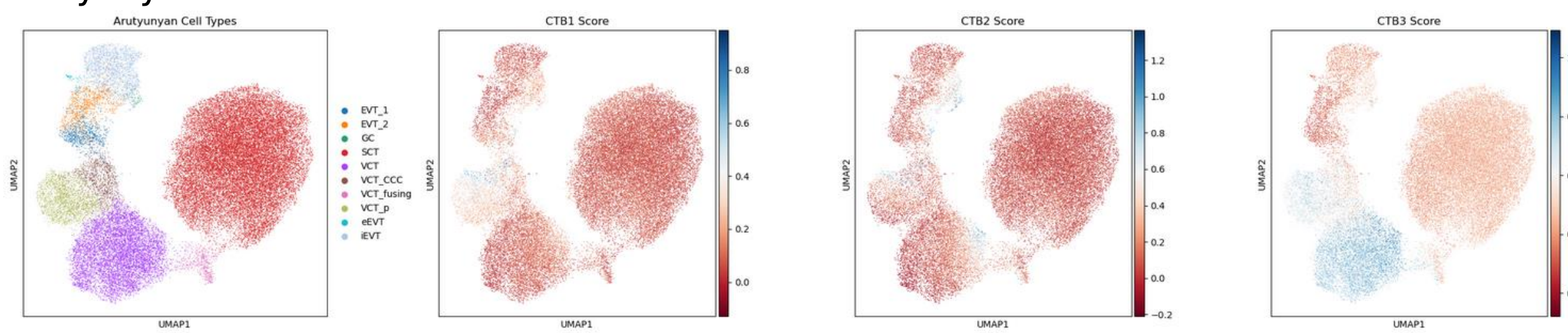

2. There are other public databases that have scRNA-seq data from placentas throughout the first trimester. Although this data has not been studied to show gestational-age changes, it should be analysed to incorporate into the data here. This would also strengthen the findings as only 2 samples from early or late gestation have been used here.

<u>Response</u>: We have used the meta analysis of multiple different scRNA-seq papers, performed by Derisoud et al. (PMID: 38478759), in order to annotate our clusters in this manuscript, and have now further clarified the annotation of different clusters/clades, applying uniform nomenclature, across our figures.

3. The authors identified a CTB initial state expressing HMMR, TROAP, CENPA and MXD3. HMMR and CENPA are G2M phase genes. Are they defining CTB initial states or just proliferating cells? How do these cells compare to trophoblast subtypes described in previous studies? Do they correspond to proliferative CTBs previously reported? Additionally, the expression of HMMR and TROAP appears weak in the initial state (Fig. 2c).

<u>Response</u>: Please see our response to Reviewer 1's comment #2 above as well as our response to your first comment. To expand our response to comment #1, Arutyunyan et al. defined VCT and VCT_p clusters using the following markers: TP63, CDH1, LRG5, L1TD1, WLS, TNIK, SEMA3F, NRP2, and BCAM. Many of these markers, such as BCAM, are found in not only their VCT and VCT_p clusters but also in their CTB column cells (VCT_CCC) and their SCT precursors, VCT_fusing. In fact, in their methods, they write that they combine the VCT and VCT-p clusters when they do their differential expression analysis and manually select their VCT cluster as their initial state when performing their trajectory analysis. Instead of manually selecting a cluster as our initial state, we wanted to computationally define our initial state cells in the most unbiased way possible and not limit ourselves to a set of predefined cells heavily reliant on a selected cluster resolution setting. We therefore used CellRank's Generalized Perron Cluster Cluster analysis (GPCCA) with the VelocityKernel to compute a transition matrix based on RNA velocity and identify macrostates of cellular dynamics, including the initial state. After computationally defining a small set of initial state cells, we asked what genes were differentially expressed in these cells compared to all of our other trophoblast cells. HMMR and TROAP were two novel genes that were, not necessarily most highly expressed, but most specifically expressed in our initial state cells compared to our other trophoblast cells. Below, we have compared our initial state cells to both Vento-Tormo et al's. data (PMID: 30429548) and Arutyunyan et al's. data (PMID:

36991123). Our initial state seems to fall within Vento-Tormo's CTB and within Arutyunyan's VCT_p cluster, which as mentioned in our response to comment #1, contains "...known stem and progenitor cell markers".

Vento Tormo et al:

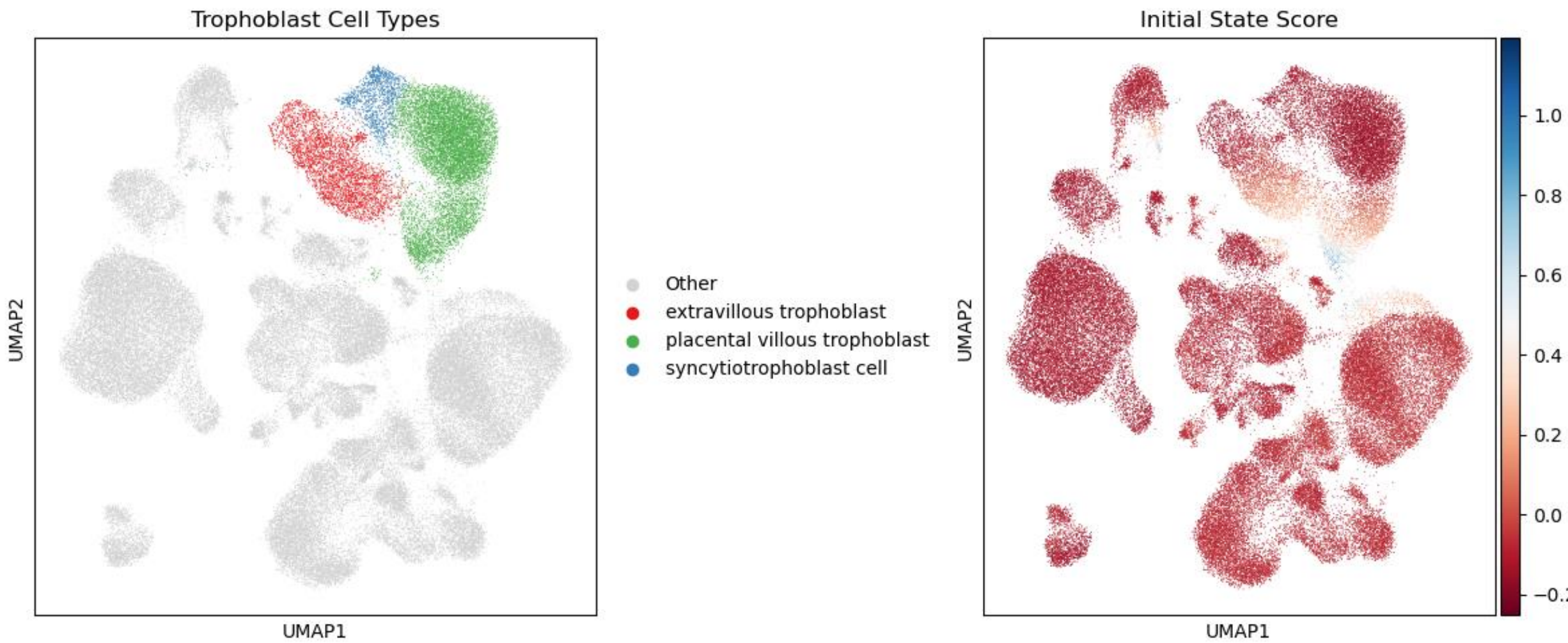

Arutyunyan et al:

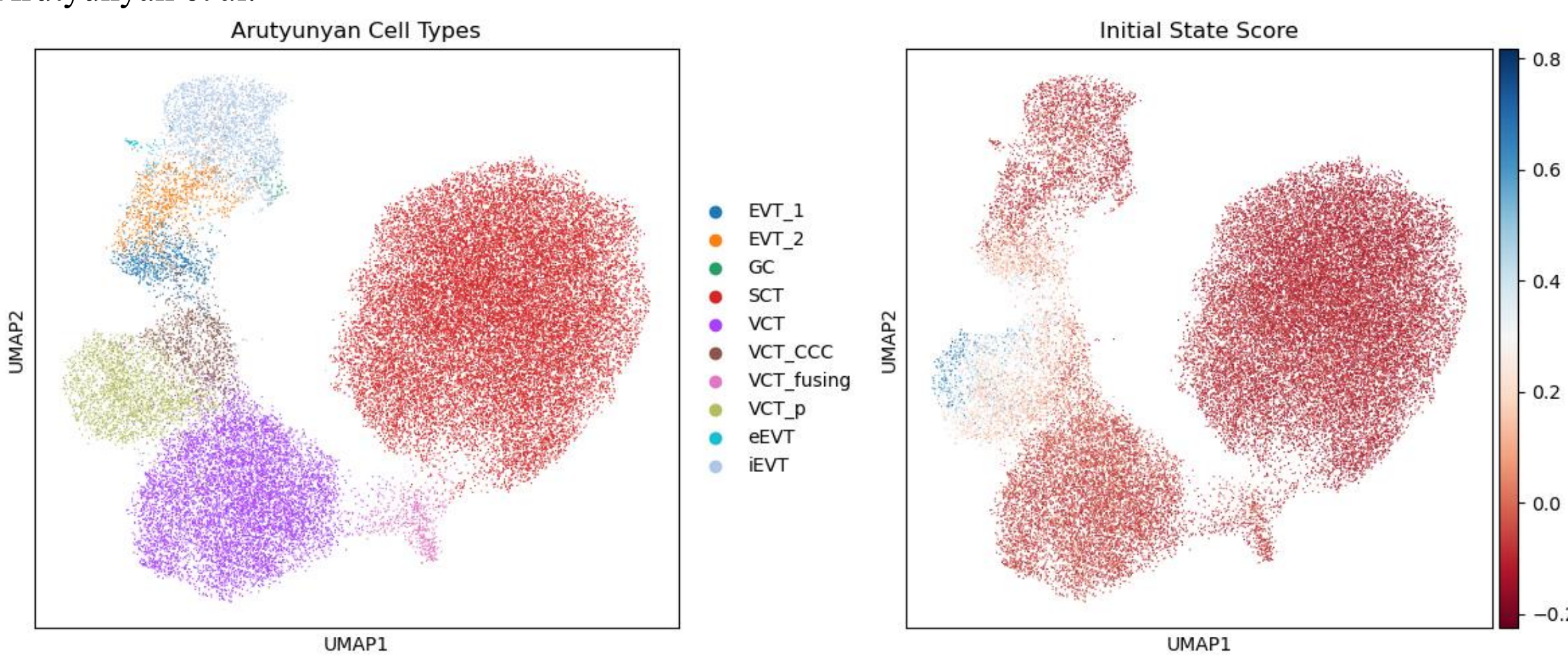

4. Marsh et al (PMID: 35796428) (not cited) also performed scRNA-seq on the two regions (villous and smooth chorion) in the second trimester. How do the findings in this study compare to those from the second trimester?

<u>Response</u>: As mentioned in the manuscript, and above, the goal of this study was to understand the origin of TSC, which were first derived from early first trimester CTB. Comparison to second trimester CTB, including to villous and smooth chorion (fetal membranes) is beyond the scope of this manuscript.

5. The section comparing TSC is not novel and is poorly performed. Okae did not separate cells based on ITGA6+ expression. Firstly, the flow cytometry needs to show the scatter plot, gating strategy, live/dead marker and negative control and then representative histograms of all markers. The error bars seem very small for 6 lines as well? It is now well established that TSC have a high proportion of ITGA2+ cells compared with trophoblast from normal placentas. The data on ITGA2, ITGA5 and ITGA6 expression (including IHC and ISH) has also all been described previously. Culturing primary trophoblast and

analysing integrin expression is also not novel and the culture in different oxygen levels introduces another variable.

<u>Response</u>: As mentioned in the manuscript, and above, this is the first manuscript to subject TSC (cultured in 2D) to detailed profiling, including by single cell analysis. Our comparison is focused on the comparison between TSC and CTB, and in fact shows a clear distinction between the in vitro-derived cells and "initial state" CTB. According to the methods section in Okae et al.'s 2018 Cell Stem Cell article (PMID: 29249463), they did indeed derive TSC from ITGA6+ CTB. Table S3 shows that all of their TS^{CT}, were derived from (6 to 9 week) CT cells and their methods section indicates that, "CT cells were immunomagnetically purified using the EasySep PE selection kit and a PE-conjugated anti-ITGA6 antibody." Other groups have indeed shown TSC to be enriched in ITGA2; however, our manuscript is the first to show the quick gain of proximal column trophoblast markers, ITGA2 and ITGA5, during the derivation process, again firmly validating the selection of a relatively rare in vivo cell type when transitioning from primary CTB to TSC. The IHC and ISH for these markers, particularly the dual ISH for ITGA2 and ITGA5 (Fig S9e), is shown here to emphasize the rarity of such dual-positive cells in vivo. Finally, as stated in response to reviewer 1's comment #6 above, the culture in different oxygen levels follows our previous publication (Wakeland et al. Am J Pathol. 2017, PMID: 28167044) and is done here to, again, compare primary CTB to TSC—going beyond computational analysis and further validating the phenotypic differences between these cell states.
Regarding the flow cytometer analysis, cells were always gated to eliminate dead cells and cell debris. These TSCs are well-established lines and therefore, the expression of EGFR, ITGA6, ITGA5, ITGA2, HLA-G, and ITGA1 is pretty consistent across all six lines and therefore, the error bars are very small.

6. The authors show that there are 3 clades of CTB. Why are the three clades not clustered together in Fig. 1c? Moreover, clade 2 (CTB2, 4, 8) has very low expression of CTB markers. The authors need to check whether this clade represents a truly distinct subpopulation of CTB. It is quite possible that they are low-quality cells with a much lower number of genes detected (usually accompanied by a higher percentage of mitochondrial genes), which would separate them from other clusters in the UMAP. This issue is also present in several other figures in the paper, including CTB2, 6 in Fig. 3a, and CTB3, 5 in Fig. 4a.

<u>Response</u>: Please see methods, which clearly delineate QC for single cell analysis, excluding low-quality cells due to high percentage of mitochondrial genes. Doublet scores and mitochondrial expression was again checked to ensure that these cells are of high quality. Clade 2 (now named CTB group 2), consisting of CTB clusters 2/4/8 in the whole placenta, has low levels of some CTB markers (such as EGFR) but high levels of other, rather specific, markers of CTB, including VGLL1. As noted in our response to comment #1, we believe that these cells may represent CTB present in early first trimester placenta. We are currently evaluating these cells further; however, this signature is noted in some of the trophoblast cells in Vento-Tormo's data, as seen below:

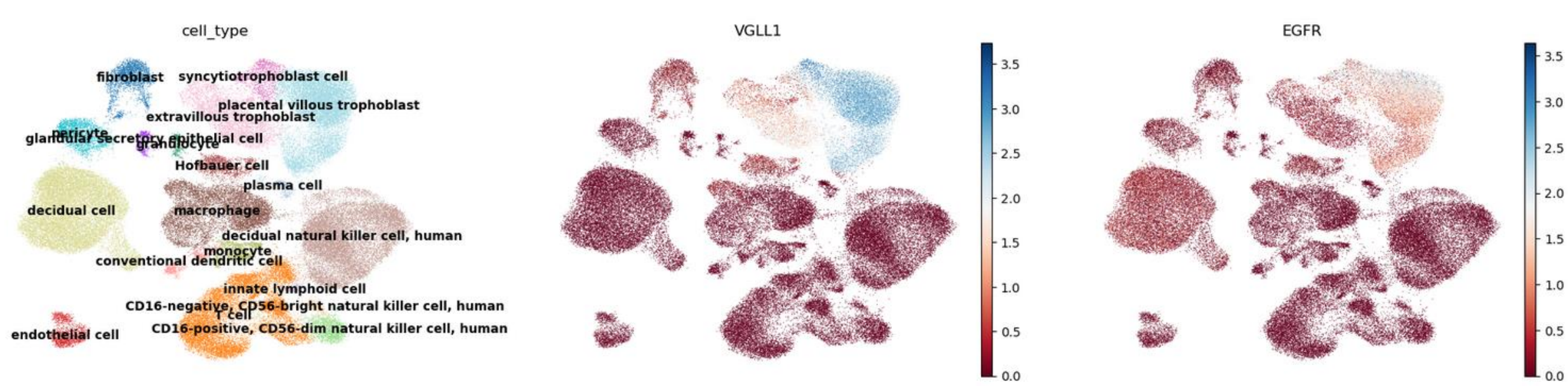

7. Are the trophoblast cells shown in Fig. 1d from chorionic or basal plate or both?

Response: Figure 1 is looking at data from the whole placenta (early vs. late first trimester), from which trophoblast were computationally separated and compared across these two different gestational timepoints. We have now clarified the methods used in each figure, using a cartoon illustration at the top of each figure.

8. Cluster 21 in Fig. 1a is likely to be doublets as it expresses VIM (which is absent in trophoblast cells).

Response: We agreed with you and in fact removed this cluster from further analysis, as clearly stated in the results. We wrote: "We were interested exclusively in the trophoblast clusters (0, 2, 3, 4, 5, 8, 10, 11, 13, 14, 17, 19) and removed all non-trophoblast clusters (Fig. S2a)." This cluster was never part of our trophoblast analyses.

9. The 'non-trophoblast cells' have been defined with markers for Hofbauer cells, fibroblasts and endothelial cells. However, in these surgical samples there is always contamination from maternal blood and decidual cells. Additional markers are needed to encompass all leukocytes (CD45, CD3, CD19, CD56) and glandular epithelial cells.

Response: We in fact used HLA-A and -B as markers of non-trophoblast, which should apply to both fetal as well as maternal cells. We have also looked at the markers mentioned above and they did not identify additional non-trophoblast cells.

10. Lines 185-186: the authors concluded that HMMR positive cells "are more numerous in early first trimester placentas, and significantly more enriched at the basal, compared to chorionic plate" based on Fig. 2d. This needs quantification.

Response: This is clearly visible from the ISH images in Figure 2d, where more brown signal is present in the 6 week sample and near the basal plate; this figure has now been further annotated to clarify the structures.

11. Lines 209-210 and Fig. S4c: it is unclear that CP-CTB genes were upregulated in pre-STB.

Response: We have changed Figure S4C (to violin plots) and clarified the description in the results to make this point more clear.

12. Line 218: given that ASCL2 is an EVT marker gene, why did the authors use ASCL2 to define BP cells?

Response: ASCL2 is also expressed in villous CTB of anchoring villi (see Figure 2b in Wakeland et al. Am J Pathol. 2017; PMID: 28167044).

13. Fig. 3b: It is unclear how the size factor was calculated, and no details are provided in the Methods section.

Response: The size factor calculation details have now been added to the methods section.

14. Fig. 5b: why are the cells separated by days? It is unclear what cell types are present.

Response: This figure is showing the TSC derivation process, which involves culture of primary CTB, isolated from 6-8 week gestation placenta by Percoll gradient centrifugation, in TSC media in 2D over 10 days in culture.

15. The colour scheme used is confusing (red indicates low expression and blue indicates high expression).

<u>Response</u>: The color scheme is python's colormap, cmap=RdBu, a standard diverging colormap designed to highlight deviations from a central neutral value. We have placed legends alongside the figures to help readers. This color scheme is common due to the fact that red light has lower energy and frequency and blue light has higher energy and frequency and can also be seen in weather maps where low pressure is depicted with red and high pressure with blue.

16. There is a paper comparing the trophoblast from basal and chorionic villi, mainly focused on the derived organoids, but they also do scRNAseq on the tissue (https://doi.org/10.1101/2024.11.19.624345) on BioRxiv.

<u>Response</u>: This manuscript is looking at smooth chorion and chorionic villi from term placenta—data which are worth comparison to the Marsh et al. study, but again, irrelevant to the goal of our study (comparison of TSC to their early first trimester cells-of-origin).

17. In Fig.1d it looks like both chorionic and basal plate are present, but the panels are really too small (maybe also a dotted line separating the two regions would help) and how they then claim the cells are more numerous in early compared to late is not clear.

<u>Response</u>: The reviewer is likely referring to Figure 2D; as stated above, this figure has now been further annotated to clarify the findings.

18. What is the sex of the samples?

<u>Response</u>: We've now added this to the supplementary table.

Reviewers' comments:

Reviewer #1 (Remarks to the Author):

The manuscript by Robert Morey and Coauthors focused on single-cell genomics to identify heterogeneity in gene expression patterns in Cytotrophoblast (CTB) cells of the early gestation human placenta and human trophoblast stem cells. Authors made the following major findings (1) Gene expression program in CTBs varies with gestation, (2) CTB progenitors in basal plate have gene expression bias toward EVT precursors, and CTB progenitors in chorionic plate have gene expression bias toward STB precursors and (3) Human TSCs have gene expression pattern more similar to VCT-CCC (the proximal column EVT precursors). Authors also indicated that ITGA5 positive CTBs could be directly differentiated towards EVTs in 2D culture, whereas ITGA5(-) CTBs fail to do so. The manuscript suggest that distinct CTB states exist in different regions of the placenta as early as six weeks gestation and that established TSC lines most closely resemble ITGA5+ CTB, biased toward the EVT lineage. The study has reiterated findings from several prior studies and seems very descriptive. There are major gaps in the manuscript and some of the data is confusing. Following comments should help the manuscript.

1. *The manuscript at its present form is descriptive and many of the information are reiteration of earlier findings, such as different subtypes of CTBs exist is well known from other studies. It is also well known that CTBs differ in their gene expression patterns across gestation. Thus, the advancement to our knowledge is only incremental. More mechanistic questions, such as why early first-trimester (6 weeks) vs. late first trimester CTBs (12-13 weeks) differ significantly in gene expression programs (such as loss of mitotic genes) or how they differ in epigenetic programming could have generated more interest to the field.*

<u>Response</u>: This is the first study to compare, not just CTB populations between early and late first trimester, but also CTB in different regions of the placenta (chorionic vs. basal plate) at the single cell level, using both 10X and spatial transcriptomics. The impetus for this comparison was to compare TSC and their cells-of-origin, namely, CTB from early first trimester (<10 week gestation) placentas. Therefore, we set out to compare the CTB subpopulations at extremes of this gestational age range (6-8 weeks vs. 12-14 weeks) and identified a late first trimester CTB cell population that contained genes known to play regulatory roles in the placenta such as angiogenesis, immune regulation, structural integrity, and cellular differentiation as well as two early first trimester CTB populations that were enriched for pathways related to cell proliferation, differentiation, KRAS signaling, and immune response. Additionally, unlike previous studies that have focused on TSC cultured in 3D (as trophoblast organoids), this is the first study to compare TSC ***cultured in 2D*** to *in vivo* CTB at the single cell level; as such, we have changed the title to further reflect this distinguishing feature of our study.

2. *Authors indicated that a fraction of initial stage CTBs (more in 6 weeks and less in 13 weeks) express mitotic markers and they are mostly confined at the Basal Plate (BP) region. The whole claim is based on studies with only one gene, HMMR. How about looking at mitosis itself? They should perform additional analyses (like phosphohistone H3 and other cell cycle markers) to define heterogeneity of mitotically active/proliferating CTB populations. It is also surprising to see so less number of CTBs expressing HMMR around the chorionic plate (CP) region. Is the CP region CTBs not proliferating at 6 weeks? The possible causes of heterogeneity of distribution of initial stage CTBs should have been included in the discussion.*

<u>Response</u>: It is well-known that proliferative CTB decrease in number through gestation (Lee et al. Hum Pathol. 2007; PMID: 17397906) and are enriched at the basal plate (Lee et al. Development. 2018; PMID: 29540503). Although stem cells are by definition proliferative, our study was focused on identifying ***"initial state" CTB***, as a possible source of TSC, using computational methods, and not simply

mitotically-active CTB. Our analysis led to the identification of a CTB subcluster (n=30 cells); we then sought to identify the genes most specifically expressed in these 30 cells, compared to other primary CTB (Supp. Table 10) and our TSC (Supp. Table 14), and found that these cells were defined by a group of genes, including HMMR and TROAP. Moreover, we also show the expression of the transcription factors MXD3 and CENPA in this initial state cell population (Figure S4A). We have now further annotated the ISH figures for HMMR and TROAP (Supp Fig 8c), which are consistently enriched in early first trimester samples, and at the basal plate. It is also important to point out that HMMR and TROAP have roles beyond the cell cycle: they are also known to be involved in cell motility and cell transformation/metastasis (PMID: 1376732, 24367099, 21940500, 36750558, 39990663). HMMR is known to play a significant role in cancer stem cells, where it promotes self-renewal and differentiation through EMT and activating signaling pathways like TGFβ/Smad2 (PMID: 30802150, 24710409). Additionally, TROAP is also highly expressed in cancer stem cells and has been shown to promote not just proliferation but also migration and invasion (PMID: 34077623, 33500384). We now also mention the heterogeneity of distribution of these "initial state" CTB in the discussion.

3. *The region of interest for spatial transcriptomics for basal plate (BP) region is biased as authors mostly selected anchoring villi regions (Fig. S4B), which contain a lot of proximal column CTBs. Authors should also select regions beyond anchoring villi. Otherwise the results that the BP region contains more initial stage CTBs as well as it contains more EVT precursors are contradictory claims. The comparison of spatial transcriptomics with scRNA-seq is also biased as it only considers the selectively enriched genes.*

<u>Response</u>: Within a dilation & curettage (D&C) specimen, which describes all of our first trimester placenta samples, the only way to recognize the basal plate is based on morphology of the anchoring villi, in the same way that the recognition of the chorionic plate depended on identification of the sac. However, for spatial transcriptomics, we used EGFR immunostaining for segmentation: in other words, within the "regions of interest" or ROI, we selected only the EGFR+ cells in the anchoring villi, in order to profile the CTB, and not proximal column trophoblast, from these regions. Our spatial data revealed that there are differences between EGFR+ CTB in the BP and CP regions of the placenta, showing that genes enriched in the BP-CTB were more highly expressed in the EVT cluster (EVT9) of our whole placenta single cell data (early and late first trimester placentas; Figure S5d).

The fact that we found differences between BP and CP in our spatial data prompted us to perform scRNA-seq on CTB isolated from dissected CP (sac) and BP (anchoring villous tips) regions of two early first trimester placentas, using Percoll gradient centrifugation. We then used the initial state CTB gene expression profile from our combined early and late first trimester whole placenta dataset to locate cells with a similar gene expression profile in our BP/CP dataset, and found that the initial state signature genes were most highly expressed in the CTB1 cluster, which is enriched in basal plate cells (Figure 3c-d). Thus, the conclusion that the BP region contains both more EVT precursors as well as more initial state cells are based on distinct analyses, and not contradictory.

4. *The findings that CP region CTBs express more EMT markers is surprising as the general concept is EMT markers are induced in EVT precursors and not in STB-precursors. Authors should further verify that CP regions have more enrichment of EMT markers and BP region have enrichment for hypoxia and mTOR signaling.*

<u>Response</u>: There are numerous novel findings in our single cell analysis, not all of which can be verified in one manuscript. It is already well-known that BP regions are enriched for hypoxia genes (PMID: 28167044 ; 31871275, 30042384). In order to focus the manuscript on "initial state" CTB and comparison to in vitro-derived TSC, we have removed discussion of these pathways from the manuscript (but left in the data in Table S11).

5. *The data regarding TSCs is extremely confusing and a major concern. The original paper from Okae et al (supplementary data) clearly showed that the established TSC lines) from CTBs highly express genes such as BCAM, HMMR, TROAP (reported in original paper's RNA-seq data). Thus, the claim from the authors seems biased only based on their own TSC lines. It is absolutely important to culture TSCs properly (as gene expression pattern varies if they are overgrown, passaged more number of times and varies from one line to other). Authors should extensively characterize the TSC lines (chromosome 19 micro RNA expression and extent of other stem markers). Also, there is no (phase contrast/bright-field) images of colonies, which should be included. It is also recommended to perform bulk RNA-seq in those lines to better characterize gene expression program as the scRNA-seq data could be biased upon supervised clustering of marker genes*.

Response: Our lab has extensive experience with derivation, culture, and differentiation of TSC lines (PMID: 35594858, 34454392, 34540826, 37787612), some of which have been characterized by bulk RNA-seq (PMID: 35594858, 34454392, 34540826). We routinely culture the cells in a sub-confluent fashion and apply strict criteria for determination of "healthy" TSC, which include surface expression of EGFR and ITGA6 at >90% and HLAG <10% (as noted in Figure 4h). We have now also included phase contrast/bright-field images of TSC colonies in Figure S9a. To further address the reviewer's concern, we combined bulk RNA-seq data from the four original TSC lines published by Okae (2018) with our TSC and early first-trimester placental dataset to assess transcriptomic similarity between their TSCs and ours. Using correlation analysis, UMAP distance measurements, and a permutation-based test, we found that the published Okae TSC lines most closely resemble the TSC1 cluster, representing the majority of our TSC (see Figure 4b). These results are now provided in Supplementary Table S13.

Additionally, we would like to point out that the original Okae et al. manuscript did not discuss BCAM, HMMR, or TROAP specifically; in fact, these three genes only appear within their Supplementary Table 1, where expression of all ~24,000 human genes are noted. BCAM has since been highlighted as a "primitive CTB" marker (Shannon et al. Development, 2022; PMID: 35020896); however, this gene is widely expressed in all CTB clusters as well as in TSC. In fact, our data suggest that these (and other "initial state") markers are not entirely lacking in TSC; rather, they are simply less enriched (Figure 4d and Figure S10b).

6. *The studies on spontaneous differentiation in 2% vs. 21% oxygen are problematic. The cells need better characterization and more extensive gene expression analyses. Authors should show TSC colony images, expression of various markers in those colonies and more extensive gene expression analyses (RNA-seq) to support their claim. It is important as authors themselves found deviation in gene expression programs (ITGA1). Further, the recent concept in the field that terminal EVT differentiation is inefficient in 2% oxygen. It is also important to note whether the Hypoxia effect is similar in acute (One passage in 2% oxygen after initial culture in 21%) vs. adapted (multiple passages in 2% oxygen) conditions.*

Response: The studies comparing culture in 2% vs. 21% oxygen are based on our previously-published study (Wakeland et al. Am J Pathol. 2017; PMID: 28167044), as noted in the manuscript. In that study, we cultured CTB, directly following isolation from first trimester placenta, in DMEM/10% FBS, and found that culture in 21% oxygen caused spontaneous differentiation toward STB, while culture in 2% oxygen caused spontaneous differentiation toward EVT, based on a combination of marker expression and functional studies. In the current manuscript, we wanted to compare TSC to first trimester CTB, the cells from which they originate; thus, we applied the same culture conditions (as well as the same assays) to TSC in this manuscript. We have now added qPCR of additional STB and EVT markers (Figure S11c). Our findings, based on these limited assays (qPCR and immunostaining) suggest that TSC in fact behave differently: that while, overall, STB markers (CGA and CGB) are higher at 21% oxygen and EVT markers (ASCL2 and HLAG) at 2% oxygen, other markers are discrepant (EVT marker ITGA1 is

increased in 21% oxygen). It is in fact possible, as the reviewer states (and the recent conference presentations from Dr. Beristain's lab regarding the role of high oxygen tension in EVT maturation, indicate), that TSC, being more similar to EVT precursors, in fact increase mature EVT markers (such as ITGA1) under 21% oxygen. Regardless, our data are consistent with the hypothesis (presented in our manuscript) that TSC are distinct from first trimester CTB, in that they lack the potential to spontaneously differentiate into bona fide STB in 2D.

7. *Authors should characterize ITGA2 expression more carefully using in situ hybridization. The IF data is reliant on antibody efficiency*.

Response: This was already included in the manuscript (see Supp Fig. S10e).

8. *How did authors ensure that all the ITGA5 (+) cells (in Fig. 5e and supplementary Fig. S11e) are CTBs? MACS shorting always have contamination from other cells (Fig. 5e shows many cells without ITGA5 expression and the cell morphology in panel 5e varies with panel S11e). It is necessary to test expression of non-trophoblast cell (such as endothelial or stromal cells) markers to eliminate possibility of contamination and more extensive marker analyses (RNA-seq) is necessary to support claim*.

Response: We first isolated CTB using Percoll gradient centrifugation, then subjected these further to MACS using an antibody against ITGA5 (see Methods). We know based on flow cytometry for EGFR and HLAG that >99% of these cells are trophoblast. To clarify what was done for each experiment, we have now added a cartoon illustration of the methods for each figure.

9. *The change in gene expression from CTB-TSC transition is not surprising (like ICM to ES cell transition) but interesting. However, it is only descriptive and authors should test which component in TSC medium induces this EVT-biased gene expression program*

Response: Thank you for this thoughtful suggestion. However, systematically dissecting the contribution of each individual medium component to TSC derivation is beyond the scope of the current manuscript. Nevertheless, to further characterize changes that occur during the derivation process, we have added additional analyses characterizing gene expression dynamics across the ten-day transition from isolated CTB to TSC. Specifically, we performed differential expression analyses across each derivation timepoint (day 0, day 1, day 4, and day 10), and identified genes and transcription factors correlated with diffusion pseudotime throughout the derivation trajectory. We then conducted pathway enrichment analyses to define stage-specific functional programs enriched during this process. These new analyses are presented in Tables S15–S18 and Figure S12e.

10. *The ITGA5 expression in hypoxia is interesting. Does low O_2 actively bias CTB progenitors toward EVT-like identity via ITGA5? Does TSC culture condition induces ITGA5 in ITGA5(-) CTBs (if they are plated with TSC medium with or without hypoxia). How does Hypoxia drive EVT-like fate? Is this dependent on HIF-ITGA5 axis? Authors should treat cells with hypoxia inhibitors (HIF1α inhibitor) and test the cell fate of CTB Progenitors and ITGA5 expression*.

Response: We have previously shown that low oxygen (2%) biases CTB toward EVT identity and that this requires hypoxia-inducible factor (HIF) signaling (see ref# 31, Wakeland et al. Am J Pathol. 2017; PMID: 28167044). TSC culture condition selects for an ITGA5+ identity, as shown in Fig. 5b-c; however, it is unclear whether it can convert ITGA5- cells to ITGA5+ ones, as we were unable to culture the ITGA5- cells in 2D under any condition.

Reviewer #2 (Remarks to the Author):

1 *The authors have analyzed human placental samples obtained in the mid and late first trimester and cells isolated for different regions of the early placenta to characterise the stem cell population. Of particular interest is the identification of the small subset of cells that are the progenitors of the cells that can be propagated as trophoblast stem cells (TSCs) in vitro. This is important work as these cells (originally described by Okae et, citation #7) have been very widely adopted by many labs in the field. The delineation of similarities and differences between these cells to their in vivo counterparts would be of considerable value to the field. Hence, I am enthusiastic about the work described.*

__Response:__ We appreciate and share the reviewer's enthusiasm!

2 *The authors state that that the villous cytotrophoblast derived trophoblast stem cells (sometimes referred to as "Okae TSCs") "……. lack of spontaneous differentiation into STB" (line 72). This is a recurring theme and in line 463 – in the context of "spontaneous differentiation" the authors state that when they culture TSCs in DMEM/F12+FBS they spontaneously differentiate down the EVT lineage. However, this is not "spontaneous" as it requires the withdrawal of the factors present in the stem-cell media that promote or potentiate WNT activation. This stated lack of spontaneous syncytialization is not an accurate reflection of the literature. For example, Shannon et al (PMID38359834 cited here #18) state, "In both hTSC-derived organoids (TB S-Org) and freshly isolated primary trophoblast-derived organoids (TB P-Org), progenitor trophoblasts spontaneously fuse to generate multinucleated hormone-producing syncytiotrophoblast (SCT)." Thus the wording in the manuscript needs revision. If the authors wish to argue that the process or readiness of TSC differentiation into STB is aberrant that that is fine but given what Sannon et al report the wording in the current manuscript. If the authors are referring to differentiation in 2-D culture then they need to specify this more precisely.*

__Response:__ With the exception of Figure 5h, our manuscript is focused **entirely** on comparison of TSC and primary CTB, ***cultured in 2D***; this is now further emphasized throughout the manuscript, including in the title. With regard to spontaneous differentiation: As an analogy, mouse TSC, upon removal of the maintenance factors FGF4 and MEF-conditioned media (or Activin A), spontaneously differentiate into both labyrinthine and junctional zone trophoblast (Tanaka et al. Science, 1998, PMID: 9851926; Parast et al. PLoS One, 2009, PMID: 19956639). Similarly, primary CTB, isolated from first trimester placenta, and cultured in DMEM/F12 + 10%FBS, spontaneously differentiate into either STB (under 21% oxygen) or EVT (under 2% oxygen) (Wakeland et al. Am J Pathol. 2017, PMID: 28167044). However, upon withdrawal of stem cell factors, and culture in DMEM/F12, human TSC spontaneously differentiate into EVT, but not bona fide STB (cells co-express EVT marker ITGA1 in normoxia, Figure 4i). In fact, unlike primary CTB, TSC require forskolin treatment (induction of PKA pathway) to fuse in 2D—another point of distinction between TSC and their cells of origin.

3 *Line 134: What is the justification for claiming that clade/groups are "…….were transcriptionally similar". Cells that a segregated into the separate clusters on the UMAP contain sufficiently different RNA populations to be separable. Hence, they are not "similar" to argue that a group of clusters are more similar to another group of clusters requires more robust justification.*

__Response:__ Hierarchical clustering first calculates the dissimilarity/Euclidean distance between data points (in our case UMAP clusters of cells) and then merges the two closest clusters, and repeats this until there's only one cluster remaining, creating a dendrogram where the height of the branches indicates the distance at which the clusters were merged or split. This is a built-in command in Scanpy because it is a common analysis practice. Figure S2d shows that certain UMAP clusters are transcriptionally more similar to each other. Likewise, the dendrogram also shows that groups of UMAP clusters are more similar to some UMAP cluster groups than others, based on the calculated Euclidean distance. Our hierarchical clustering shows that, within the whole placenta dataset (Figure 1), CTB clusters 1, 6, and 10

were transcriptionally most similar to each other and were therefore grouped together (CTB group 1) and that this group was most similar to CTB group 2, containing CTB clusters 2, 4 and 8. To make our analyses clearer to the reader, we have changed the naming scheme from Clade to hierarchical clustering group throughout the manuscript.

4 *Describing a group of clusters as a "clade" is not helpful. "Clade" implies common ancestry which is meaningless in the context of cells that are all derived from the same zygote. I appreciate that this could be useful short hand but if this is to be used then some justification and caveats need to be provided.*

Response: We are sorry for the confusion and are not implying common ancestry, simply using "clade" to refer to a group of clusters. As noted above, we have now changed the naming scheme from Clade to hierarchical clustering group.

5a. *Line 208-212. The conclusions drawn about the basal and chorionic plate genes requires more explanation. The genes of interest are apparently upregulated in EVT or pre-STB clusters. However, it is not clear what "up regulated" means – the number of cells in each cluster varies so is the increase just due to there being more cells, or a more homogeneous cluster?*

Response: We have added statistical analysis to this claim as well as changed the wording. This line now reads: "We found that the BP-CTB genes were significantly more highly expressed in the EVT clusters (Wilcoxon rank-sum p-value < 0.001), and the CP-CTB genes were significantly more highly expressed (Wilcoxon rank-sum p-value < 0.001) in our pre-STB clusters (**Fig. S5d**)."

5b. *The term "up-regulated" also implies more than the authors intend – it implies a detected increase – ie something was low and then when measured later it was higher. However, what the authors actually mean is that something was simply higher in one cell (or cluster) than another when measured at the same time point.*

Response: We have replaced the word "upregulated" with "enriched" throughout the manuscript to more accurately indicate our meaning.

6. *My major reservation about the data presented is due to the low number of placental samples analysed. In the core experiments n=2 although for some experiments it is slightly higher. This is problematic although I fully understand the challenge of analysing more samples – tissue availability and the time and cost of more single cell RNAseq are not insignificant. However, the authors are drawing firm conclusion that there is more of one cell type at a given gestational age (6-8 weeks vs 12-14 weeks) or location in the placenta (basal vs chorionic plate). There will be sample to sample variation and heterogeneity within a single placenta. When one only has 2 samples it is not possible to make strong statements about any observed differences. This is a major weakness of the manuscript.*

Response: We understand the reviewer's reservations with our sample sizes. In our whole placenta analysis, we performed single cell RNA-seq on two samples from early first trimester placenta and two from late first trimester placenta, then used two additional placentas for our basal and chorionic plate analysis; however, for the basal and chorionic plate analysis, we also performed spatial transcriptomics on an additional three samples. Finally, we also performed scRNA-seq on three different TSC lines to compare to our primary placenta samples, then applied scRNA-seq analysis to four different timepoints of TSC derivation from two separate early first trimester placentas.

To address the reviewer's concern, we have now performed a comparable early-versus-late first trimester CTB analysis using, to the best of our knowledge, the only published single-cell dataset containing both timepoints, from Shannon et al. (2022; PMID: 35020896). This independent analysis recapitulated many of the CTB differences observed in our own dataset. These results are now presented

in Figure S3 and Table S6. We also acknowledge the limitations imposed by our sample size and have explicitly discussed these constraints in the Discussion section.

7. *Lines 184-5: The histological evaluation of the proposed progenitor cells – statements such as "more numerous", or "more enriched" (or better, simply "enriched") require some numerical assessment. This will not be robust when only 2 samples are available at each gestational age.*

Response: The histological evaluation in lines 184-185 was done in five samples from both the basal and chorionic group. To make this clearer to the reader, we have added this to the caption for Figure 2D.

8. *Lines 324-7: The authors describe experiments performed at 21% and 2% oxygen and describe these as "normoxia" and "hypoxia" respectively. The authors need to be careful with these terms – the first trimester placenta in vivo is in a physiologically low oxygen environment – it's low but normal so this is "normoxia" (explicitly not hypoxic although some of the published literature does use this term). However, the in vitro work described in this manuscript is performed at ambient oxygen. The text needs amendment to clarify - it's likely simplest not to refer to normoxia or hypoxia in the manuscript as the terms will likely cause confusion.*

Response: We have now changed these terms, from normoxia and hypoxia, to 21% oxygen and 2% oxygen, respectively.

9. *Line 135 (and elsewhere). The level of RNAs in a cell and hence those that distinguish different clusters depends on transcription and this is different in different cells (and clusters). However, the scRNAseq evaluates the level and a fixed point in time so is not a measure of transcription so it is inappropriate to say that cells are "were transcriptionally similar". Clearly transcription is a critical regulatory process but is not assessed directly by scRNAseq.*

Response: We respectfully submit that the terminology used in our manuscript is broadly used by other groups when describing scRNA-seq data. However, to make the manuscript as accurate as possible we have eliminated the use of the phrase "transcriptionally similar" where possible.

Minor points
Fig 1 b please use a single continuous colour scale to shoe expression level

Response: We respectfully submit that the two-color sequential color maps allow the reader to better visualize the expression levels.

Fig 2d is much much too small

Response: This figure shows both a low-power image (in order to capture both the chorionic and basal plate for comparison in the same image) as well as a high-power inset image (in order to better capture the signal in distinct cell types). We have added further annotation to the figure in order to clarify the different structures.

*Line 227 and elsewhere: chorionic **plate** cells*

Response: This has now been updated.

Reviewer #3 (Remarks to the Author):

This is a descriptive single cell RNA-seq paper. The authors profile the transcriptomes of placentas from

2 early (6-8 weeks) and 2 late (12-14 weeks) first trimester, with early placentas further separated into basal and chorionic fractions. They also measure the transcriptomes of TSC cultured in vitro in 2D. Based on these data, the authors perform comparisons between early and late placentas, between chorionic and basal plate, as well as between in vivo trophoblast cells and TSC.

Response: We in fact profile a total of six first trimester placentas by single cell RNA-seq: 2 early (6-8 week) and 2 late (12-14 week) first trimester placentas are profiled at the whole placenta level (Figure 1), while two additional early (6-8 week) first trimester placentas are used to isolate CTB from chorionic and basal plates, then subjected to single cell analysis (Figure 3). Three additional early (6-8 week) first trimester placentas were used for spatial transcriptomics (Fig S5). We also profiled 3 previously-derived TSC lines, as well as two additional TSC lines during the process of derivation from early (6-8 week) first trimester placentas by single cell RNA-seq. Other than simply comparing in vitro TSC, cultured in 2D, to their cells-of-origin by computational methods, we also culture the cells, in both 2D and 3D, in order to further confirm the differences between TSC and CTB. For comparison, Arutyunyan et al.'s 2023 Nature paper (PMID: 36991123) was based on analysis of essentially ONE early gestation placenta sample, while Shannon et al.'s Dev Cell paper (PMID: 38359834) used samples from only 3 patients, with additional comparison to previously-published data.

Although they found differences from these comparisons and provided another resource for the field, it is not clear what is new and different from other similar studies. In particular, in Shannon Dev Cell 2024 and Arutyunyan Nature 2023, similar comparisons are performed and these papers have also looked at primary trophoblast organoids and TSC in 3D. Moreover, both the rationale for the studies performed and summaries of the findings need to be more succinctly and clearly explained. The paper is dense and difficult to read. One focus is on the identity of the trophoblast progenitor(s) but this is not resolved nor where they reside.

Response: As described above, our sample numbers exceeded those published by Arutyunyan et al. and Shannon et al. In addition, as now even further emphasized in the revised manuscript, this is the first study to: 1) compare chorionic vs. basal plate CTB, both by single cell RNA-seq and spatial transcriptomics; and 2) profile TSC, ***cultured in 2D***, by single cell RNA-seq, and compare them to their cells-of-origin. We have now clarified the methods performed in each figure using a cartoon summary, and have re-focused the manuscript on the two above, novel points.

Major concerns:

1. *There are many 'clusters' and 'clades' mentioned in Figs. 1, 3, 4. The naming of these clusters (e.g., CTB1, CTB2) is confusing. This makes each cluster unclear and difficult to compare across different figures (for example, between Fig. 1d and Fig. 3a). How do the CTB subclusters align across the three figures (Fig. 1d, Fig. 3a, and Fig. 4a)? Consistent terminology and clearer alignment are needed. In addition, it is unclear how these clusters correspond to previously reported trophoblast subtypes, and what is new and different from previous characterizations.*

Response: We have changed the term clades to "groups" and changed the CTB group names for the BP and CP scRNA-seq to CTB groups A and B, so as to distinguish them from the whole placenta CTB groups 1-3. Our CTB naming scheme was chosen in order to assign cells in an unbiased way, allow for the initial state cells to be computationally assigned, to find differences and similarities between early and late first trimester CTB, and to determine the similarity and differences between our TSC and initial state CTB. We have now compared our data to both Arutyunyan et al's. 2023 (PMID: 36991123) and Vento-Tormo et al's. 2018 (PMID: 30429548) manuscripts.

First, we scored the top 200 genes from each of our CTB clusters with the data published in Arutyunyan et al., the results of which are shown in UMAPs in Fig. S2e. This dataset is focused on trophoblast cell types but notably comes primarily from a single placenta that is 9 weeks gestational age

(GA), which is abnormally attached to the underlying uterus (e.g. part of a gravid hysterectomy for first trimester placenta accreta spectrum disorder). Our CTB group 3 cluster, which contains a high percentage of our late first trimester placenta and is therefore arguably most similar in GA to their data, not surprisingly corresponds to their VCT cluster. Our CTB group 1 cluster, the cluster that contains our initial state CTB and a high percentage of our early first trimester placenta cells, generally corresponds to their proliferating VCT cluster which they mention contains, "...known stem and progenitor cell markers". Of note, in their trajectory analysis, they manually set their initial state as their VCT cluster instead of computationally assigning it as we have done here. Our CTB group 2 cluster is distributed across several VCT clusters and although does not express markers of non-trophoblast cells, EVT, or STB, it exhibits lower expression of many canonical CTB markers. This may reflect the high proportion of early first trimester placenta cells within this cluster. Accordingly, we have revised the manuscript to clarify that, although CTB group 2 cells show reduced expression of several canonical CTB markers, they display high expression of *VGLL1*, a highly specific trophoblast marker that is most strongly expressed in villous CTB (PMID: 29361559; 41284866).

We also scored the data from Vento-Tormo et al. in the same way; the results are shown below. However, this study focused less on trophoblast cell types and therefore lacks granularity for further assignment of our CTB subclusters. Nevertheless, we have now used this dataset to highlight a subset of villous trophoblast, similar to our CTB group 2, characterized by high VGLL1 and low EGFR expression, as shown in Fig. S2f.

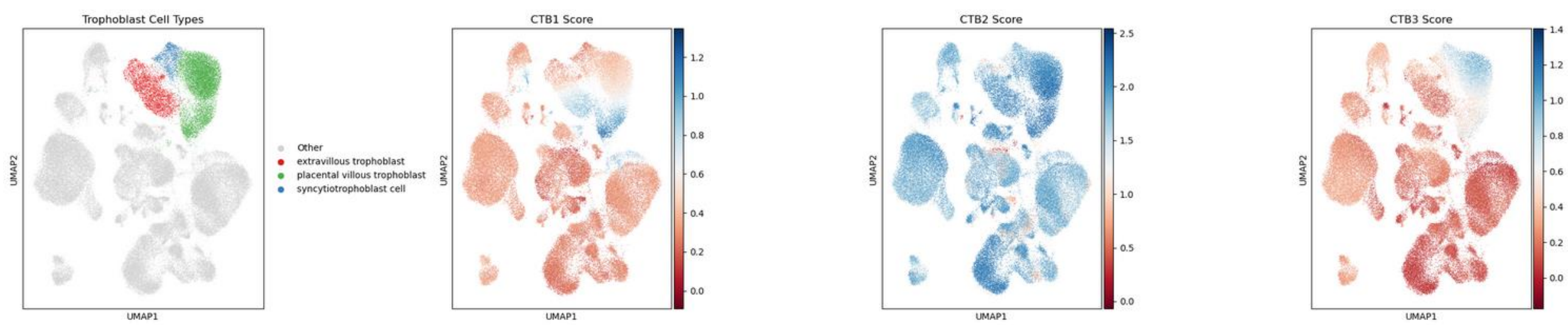

2. *There are other public databases that have scRNA-seq data from placentas throughout the first trimester. Although this data has not been studied to show gestational-age changes, it should be analysed to incorporate into the data here. This would also strengthen the findings as only 2 samples from early or late gestation have been used here*.

Response: We have used the meta analysis of multiple different scRNA-seq papers, performed by Derisoud et al. (PMID: 38478759), in order to annotate our clusters in this manuscript, and have now further clarified the annotation of different clusters/clades, applying uniform nomenclature, across our figures. Additionally, as noted in response to Reviewer #2, we performed a comparable early-versus-late first trimester CTB analysis using, to the best of our knowledge, the only published single-cell dataset containing both timepoints, from Shannon et al. (2022; PMID: 35020896). This independent analysis recapitulated many of the CTB differences observed in our own dataset. These results are now presented in Figure S3 and Table S6. Also, as stated under Reviewer #3, point #1 above, we have also compared our cells to Arutyunyan et al's. (2023, PMID: 36991123) snRNA-seq data (Fig. S2e, Fig. S4c, Fig. 4g) and Vento-Tormo et al's. (2018, PMID: 30429548) scRNA-seq data (Fig. S2f). Finally, we also acknowledge the limitations imposed by our sample size and have explicitly discussed these constraints in the Discussion section.

3. *The authors identified a CTB initial state expressing HMMR, TROAP, CENPA and MXD3. HMMR and CENPA are G2M phase genes. Are they defining CTB initial states or just proliferating cells? How do these cells compare to trophoblast subtypes described in previous studies? Do they correspond to*

proliferative CTBs previously reported? Additionally, the expression of HMMR and TROAP appears weak in the initial state (Fig. 2c).

Response: Please see our response to Reviewer 1's comment #2 above as well as our response to your first comment. To expand our response to comment #1, Arutyunyan et al. defined VCT and VCT_p clusters using the following markers: TP63, CDH1, LRG5, L1TD1, WLS, TNIK, SEMA3F, NRP2, and BCAM. Many of these markers, such as BCAM, are found in not only their VCT and VCT_p clusters but also in their CTB column cells (VCT_CCC) and their SCT precursors, VCT_fusing. In fact, in their methods, they write that they combine the VCT and VCT-p clusters when they do their differential expression analysis and manually select their VCT cluster as their initial state when performing their trajectory analysis. Instead of manually selecting a cluster as our initial state, we wanted to computationally define our initial state cells in the most unbiased way possible and not limit ourselves to a set of predefined cells heavily reliant on a selected cluster resolution setting. We therefore used CellRank's Generalized Perron Cluster Cluster analysis (GPCCA) with the VelocityKernel to compute a transition matrix based on RNA velocity and identify macrostates of cellular dynamics, including the initial state. After computationally defining a small set of initial state cells, we asked what genes were differentially expressed in these cells compared to all of our other trophoblast cells. HMMR and TROAP were two novel genes that were, not necessarily most highly expressed, but most specifically expressed in our initial state cells compared to our other trophoblast cells. Below, we compared our initial-state cells to the dataset published by Vento-Tormo et al. (2018; PMID: 30429548). Our initial state scoring is highest within their CTB cluster; however, their study was not specifically designed to resolve CTB subtypes. We also compared our initial state signature to the snRNA-seq dataset from Arutyunyan et al. (PMID: 36991123). Cells with the highest initial-state scores were enriched within Arutyunyan's VCT_p cluster, which, as noted in our response to Comment #1, contains "known stem and progenitor cell markers." These results are now presented in Figure S4c.

Vento Tormo et al:

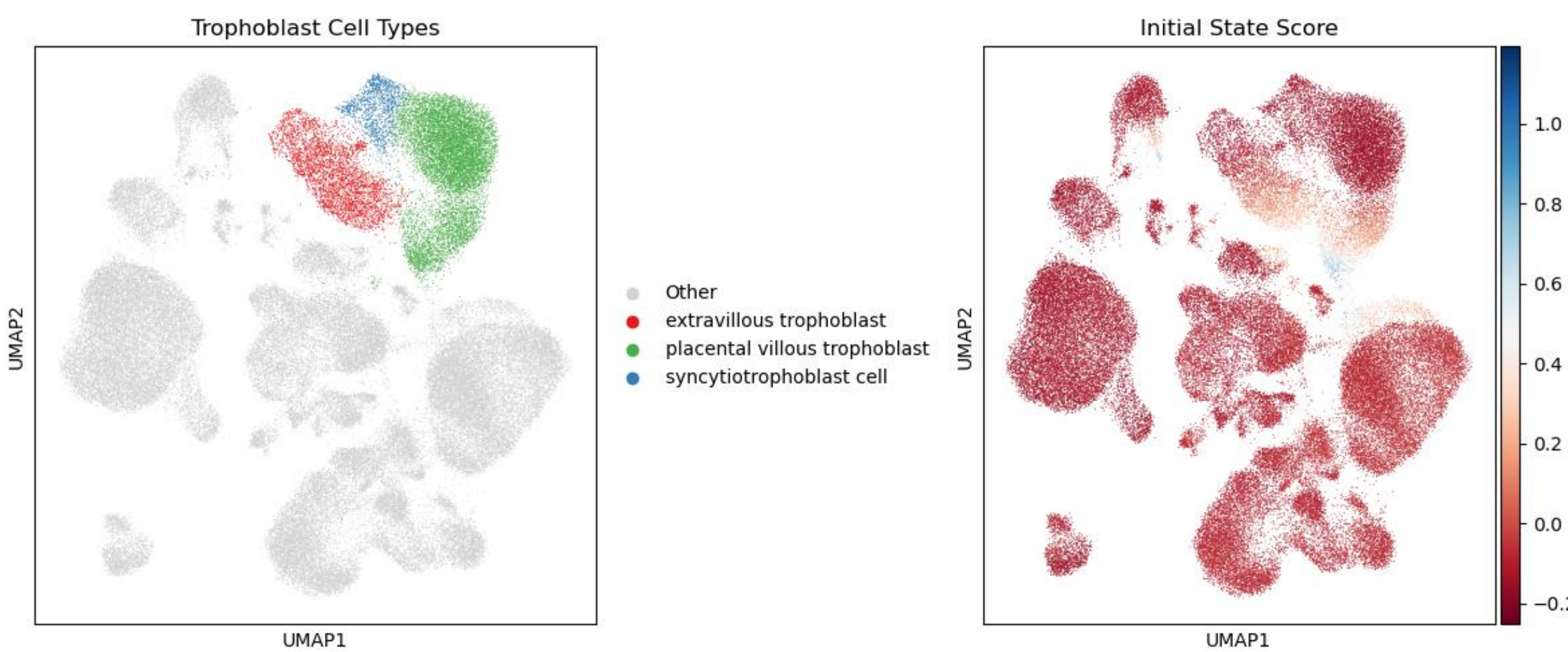

4. *Marsh et al (PMID: 35796428) (not cited) also performed scRNA-seq on the two regions (villous and smooth chorion) in the second trimester. How do the findings in this study compare to those from the second trimester?*

Response: As mentioned in the manuscript, and above, the goal of this study was to understand the origin of TSC, which were first derived from early first trimester CTB, and from the villous placenta. Therefore, comparison to second trimester CTB, and especially to smooth chorion (cells within the fetal membranes and not within chorionic villi), is beyond the scope of this manuscript.

5. *The section comparing TSC is not novel and is poorly performed. Okae did not separate cells based on ITGA6+ expression. Firstly, the flow cytometry needs to show the scatter plot, gating strategy, live/dead marker and negative control and then representative histograms of all markers. The error bars seem very small for 6 lines as well? It is now well established that TSC have a high proportion of ITGA2+ cells compared with trophoblast from normal placentas. The data on ITGA2, ITGA5 and ITGA6 expression (including IHC and ISH) has also all been described previously. Culturing primary trophoblast and analysing integrin expression is also not novel and the culture in different oxygen levels introduces another variable.*

Response: As mentioned in the manuscript, and above, this is the first manuscript to subject TSC (cultured in 2D) to detailed profiling, including by single cell analysis. Our comparison is focused on the comparison between TSC and CTB, and in fact shows a clear distinction between the in vitro-derived cells and "initial state" CTB. According to the methods section in Okae et al.'s 2018 Cell Stem Cell article (PMID: 29249463), they did indeed derive TSC from ITGA6+ CTB. Table S3 in that manuscript shows that all of their TS^{CT}, were derived from (6 to 9 week) CT cells and their methods section indicates that, "CT cells were immunomagnetically purified using the EasySep PE selection kit and a PE-conjugated anti-ITGA6 antibody." Other groups have indeed shown TSC to be enriched in ITGA2; however, our manuscript is the first to show the quick gain of proximal column trophoblast markers, ITGA2 and ITGA5, during the derivation process, again firmly validating the selection of a relatively rare in vivo cell type when transitioning from primary CTB to TSC. The IHC and ISH for these markers, particularly the dual ISH for ITGA2 and ITGA5 (Fig S9e), is shown here to emphasize the rarity of such dual-positive cells in vivo. Finally, as stated in response to reviewer 1's comment #6 above, the culture in different oxygen levels follows our previous publication (Wakeland et al. Am J Pathol. 2017, PMID: 28167044) and is done here to, again, compare primary CTB to TSC—going beyond computational analysis and further validating the phenotypic differences between these cell states.
Regarding the flow cytometer analysis, cells were always gated to eliminate dead cells and cell debris. These TSCs are well-established lines and therefore, the expression of EGFR, ITGA6, ITGA5, ITGA2, HLA-G, and ITGA1 is pretty consistent across all six lines and therefore, the error bars are very small. Lastly, we combined Okae's (2018) bulk RNA-seq data from their four original TSC lines with our TSC and early first-trimester placental dataset to assess transcriptomic similarity between their TSCs and ours. Using correlation analysis, UMAP distance measurements, and a permutation-based test, we found that the published Okae TSC lines most closely resemble the TSC1 cluster, representing the majority of our TSC (see Figure 4b). These results are now provided in Supplementary Table S13.

6. *The authors show that there are 3 clades of CTB. Why are the three clades not clustered together in Fig. 1c? Moreover, clade 2 (CTB2, 4, 8) has very low expression of CTB markers. The authors need to check whether this clade represents a truly distinct subpopulation of CTB. It is quite possible that they are low-quality cells with a much lower number of genes detected (usually accompanied by a higher percentage of mitochondrial genes), which would separate them from other clusters in the UMAP. This issue is also present in several other figures in the paper, including CTB2, 6 in Fig. 3a, and CTB3, 5 in Fig. 4a.*

Response: Please see methods, which clearly delineate QC for single cell analysis, excluding low-quality cells due to high percentage of mitochondrial genes. Doublet scores and mitochondrial expression was again checked to ensure that these cells are of high quality. Clade 2 (now named CTB group 2), consisting of CTB clusters 2/4/8 in the whole placenta, has low levels of some CTB markers (such as EGFR) but high levels of other, rather specific, markers of CTB, including VGLL1. As noted in our response to comment #1, we believe that these cells may represent CTB present in early first trimester placenta. We are currently evaluating these cells further; however, this signature is noted in some of the trophoblast cells in Vento-Tormo's data. These results have now been added in Fig. S2f.

7. *Are the trophoblast cells shown in Fig. 1d from chorionic or basal plate or both?*

Response: Figure 1 is looking at data from the whole placenta (early vs. late first trimester), from which trophoblast were computationally separated and compared across these two different gestational timepoints. We have now clarified the methods used in each figure, using a cartoon illustration at the top of each figure.

8. *Cluster 21 in Fig. 1a is likely to be doublets as it expresses VIM (which is absent in trophoblast cells)*.

Response: We agreed with you and in fact removed this cluster from further analysis, as clearly stated in the results. We wrote: "We were interested exclusively in the trophoblast clusters (0, 2, 3, 4, 5, 8, 10, 11, 13, 14, 17, 19) and removed all non-trophoblast clusters (Fig. S2a)." This cluster was never part of our trophoblast analyses.

9. *The 'non-trophoblast cells' have been defined with markers for Hofbauer cells, fibroblasts and endothelial cells. However, in these surgical samples there is always contamination from maternal blood and decidual cells. Additional markers are needed to encompass all leukocytes (CD45, CD3, CD19, CD56) and glandular epithelial cells.*

Response: We in fact used HLA-A and -B as markers of non-trophoblast, which should apply to both fetal as well as maternal cells. We have also looked at the markers mentioned above and they did not identify additional non-trophoblast cells.

10. *Lines 185-186: the authors concluded that HMMR positive cells "are more numerous in early first trimester placentas, and significantly more enriched at the basal, compared to chorionic plate" based on Fig. 2d. This needs quantification*.

Response: This is clearly visible from the ISH images in Figure 2d, where more brown signal is present in the 6-week sample and near the basal plate; this figure has now been further annotated to clarify the structures.

11. *Lines 209-210 and Fig. S4c: it is unclear that CP-CTB genes were upregulated in pre-STB*.

Response: We have changed Fig. S4c (now Fig. 5d) (to violin plots) and clarified the description in the results to make this point more clear.

12. *Line 218: given that ASCL2 is an EVT marker gene, why did the authors use ASCL2 to define BP cells?*

Response: ASCL2 is also expressed in villous CTB of anchoring villi (see Figure 2b in Wakeland et al. Am J Pathol. 2017; PMID: 28167044).

13. *Fig. 3b: It is unclear how the size factor was calculated, and no details are provided in the Methods section*.

Response: The size factor calculation details have now been added to the methods section.

14. *Fig. 5b: why are the cells separated by days? It is unclear what cell types are present*.

Response: This figure is showing the TSC derivation process, which involves culture of primary CTB, isolated from 6-8 week gestation placenta by Percoll gradient centrifugation, in TSC media in 2D over 10 days in culture.

15. *The colour scheme used is confusing (red indicates low expression and blue indicates high expression)*.

Response: The color scheme is python's colormap, cmap=RdBu, a standard diverging colormap designed to highlight deviations from a central neutral value. We have placed legends alongside the figures to help readers. This color scheme is common due to the fact that red light has lower energy and frequency and blue light has higher energy and frequency and can also be seen in weather maps where low pressure is depicted with red and high pressure with blue.

16. *There is a paper comparing the trophoblast from basal and chorionic villi, mainly focused on the derived organoids, but they also do scRNAseq on the tissue (https://doi.org/10.1101/2024.11.19.624345) on BioRxiv*.

Response: This manuscript is looking at smooth chorion (trophoblast within the fetal membranes) and chorionic villi from term placenta—data which are worth comparison to the Marsh et al. study, but again, irrelevant to the goal of our study (which was the comparison of TSC to their early first trimester cells-of-origin within the villous placenta, not to second or third trimester cells nor to trophoblast in the smooth chorion/fetal membrane region of the placenta).

17. *In Fig.1d it looks like both chorionic and basal plate are present, but the panels are really too small (maybe also a dotted line separating the two regions would help) and how they then claim the cells are more numerous in early compared to late is not clear.*

Response: The reviewer is likely referring to Figure 2D; as stated above, this figure has now been further annotated to clarify the findings.

18. *What is the sex of the samples?*

Response: We've now added this to the supplementary table.

REVIEWERS' COMMENTS:

Reviewer #1 (Remarks to the Author):

The authors have addressed all of my concerns through their explantation in the rebuttal letter and by adding additional supplementary figures. I do not have any major concern about the study.

We thank the reviewer for their help throughout the review process.

Reviewer #2 (Remarks to the Author):

Point # 2 In my earlier review I commented on the use of the phrase “spontaneous differentiation”. The authors have responded to this and they give what they think is an analogy and they state, “as an analogy, mouse TSC, upon removal of the maintenance factors FGF4 and MEF-conditioned media (or activin A), spontaneously differentiate into both labyrinthine and junctional zone trophoblast.” This is in fact a perfect example that the use of the word spontaneous is incorrect. The authors acknowledge that the differentiation from stem cells requires the removal of FGF4 and conditioned media. Hence it is not spontaneous - it is induced by the intervention of removing these factors. Therefore, my comment in my earlier review remains - it is inappropriate to use the word “spontaneous” when the observed change requires the addition or removal of a factor or stimulus. Hence the wording still needs to be changed.

We thank the reviewer for this clarification regarding the use of the term “spontaneous differentiation.” We now better understand the reviewer’s concern that differentiation initiated by removal of maintenance factors is not strictly spontaneous, as it requires an experimental intervention. Our intended use of the term was to distinguish differentiation occurring in the absence of lineage-directed cues from differentiation induced toward a specific trophoblast fate. To avoid ambiguity and align with the reviewer’s recommendation, we have removed the term “spontaneous” throughout the manuscript.

Points # 3,4 and 5 I agree, the revised wording clarifies this.

Thank you for confirming.

Point #6 This was my major concern – the very low number of samples analysed and the strength of the conclusions drawn. The authors have added new analyses of other data sets to support their work. These are useful additions and are reassuring as they do broadly support the conclusions drawn. Nonetheless I would still like the conclusions to be asserted more cautiously. For example these phrases in the abstract and discussion need tempering: “We found notable distinctions” (line 31); CTB progenitors

were enriched…. basal plate CTB were biased toward EVT…" (line35-6); "in-depth transcriptomic perspective….. have significant implications….." Line 521-2);

We thank the reviewer for this thoughtful comment regarding the interpretation of findings derived from a limited sample size. We agree that the conclusions should be stated more cautiously. Accordingly, we have revised the wording throughout the manuscript, abstract, results, and discussion to better reflect the exploratory nature of these findings while still conveying the observed trends, which are additionally supported by independent published datasets.

Point #7 Simply pointing out that n=5 does not address this. Lines 190-1 explicitly state, "We confirmed that these cells were more numerous…." To conclude they were "more numerous" you need to count them in several samples (5 would probably be sufficient) and then asses the apparent difference statistically. I suspect the authors have not done this so softening the hard conclusion to "appeared to be more numerous" would solve the problem – but of course make the conclusion less interesting.

We thank the reviewer for this suggestion and agree that the original wording overstated the strength of the conclusion given the limited quantitative analysis. Accordingly, we revised the text from "were more numerous" to "appeared to be more numerous" to more appropriately reflect the observational nature of these data.

Point #8 ok

Thank you for confirming.

Point #9 The use of the phrase "transcriptionally similar". My point is somewhat pedantic and just because other papers use the phrase somewhat loosely is not a justification for propagating the error. Precision matters in science and RNAseq does not measure transcription – it measures the level of transcripts in a sample (or cell). Clearly this level critically depends on transcription but this is not measured – only inferred. I appreciate it that the authors have revised the wording accordingly.

Thank you for confirming.

Reviewer #3 (Remarks to the Author):

The authors have addressed most of the concerns. However, several minor issues still need to be further clarified or corrected.

1.Figures 1a, 3a, and 4a have not been cited in the main text.

Thank you for pointing this out. We have now cited these in the main text.

2.In Tables S5 and S9, does "CTB1–3" refer to CTB groups 1–3 or clusters 1–3? The terminology is currently confusing.

We're sorry for the confusion. Table S5 relates to Fig. 1E and Table S9 relates to Fig. 2a and the names of the columns in the table correspond to the clusters shown in the figures.

3.In lines 184–185, TET1, KMT2A, and CENPA are not transcription factors. Similarly, in Figure S12e, several listed genes are also not transcription factors.

We thank the reviewer for this comment. These genes were identified from the HumanTFDB resource (http://humantfs.ccbr.utoronto.ca), a curated database of known and likely human transcription factors and transcription-associated regulatory proteins. However, we agree that TET1, KMT2A, and CENPA are not widely considered canonical transcription factors and could be more accurately classified as chromatin-associated regulatory factors rather than transcription factors. Accordingly, we have revised the text and Figure S12e to use broader terminology where appropriate.

4.In Figure S4b, what does "CTB-Clade" refer to?

We are sorry for the confusion. In an earlier version of the manuscript, we used the term "Clade" to represent groups of clusters that clustered together using Euclidean distance in a dendrogram. This has now been changed to "group" in the figure.

5.The placements of panel label "b" in Figure 3 and panel labels "c", "g", and "h" in Figure 5 are not appropriate.

Thank you for your attention to this. Figure 3a has now been added to the text so Figure 3b should make more sense. For Figure 5, the figure labels have been moved around to make it more reader friendly.

6.In line 352, "Figure S11b" may be incorrect. Should this instead refer to Figure 12b?

Thank you for your attention to detail. This was supposed to read "S12a and S12b". This has now been corrected.
