# Supplementary material for: Single-cell transcriptomics reveals differences between chorionic and basal plate cytotrophoblasts and 2D-cultured trophoblast stem cells
Source: Commun Biol. 2026 Jun 10;9:1234. doi: 10.1038/s42003-026-10460-0 (PMC13594312; doi:10.1038/s42003-026-10460-0)
Supplement: Supplementary file 2 [file 42003_2026_10460_MOESM2_ESM.pdf]

Supplementary Figures for

Single-cell transcriptomics reveals differences between chorionic and basal plate cytotrophoblasts and 2D-cultured trophoblast stem cells

Robert Morey[1,2,3,*], Francesca Soncin[1,2,3,*], Sampada Kallol[1,2,3], Nirvay Sah[1,2,3], Zoe Manalo[1,2,3], Tony Anh Bui[1,2,3], Jaroslav Slamecka[1,2,3], Virginia Chu Cheung[1,2,3], Donald Pizzo[1], Daniela F. Requena[1,2,3], Ching-Wen Chang[1,2], Omar Farah[1,2], Ryan Kittle[1,2,3], Kelly Lam [1,2,3], Morgan Meads[1,2,3], Mariko Horii[1,2,3], Kathleen M. Fisch[3,4], Mana M. Parast[1,2,3,6]

[1]Department of Pathology, School of Medicine, University of California San Diego, La Jolla, CA, 92093, USA.
[2]Sanford Consortium for Regenerative Medicine, La Jolla, CA, 92093, USA.
[3]Center for Perinatal Discovery, University of California San Diego, La Jolla, CA, 92093, USA.
[4]Department of Obstetrics, Gynecology, and Reproductive Sciences, School of Medicine, University of California San Diego, La Jolla, CA, 92093, USA.
[*]These authors contributed equally
[6]Correspondence: mparast@health.ucsd.edu

This file includes:

Supplementary Figures S1-S12

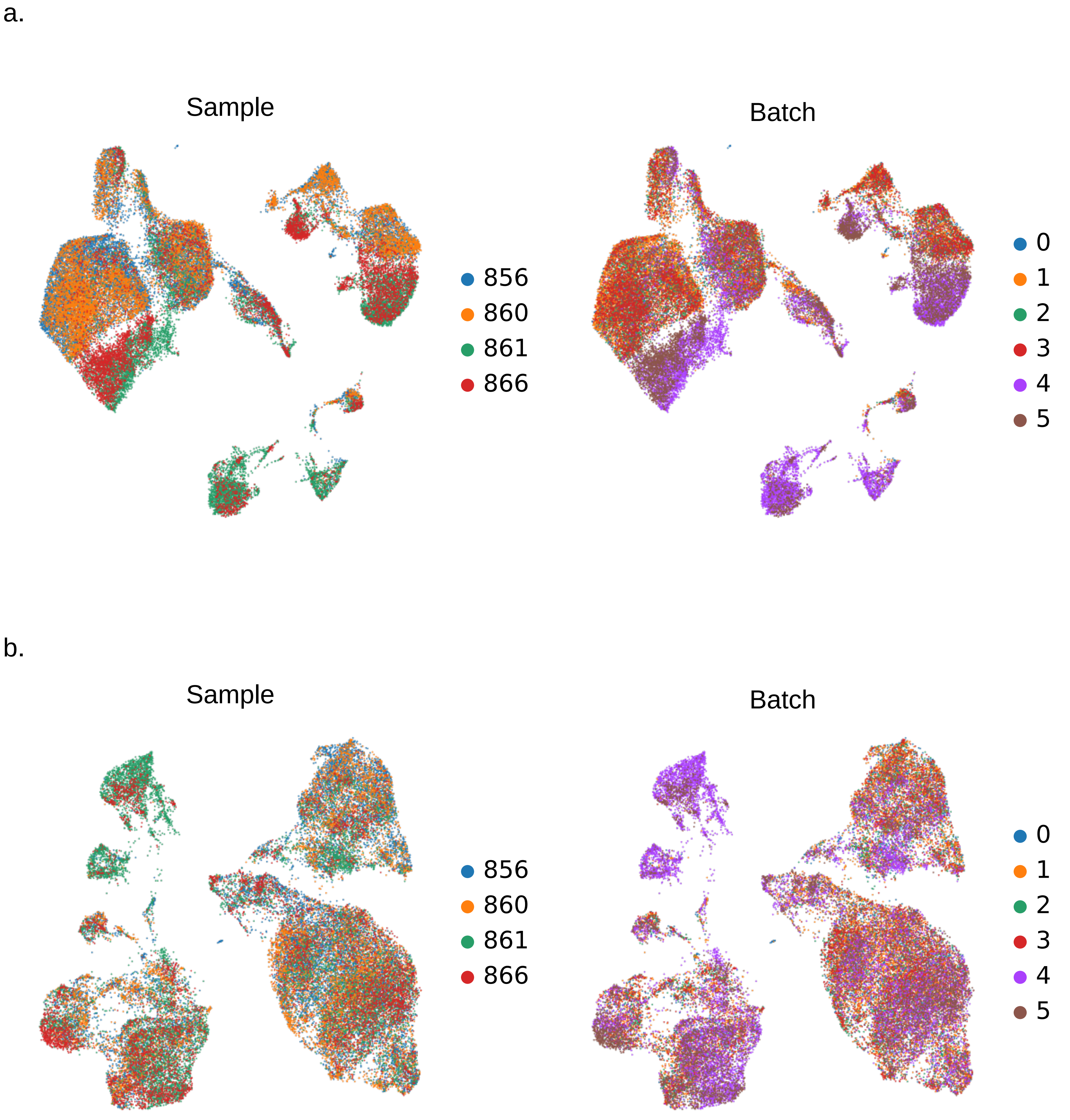

Figure S1: First trimester placental single cell RNA-seq: Integration UMAPs. (a) UMAPs of placental samples before integration colored by the patient number (left) and batch number (right). (b) UMAPs of placental samples after integration colored by the patient number (left) and batch number (right).

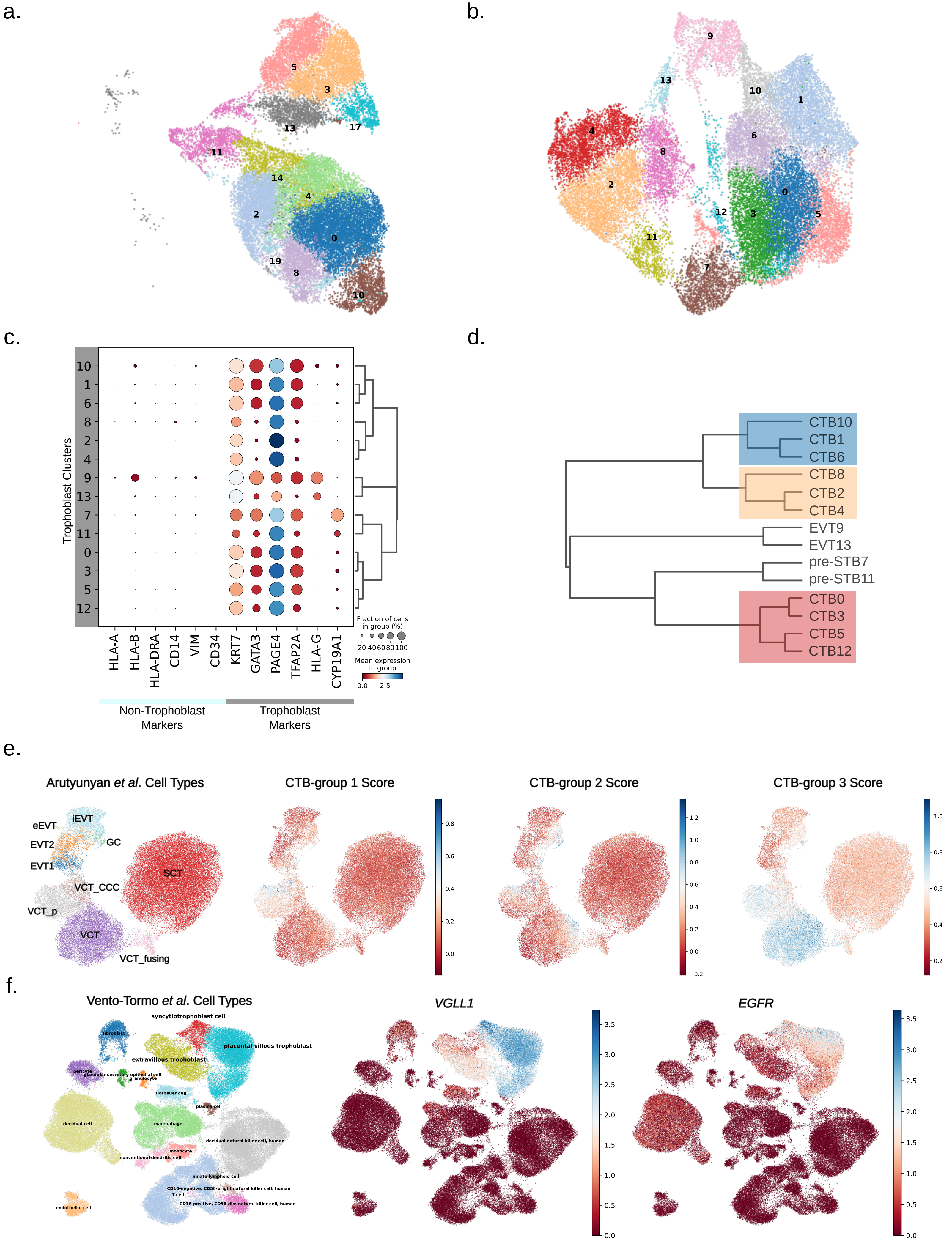

a.
b.
c.
d.
e.
f.
Trophoblast Clusters
HLA-A
HLA-B
HLA-DRA
CD14
VIM
CD34
KRT7
GATA3
PAGE4
TFAP2A
HLA-G
CYP19A1
Fraction of cells in group (%)
20 40 60 80 100
Mean expression in group
0.0 2.5
Non-Trophoblast Markers
Trophoblast Markers
CTB10
CTB1
CTB6
CTB8
CTB2
CTB4
EVT9
EVT13
pre-STB7
pre-STB11
CTB0
CTB3
CTB5
CTB12
Arutyunyan et al. Cell Types
CTB-group 1 Score
CTB-group 2 Score
CTB-group 3 Score
eEVT
iEVT
EVT2
GC
EVT1
SCT
VCT_CCC
VCT_p
VCT
VCT_fusing
Vento-Tormo et al. Cell Types
VGLL1
EGFR
syncytiotrophoblast cell
fibroblast
placental villous trophoblast
extravillous trophoblast
pericyte
glandular secretory epithelial cell
granulocyte
Hofbauer cell
plasma cell
decidual cell
macrophage
decidual natural killer cell, human
monocyte
conventional dendritic cell
innate lymphoid cell
CD16-negative, CD56-bright natural killer cell, human
T cell
CD16-positive, CD56-dim natural killer cell, human
endothelial cell

Figure S2: First trimester placental single cell RNA-seq: Removal of non-trophoblast clusters and reclustering of trophoblast cells. (a) UMAP following the removal of clusters in **Fig. 1b** deemed to be non-trophoblast cells (**Fig. 1c**). (b) Reclustering of cells after removal of most of the non-trophoblast cells. (c) Dot plot and hierarchical clustering showing expression of trophoblast- and non-trophoblast-associated genes in reclustered UMAP shown in (b). (d) Dendrogram showing the transcriptional similarity between clusters in UMAP shown in (b), and annotated using dotplot shown in F**ig. 1d**, with groups colored by the cluster color of UMAP in **Fig. 1e** following the combining of clusters. (e) UMAP visualizations of snRNA-seq data from Arutyunyan *et al*[1] depicting the clustered and annotated trophoblast cell subtypes (left side) and the scores of the top 200 differentially expressed genes from each of the CTB groups found in **Fig. 1e**. (f) UMAP visualization of scRNA-seq data from Vento-Tormo *et al*[2] depicting the annotated cell types for each cluster (left side) and the expression of the CTB markers *VGLL1* and *EGFR*.

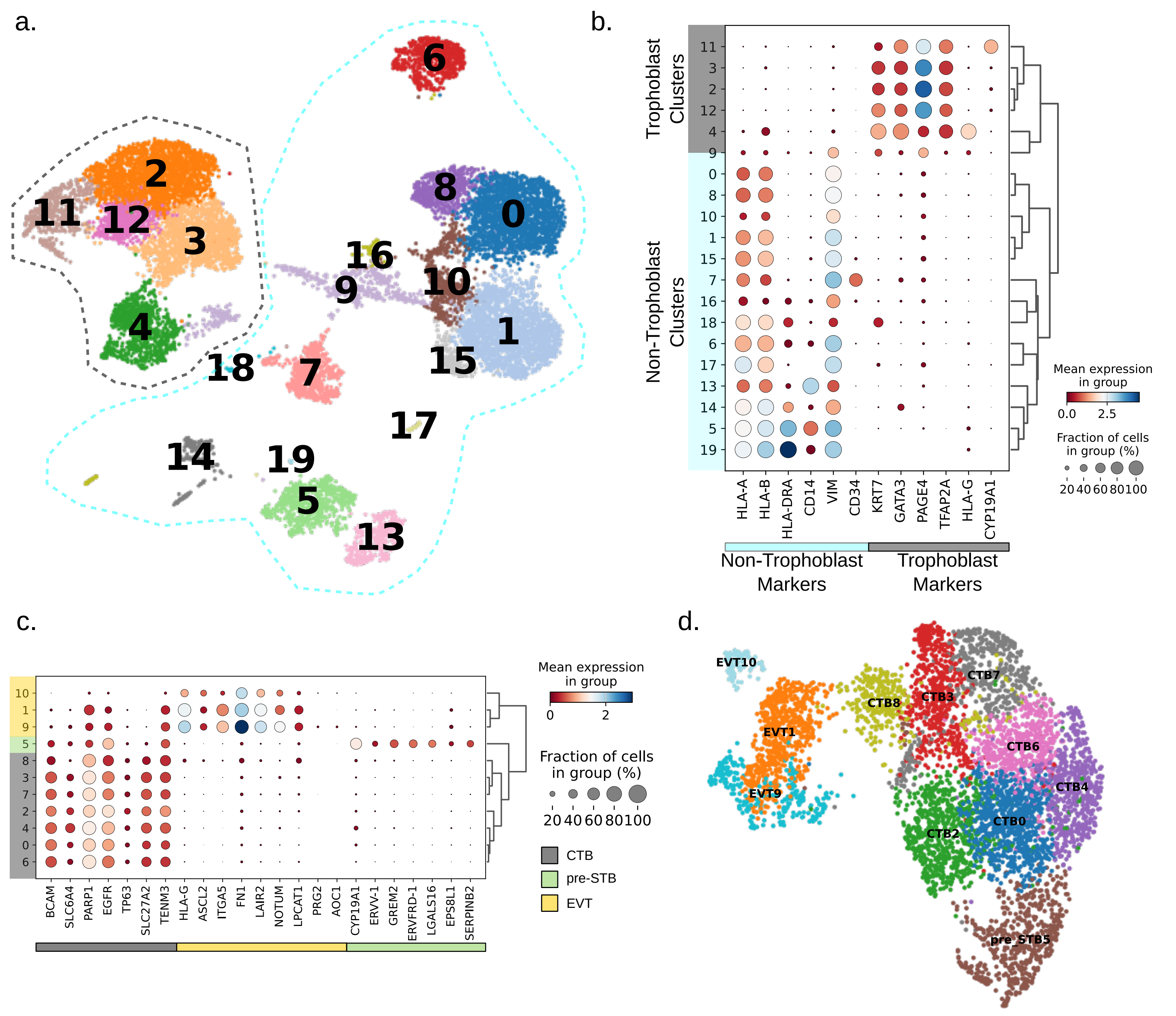

Number of cells per cluster

	CTB0	EVT1	CTB2	CTB3	CTB4	pre_STB5	CTB6	CTB7	CTB8	EVT9	EVT10
Early 1st Trimester	334	352	307	195	351	181	146	182	113	138	102
Late 1st Trimester	495	298	342	433	233	375	366	301	207	136	9

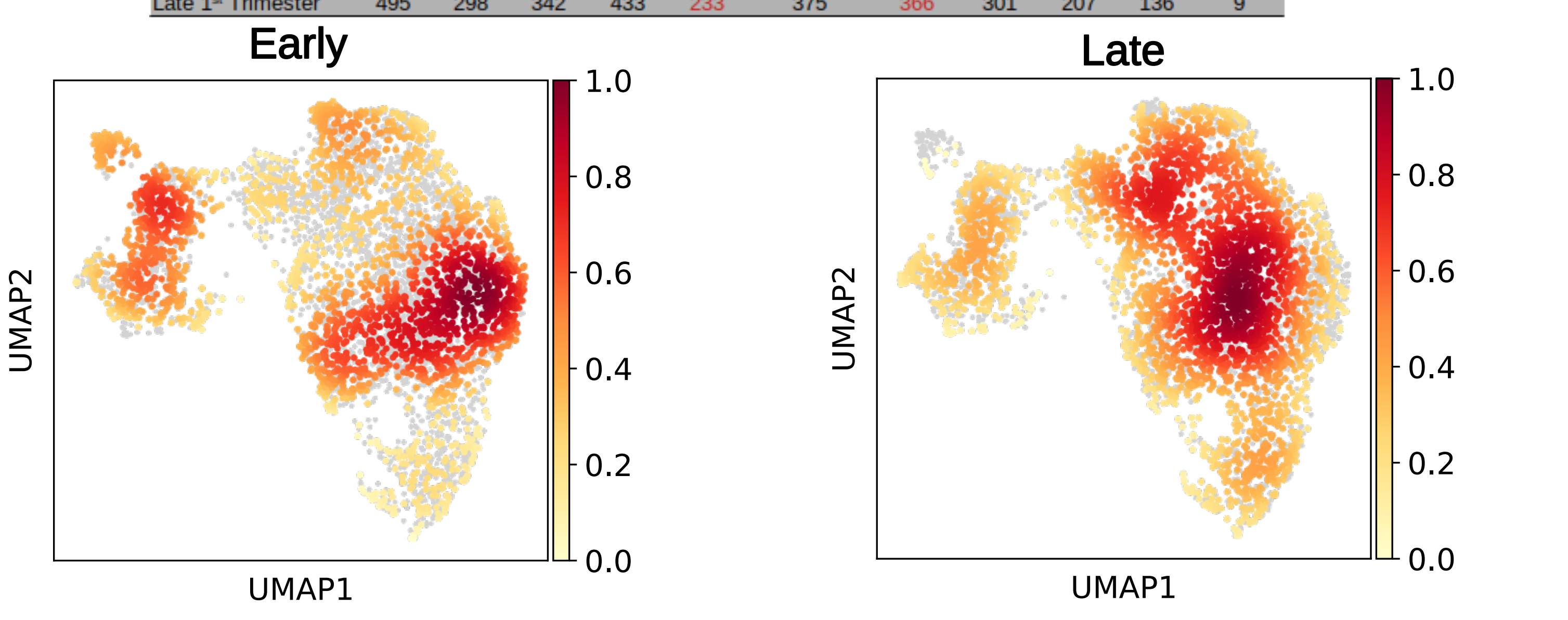

Figure S3: Reanalysis of first trimester placenta single cell RNA-seq from (Shannon et al.)[3]: Removal of non-trophoblast clusters, reclustering and annotation of trophoblast cells. (a) UMAP of all cells from four early first trimester and three late first trimester placenta. The grey dotted line marks trophoblast clusters while the light blue dotted line indicates clusters with non-trophoblast marker expression. (b) Dot plot and hierarchical clustering corresponding to UMAP clusters in part (a), showing the mean expression and fraction of cells expressing various trophoblast (grey) and non-trophoblast (light blue) marker genes. (c) Dot plot and hierarchical clustering corresponding to UMAP clusters in part (d) showing the mean expression and fraction of cells expressing trophoblast cell type-specific markers (CTB in grey, EVT in yellow, and pre-STB in green). (d) UMAP of trophoblast cells after removal of non-trophoblast cells in part (a) and annotated based on cell type specific gene expression shown in part (c). (e) UMAPs depicting the cell density in early (left) and late (right) first trimester placentas. Table (top) depicts the number of cells in each cluster from either the early or late first trimester placentas. Note: Clusters CTB4 and CTB6 (highlighted in red font), were used to represent cells in early and late first trimester placentas, respectively.

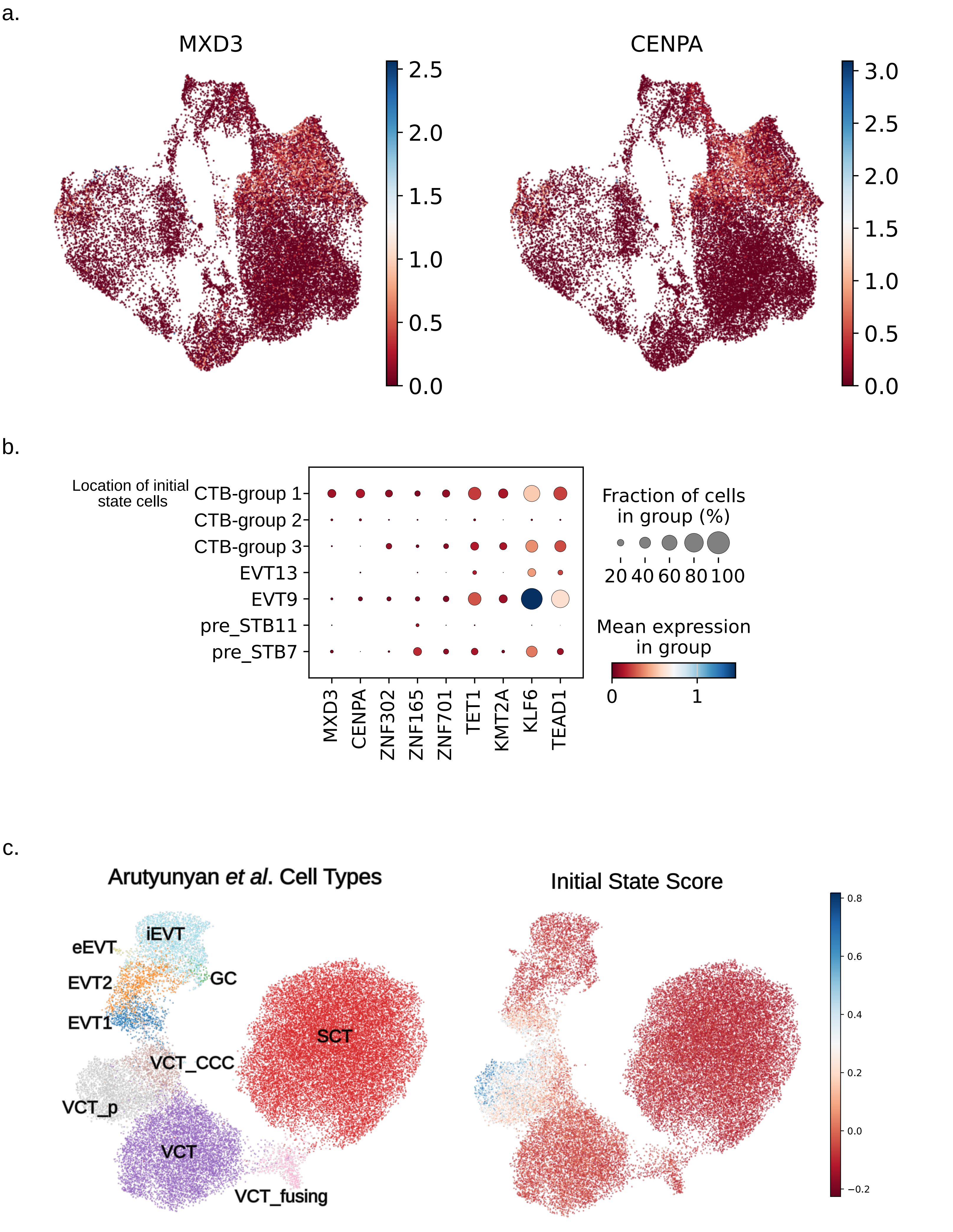
a.
MXD3
CENPA
0.0
0.5
1.0
1.5
2.0
2.5
3.0
b.
Location of initial state cells
CTB-group 1
CTB-group 2
CTB-group 3
EVT13
EVT9
pre_STB11
pre_STB7
MXD3
CENPA
ZNF302
ZNF165
ZNF701
TET1
KMT2A
KLF6
TEAD1
Fraction of cells in group (%)
20
40
60
80
100
Mean expression in group
0
1
c.
Arutyunyan et al. Cell Types
iEVT
eEVT
EVT2
GC
EVT1
SCT
VCT_CCC
VCT_p
VCT
VCT_fusing
Initial State Score
0.8
0.6
0.4
0.2
0.0
−0.2

Figure S4: Enriched transcriptional regulators in initial state cells. (a) UMAP showing the expression of the transcriptional regulators *MXD3* and *CENPA* that were differentially expressed (adj. p-value < 0.05 & log fold change > 1) in the initial state cells (n=30), compared to the rest of the trophoblast cells in our integrated first trimester trophoblast dataset (**Supplementary Data 10**), and were part of the CTB1 rank velocity genes (**Supplementary Data 9**). (b) Dot plot showing the mean cluster expression and the fraction of cells in each cluster expressing differentially expressed transcriptional regulators (adj. p-value < 0.05 & log fold change > 1) in the initial state cells (n=30) compared to the rest of the trophoblast cells in our integrated first trimester trophoblast dataset (**Supplementary Data 10**), and were part of the CTB1 rank velocity genes (**Supplementary Data 9**). (c) UMAP visualizations of snRNA-seq data from Arutyunyan *et al*[1] depicting the scores of the differentially-enriched (adj. p-value < 0.05 & log fold change > 2) initial state genes (n=130), found by performing differential expression between the cells highlighted in **Fig. 2b** and the rest of the cells in this UMAP, and listed in **Supplementary Data 10**.

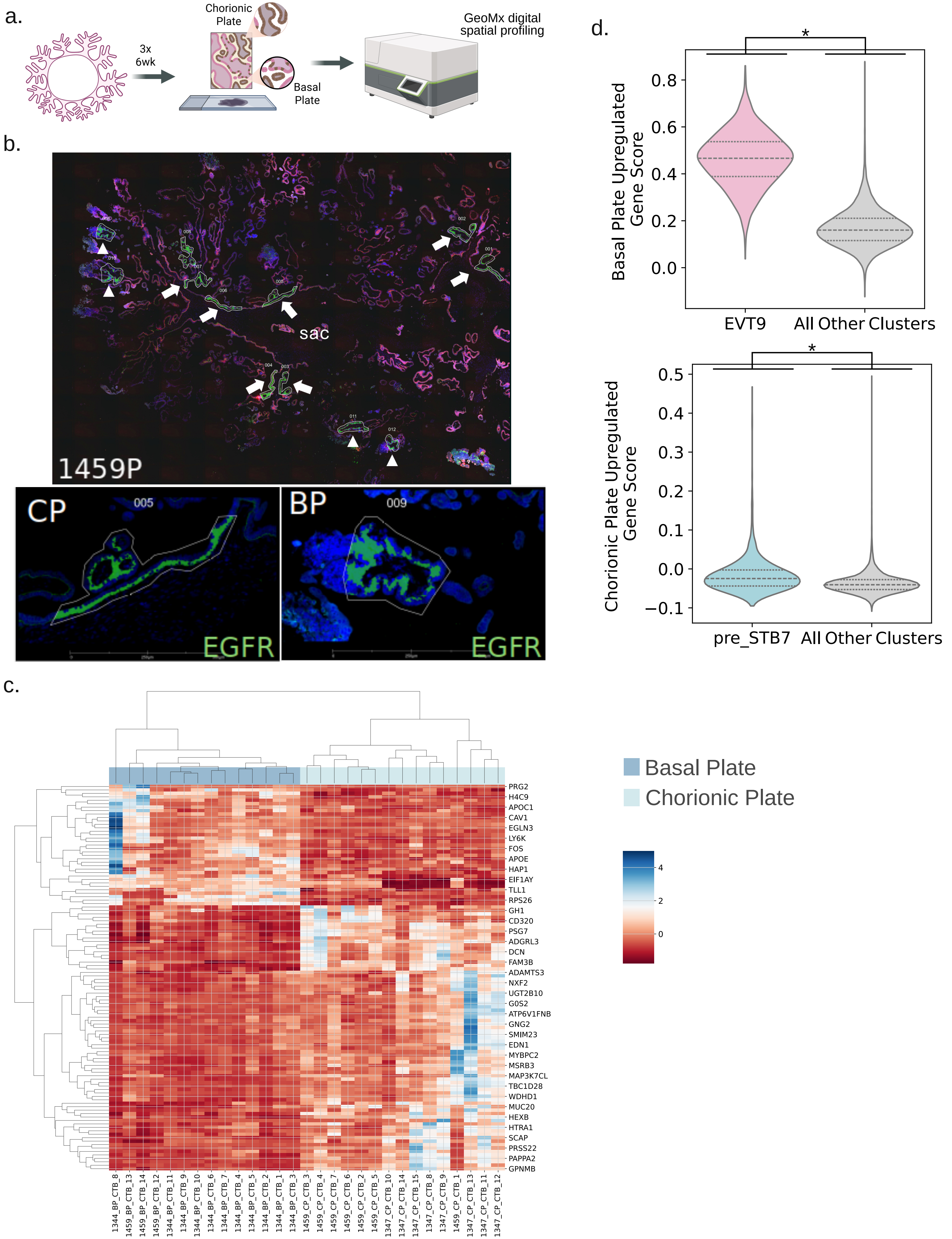

a.
Chorionic Plate
3x 6wk
Basal Plate
GeoMx digital spatial profiling
b.
sac
1459P
CP
005
EGFR
BP
009
EGFR
c.
Basal Plate
Chorionic Plate
PRG2
H4C9
APOC1
CAV1
EGLN3
LY6K
FOS
APOE
HAP1
EIF1AY
TLL1
RPS26
GH1
CD320
PSG7
ADGRL3
DCN
FAM3B
ADAMTS3
NXF2
UGT2B10
G0S2
ATP6V1FNB
GNG2
SMIM23
EDN1
MYBPC2
MSRB3
MAP3K7CL
TBC1D28
WDHD1
MUC20
HEXB
HTRA1
SCAP
PRSS22
PAPPA2
GPNMB
1344_BP_CTB_8
1459_BP_CTB_13
1459_BP_CTB_14
1459_BP_CTB_12
1344_BP_CTB_11
1344_BP_CTB_9
1344_BP_CTB_10
1344_BP_CTB_6
1344_BP_CTB_7
1344_BP_CTB_4
1344_BP_CTB_5
1344_BP_CTB_2
1344_BP_CTB_1
1344_BP_CTB_3
1459_CP_CTB_3
1459_CP_CTB_4
1459_CP_CTB_7
1459_CP_CTB_6
1459_CP_CTB_2
1459_CP_CTB_5
1347_CP_CTB_10
1347_CP_CTB_14
1347_CP_CTB_15
1347_CP_CTB_8
1347_CP_CTB_9
1459_CP_CTB_1
1347_CP_CTB_13
1347_CP_CTB_11
1347_CP_CTB_12
4
2
0
d.
*
Basal Plate Upregulated Gene Score
0.8
0.6
0.4
0.2
0.0
EVT9
All Other Clusters
*
Chorionic Plate Upregulated Gene Score
0.5
0.4
0.3
0.2
0.1
0.0
−0.1
pre_STB7
All Other Clusters

Figure S5: Spatial transcriptomic profiling of basal and chorionic plate CTB. (a) Schematic showing GeoMx-based digital spatial profiling of three early first trimester placentas, including both chorionic and basal plate regions. Created in BioRender. Soncin, F. (2026) https://BioRender.com/a1v76tu (b) Representative scan of formalin-fixed paraffin-embedded placental tissue (placenta # 1459P) used for GeoMx-based digital spatial profiling, showing tissue stained for EGFR to identify vCTB, with selection of Regions of Interest (ROI's) near the basal (BP, arrowheads) and chorionic plate (CP, arrows) based on spatial relation to the gestational sac, bottom. (c) Heatmap displaying the expression of genes that were determined to be differentially expressed (adj. P-value < 0.05 & log fold change > 1) between BP and CP CTB in 29 ROI's, located within three different placentas, using the GeoMx whole transcription atlas (WTA) panel. (d) Violin plots showing the score of the genes, found to be differentially enriched in the basal plate ROIs compared to the chorionic plate ROIs from the spatial transcriptomic dataset, in cluster EVT9 and all the other clusters (top), or the score of the genes, found to be differentially enriched in the chorionic plate ROIs compared to the basal plate ROIs from the spatial transcriptomic dataset, in cluster pre_STB7 and all the other clusters (bottom). Dashed lines indicate quartiles and * indicates Wilcoxon rank-sum p-value < 0.001.

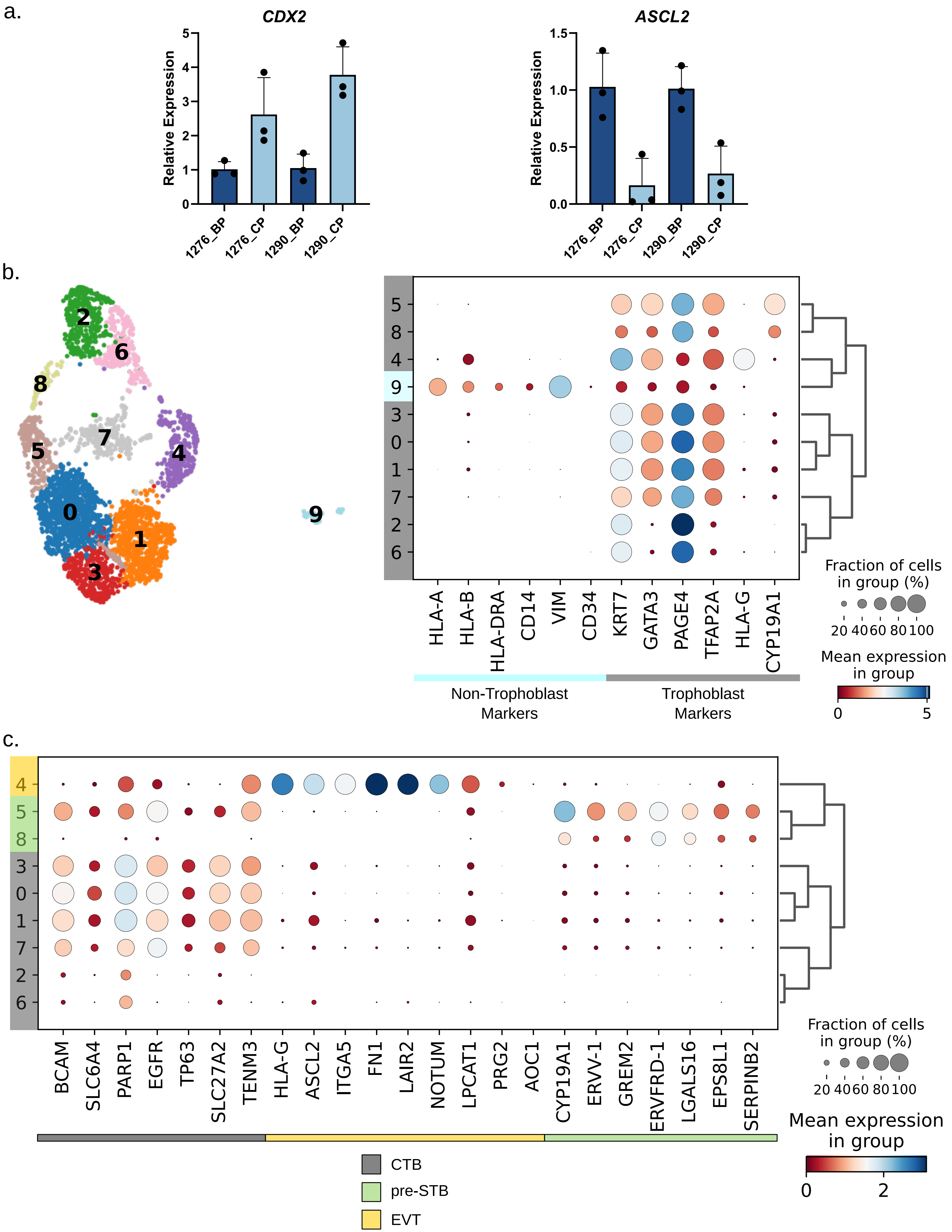

a.
CDX2
Relative Expression
1276_BP
1276_CP
1290_BP
1290_CP
ASCL2
Relative Expression
b.
HLA-A
HLA-B
HLA-DRA
CD14
VIM
CD34
KRT7
GATA3
PAGE4
TFAP2A
HLA-G
CYP19A1
Non-Trophoblast Markers
Trophoblast Markers
Fraction of cells in group (%)
Mean expression in group
c.
BCAM
SLC6A4
PARP1
EGFR
TP63
SLC27A2
TENM3
HLA-G
ASCL2
ITGA5
FN1
LAIR2
NOTUM
LPCAT1
PRG2
AOC1
CYP19A1
ERVV-1
GREM2
ERVFRD-1
LGALS16
EPS8L1
SERPINB2
Fraction of cells in group (%)
Mean expression in group
CTB
pre-STB
EVT

Figure S6: Single cell RNA-seq of basal and chorionic plate CTB. (a) Q-PCR showing the relative expression of the chorionic plate marker CDX2 and the basal plate marker ASCL2 in cells separated into basal plate (BP, dark blue) and chorionic plate (CP, light blue) fractions (n=3, data reported as mean ± standard deviation, measured in technical triplicate). Single-cell RNA-seq was then subsequently performed on these fractions. Data for bar charts can be found in (**Supplementary Data 19**). (b) UMAP showing the clustering of the basal and chorionic plate scRNA-seq dataset (left) and dot plot showing the mean expression and fraction of cells expressing various trophoblast (grey) and non-trophoblast (light blue) marker genes (right). (c) Dot plot and hierarchical clustering of cell clusters in the BP and CP dataset, showing the mean expression and fraction of cells expressing trophoblast cell type-specific markers (CTB in grey, EVT in yellow, and pre-STB in green).

a.

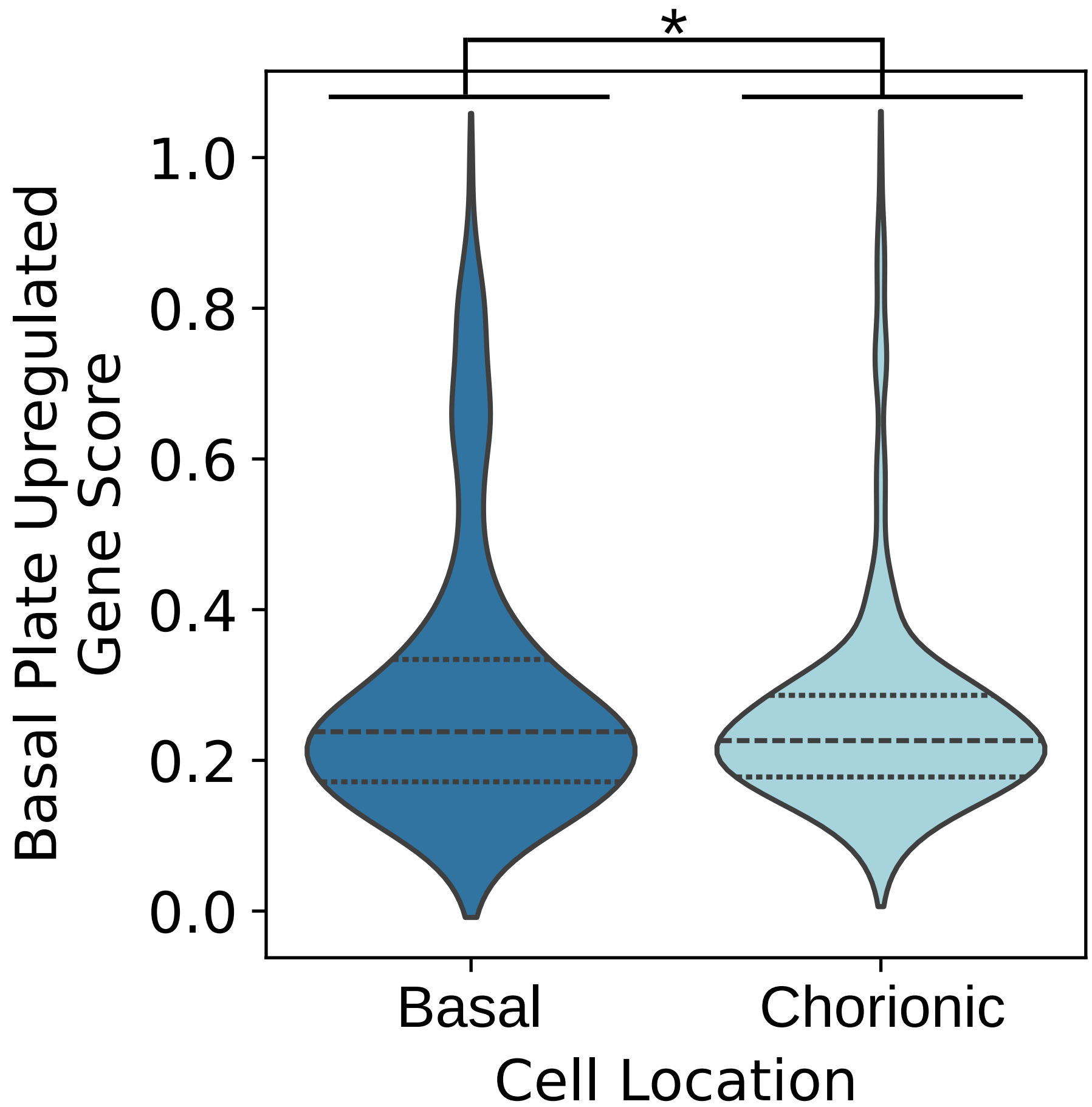

*
Basal Plate Upregulated Gene Score
1.0
0.8
0.6
0.4
0.2
0.0
Basal
Chorionic
Cell Location

b.

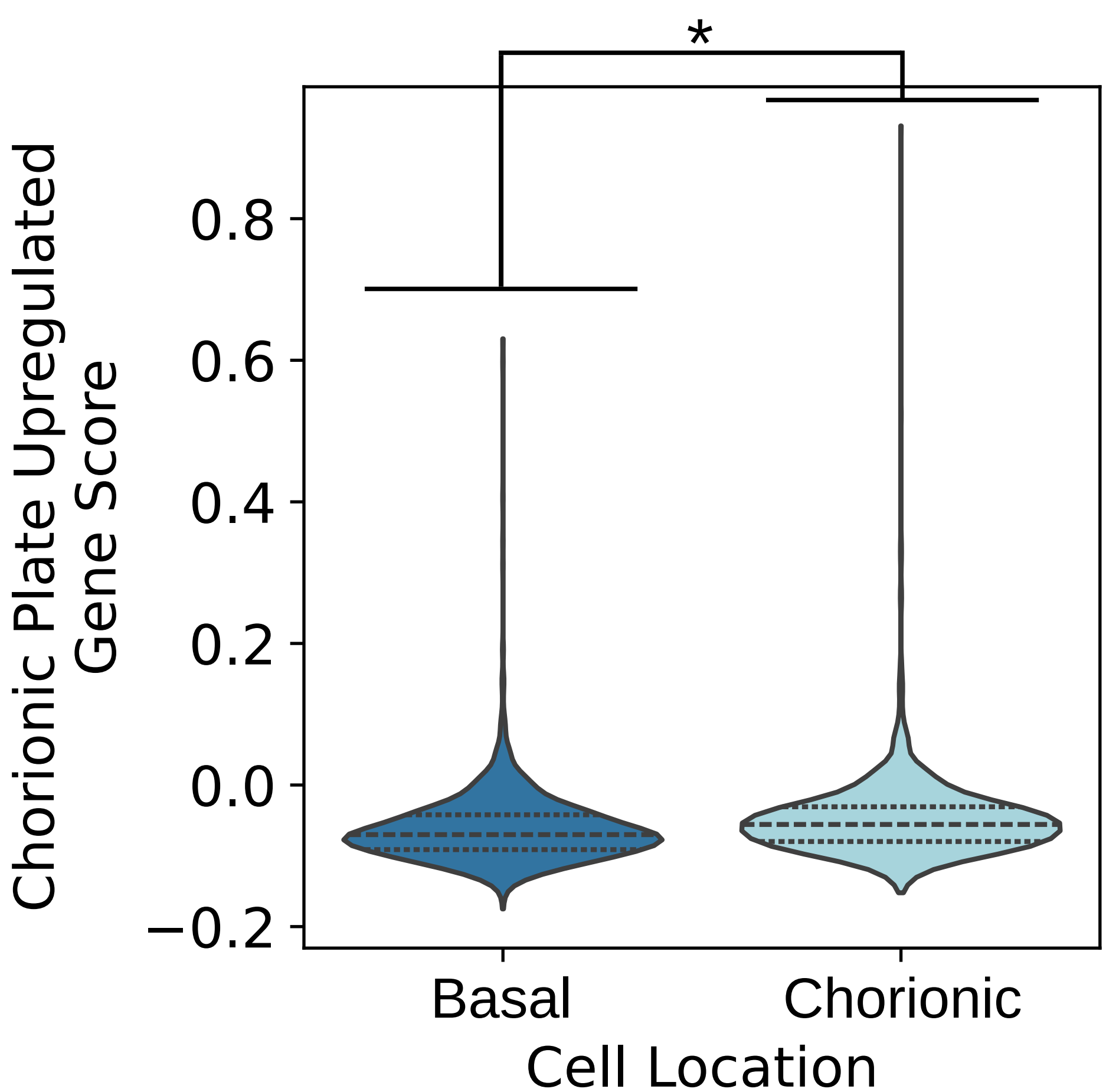

*
Chorionic Plate Upregulated Gene Score
0.8
0.6
0.4
0.2
0.0
−0.2
Basal
Chorionic
Cell Location

Figure S7: Basal and chorionic plate cell scoring using spatial transcriptomics data. (a) Violin plot showing the basal and chorionic plate cells, scored using the genes found to be differentially enriched in the basal plate regions of interest (ROIs) compared to the chorionic plate ROIs from the spatial transcriptomic dataset. (b) Violin plot showing the basal plate and chorionic plate cells scored using the genes found to be differentially enriched in the chorionic plate ROIs compared to the basal plate ROIs from the spatial transcriptomic dataset. Dashed lines indicate quartiles and * indicates Wilcoxon rank-sum p-value < 0.001.

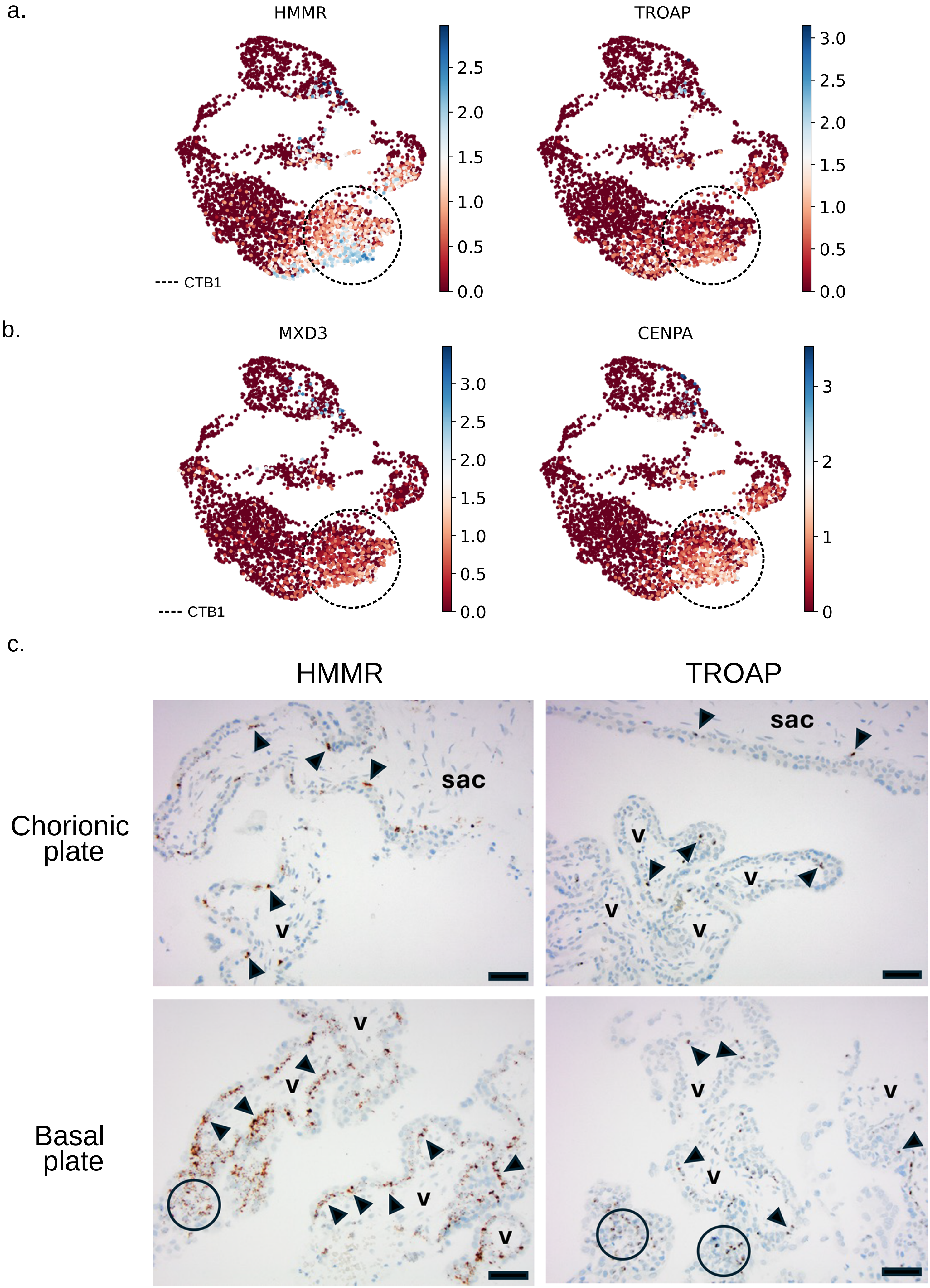
a.
HMMR
TROAP
CTB1
0.0
0.5
1.0
1.5
2.0
2.5
3.0
b.
MXD3
CENPA
CTB1
0
1
2
3
c.
HMMR
TROAP
Chorionic plate
Basal plate
sac
v

Figure S8: Expression of initial state marker genes in the basal and chorionic plate. (a) UMAPs showing the expression of initial state marker genes *HMMR* and *TROAP* in basal and chorionic plate scRNA-seq dataset. Cluster CTB1 is marked by a dotted circle. (b) UMAPs showing the expression of initial state transcription factors *MXD3* and *CENPA* in basal and chorionic plate scRNA-seq dataset. Cluster CTB1 is marked by a dotted circle. (c) *In-situ* hybridization of the initial state markers, *HMMR* and *TROAP*, in a 6-week placenta (same placenta as shown in **Fig. 2d**), comparing expression at/near basal vs. chorionic plate. Villous CTB in chorionic villi ("v") are marked by arrowheads. The gestational sac is labeled "sac," while the proximal column trophoblast are marked by a circle. Note higher signal (brown) at/near the basal plate, including in the trophoblast cell column. Scale bar=125 μm.

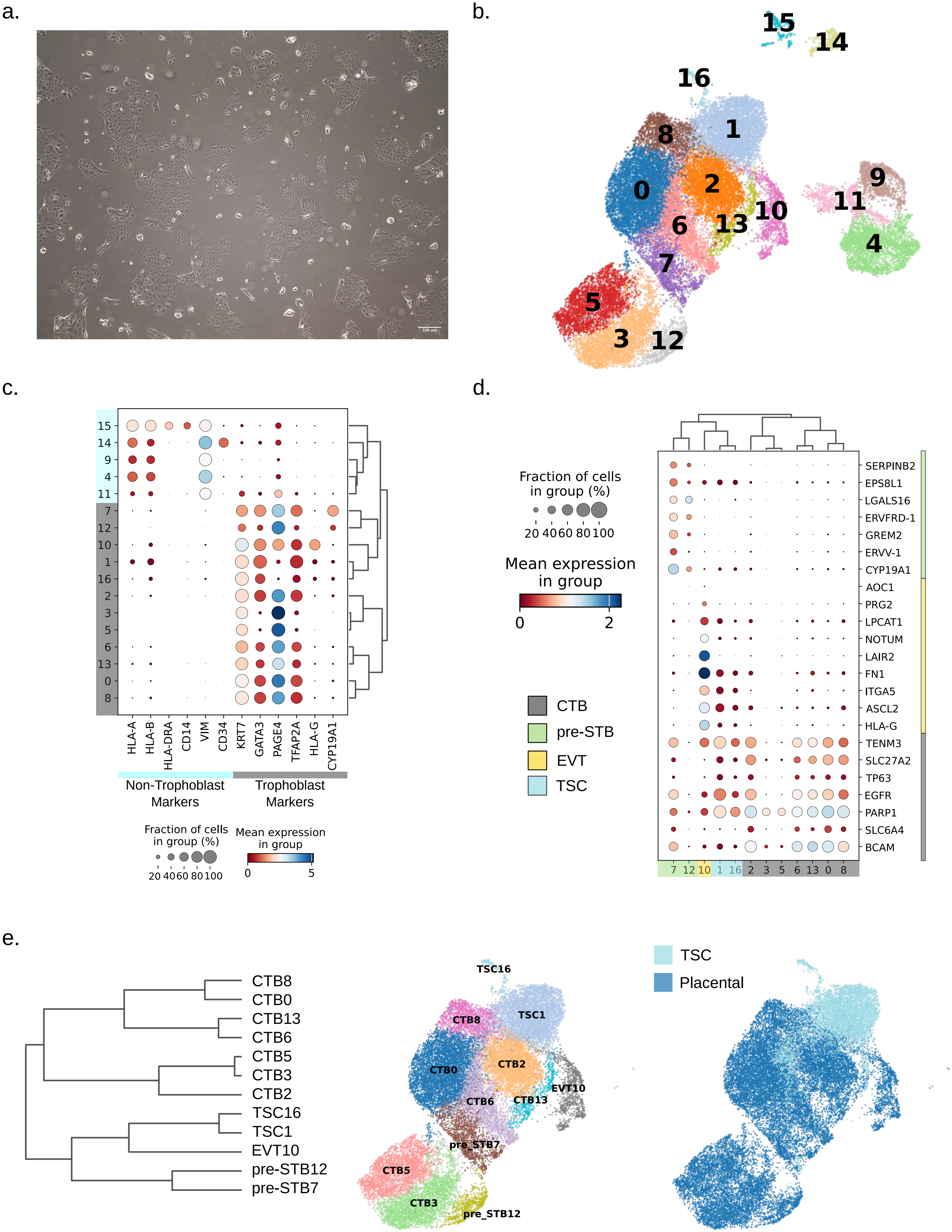
a.
b.
15
14
16
1
8
0
2
11
9
10
6
13
4
7
5
3
12
c.
15
14
9
4
11
7
12
10
1
16
2
3
5
6
13
0
8
HLA-A
HLA-B
HLA-DRA
CD14
VIM
CD34
KRT7
GATA3
PAGE4
TFAP2A
HLA-G
CYP19A1
Non-Trophoblast Markers
Trophoblast Markers
Fraction of cells in group (%)
20 40 60 80 100
Mean expression in group
0
5
d.
Fraction of cells in group (%)
20 40 60 80 100
Mean expression in group
0
2
CTB
pre-STB
EVT
TSC
SERPINB2
EPS8L1
LGALS16
ERVFRD-1
GREM2
ERVV-1
CYP19A1
AOC1
PRG2
LPCAT1
NOTUM
LAIR2
FN1
ITGA5
ASCL2
HLA-G
TENM3
SLC27A2
TP63
EGFR
PARP1
SLC6A4
BCAM
7 12 10 1 16 2 3 5 6 13 0 8
e.
CTB8
CTB0
CTB13
CTB6
CTB5
CTB3
CTB2
TSC16
TSC1
EVT10
pre-STB12
pre-STB7
TSC16
TSC1
CTB8
CTB0
CTB2
EVT10
CTB6
CTB13
pre_STB7
CTB5
CTB3
pre_STB12
TSC
Placental

Figure S9: Early first trimester and trophoblast stem cell (TSC) scRNA-seq dataset, following integration, clustering, and annotation. (a) Example bright-field image of trophoblast stem cells in 2D culture. Scale bar=100 μm. **(b)** UMAP of all cells from early first trimester placenta and TSC, following quality control filtering, integration, and clustering. (c) Dot plot and hierarchical clustering of cell clusters using trophoblast and non-trophoblast specific gene expression. (d) Dot plot and hierarchical clustering of cell clusters using cell type specific gene expression. (e) Hierarchical clustering dendrogram showing the transcriptomic similarity (Pearson correlation) between cell clusters (left), UMAP of clusters annotated based on cell type specific gene expression shown in part (c) (middle) and UMAP showing the location of TSC (light blue) vs. first trimester placental cells (dark blue) (right).

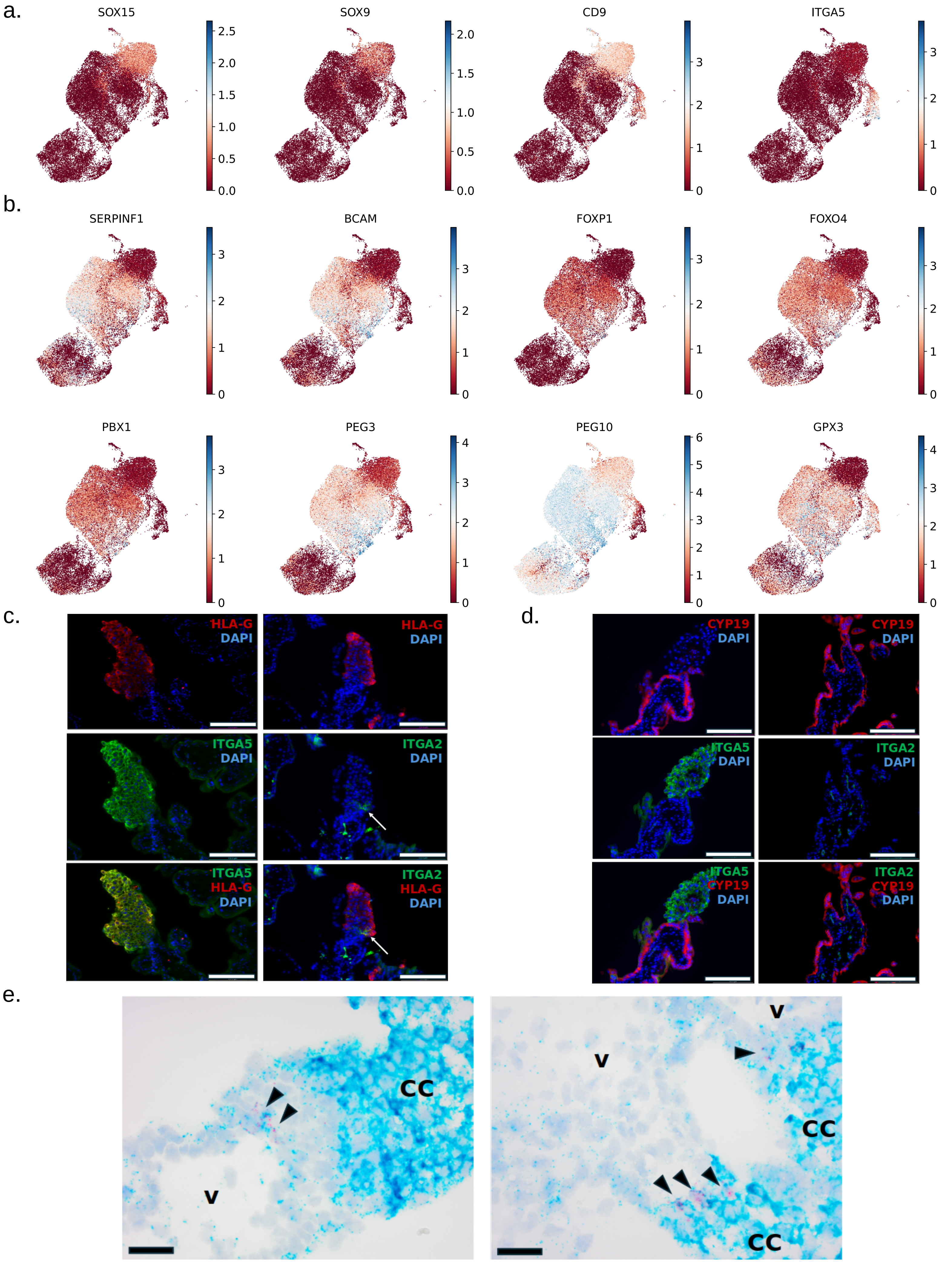

a.
SOX15
SOX9
CD9
ITGA5
b.
SERPINF1
BCAM
FOXP1
FOXO4
PBX1
PEG3
PEG10
GPX3
c.
HLA-G
DAPI
ITGA5
ITGA2
d.
CYP19
e.
v
cc

Figure S10: Differences in gene expression between TSC and initial state CTB. (a) UMAPs showing the expression of genes enriched in TSCs compared to initial state CTB. (b) UMAPs showing the expression of genes enriched in initial state CTB compared to TSCs. **(c-d)** Immunofluorescence of first trimester placenta with antibodies against ITGA5 and ITGA2, co-stained either with HLA-G (c) or CYP19A1 (d). ITGA5 is only expressed in column trophoblast, while ITGA2 is expressed in rare proximal column trophoblast (arrow in panel C), but also in fetal endothelial cells (panel D). Scale bar=156 μm. (e) *In-situ* hybridization of first trimester placenta with probes against *ITGA5* (teal) and *ITGA2* (magenta). Rare dual-positive cells are noted in the proximal columns (arrowheads). V=villous core; CC=cell column. Scale bar=62.5 μm.

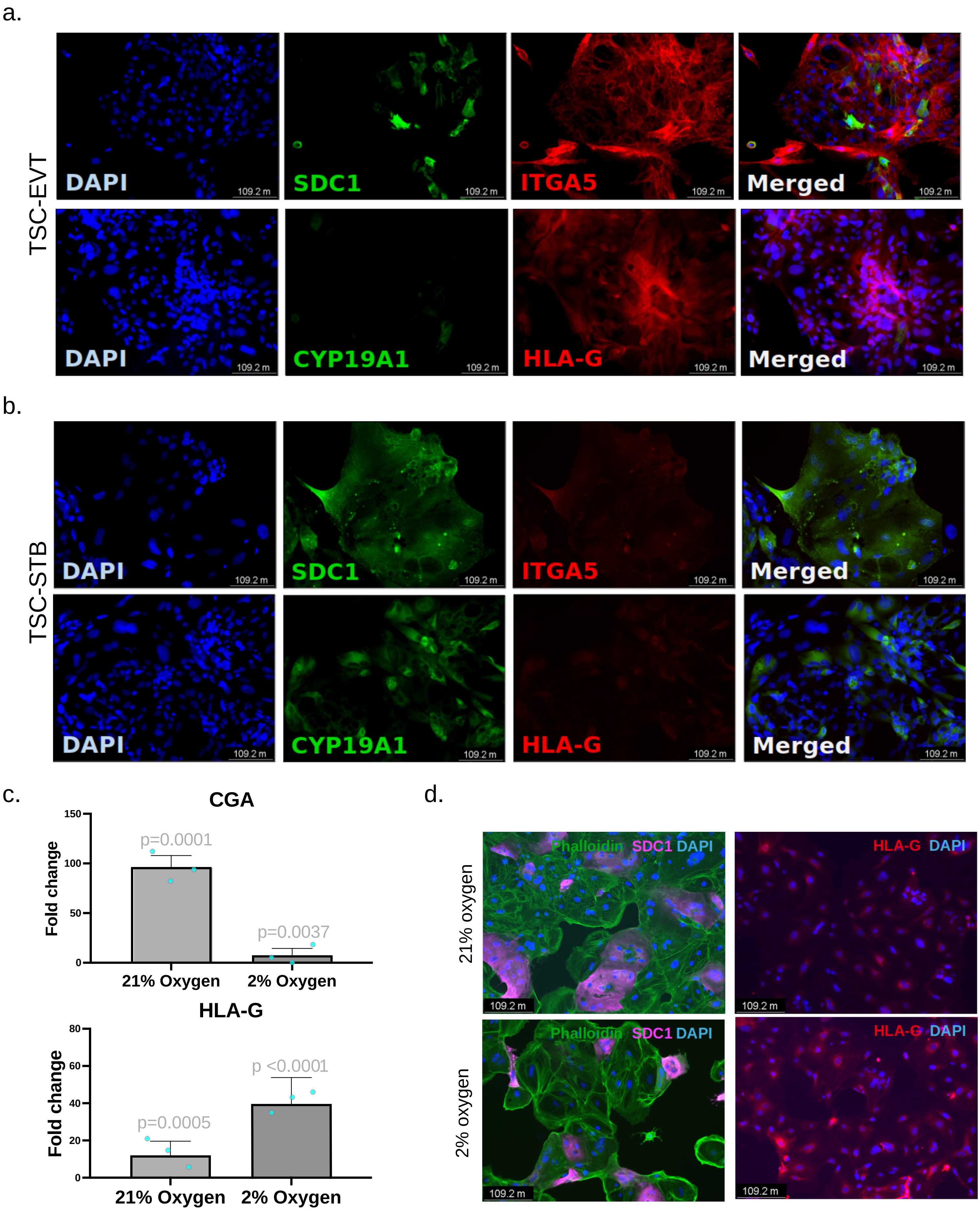

	CGA		HLA-G	
	21%	2%	21%	2%
Rep1 fold change	114.44	6.16	20.38	35.15
Rep2 fold change	85.22	3.54	14.34	42.95
Rep3 fold change	91.95	19.77	5.91	45.64

Figure S11: Directed and undirected differentiation of TSC. (a) Immunofluorescence of STB markers (SDC1 and CYP19A1), pcEVT marker (ITGA5), and EVT marker (HLA-G) following directed differentiation of TSCs to EVT. (b) Immunofluorescence of STB markers (SDC1 and CYP19A1), pcEVT marker (ITGA5), and EVT marker (HLA-G) following directed differentiation of TSC to STB. (c) Quantitative PCR of STB marker CGA, and EVT marker HLA-G, in TSC differentiated in an undirected manner over four days in 21% oxygen or 2% oxygen. Representative data on one TSC line measured in triplicate (n=3), presented as fold change over undifferentiated TSC, reported as mean ± standard deviation, with p-values calculated using one-way ANOVA. (d) Immunofluorescence of STB marker (SDC1) and EVT marker (HLA-G) following undirected differentiation of TSC (cultured in DMEM-F12+FBS in either 21% oxygen or 2% oxygen). By morphology and staining, the TSCs formed large SDC1^{+} multinucleated cells in 21% oxygen, but preferentially formed HLA-G^{+} mononuclear cells in 2% oxygen.

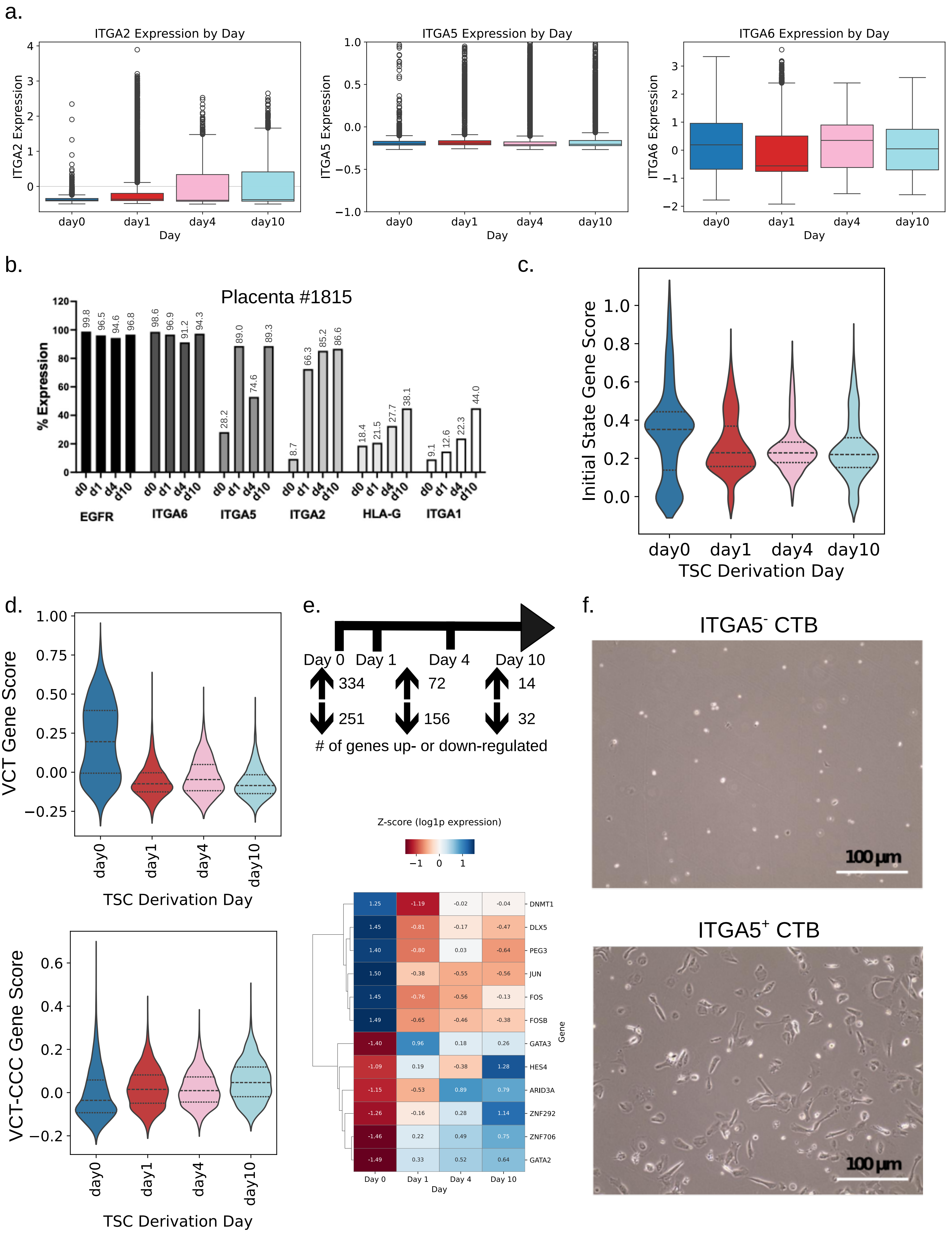
a.
ITGA2 Expression by Day
ITGA2 Expression
Day
day0
day1
day4
day10
ITGA5 Expression by Day
ITGA5 Expression
ITGA6 Expression by Day
ITGA6 Expression
b.
Placenta #1815
% Expression
99.8
96.5
94.6
96.8
98.6
96.9
91.2
94.3
28.2
89.0
74.6
89.3
8.7
66.3
85.2
86.6
18.4
21.5
27.7
38.1
9.1
12.6
22.3
44.0
d0 d1 d4 d10
EGFR
ITGA6
ITGA5
ITGA2
HLA-G
ITGA1
c.
Initial State Gene Score
TSC Derivation Day
d.
VCT Gene Score
TSC Derivation Day
VCT-CCC Gene Score
TSC Derivation Day
e.
Day 0
Day 1
Day 4
Day 10
334
251
72
156
14
32
# of genes up- or down-regulated
Z-score (log1p expression)
DNMT1
DLX5
PEG3
JUN
FOS
FOSB
GATA3
HES4
ARID3A
ZNF292
ZNF706
GATA2
Gene
Day
f.
ITGA5⁻ CTB
100 µm
ITGA5⁺ CTB
100 µm

Figure S12: Derivation of TSC from early first trimester CTB shows gradual progression toward a pcEVT phenotype. (a) Boxplots showing expression of *ITGA2*, *ITGA5*, and *ITGA6*, grouped by day based on scRNA-seq of CTB (isolated from placenta #1815), cultured in TSC derivation media. The center line indicates the median, box limits represent the interquartile range (IQR; 25th–75th percentile), whiskers extend to 1.5 × IQR, and points beyond the whiskers are plotted as outliers. (b) Percent of early gestation first trimester CTB expressing markers of CTB (EGFR, ITGA6), pcEVT (ITGA5, ITGA2), pan-EVT (HLA-G) and mature EVT (ITGA1), by flow cytometry, at 4 timepoints during TSC derivation, in placenta #1815. (c) Violin plot showing initial state scoring of cells grouped by day of derivation, in placenta #1815. Scoring was done using the significantly enriched genes in initial state cells in first trimester placenta (**Fig. 2b, Supplementary Data 10**). Dashed lines represent quartiles. (d) Violin plots grouped by day of derivation, scoring cells from placenta #1815, based on the top 50 uniquely expressed genes as outlined by Arutyunyan *et al*[1], for the cell types villous cytotrophoblast (VCT) and cytotrophoblast cell columns derived from VCT (VCT-CCC). Dashed lines represent quartiles. (e) Diagram (top) showing the number of genes differentially expressed between sequential timepoints (adj. p-value < 0.05 and log fold change > |1|) (**Supplementary Data 15**). Heatmap (below) shows the z-score (log1p expression) of 12 transcriptional regulators that are either negatively or positively correlated during derivation of TSC from placenta #1815 (**Supplementary Data 17**). (f) Isolated CTB and MACS-sorted for ITGA5 cells plated in 2D. Pictures show that only $ITGA5^+$ cells adhered to the tested substrates (fibronectin), in both oxygen tensions tested (21% or 2%, 21% shown), in TSC media, while very few $ITGA5^-$ cells did the same.

Supplementary References

1) Arutyunyan, A. et al. Spatial multiomics map of trophoblast development in early pregnancy. Nature 616, 143–151 (2023).
2) Vento-Tormo, R., Efremova, M., Botting, R.A. et al. Single-cell reconstruction of the early maternal–fetal interface in humans. Nature 563, 347–353 (2018).
3) Shannon, M. J. et al. Cell trajectory modeling identifies a primitive trophoblast state defined by bcam enrichment. Development 149 (2022).
