# Supplementary material for: Single-cell transcriptomics reveals differences between chorionic and basal plate cytotrophoblasts and 2D-cultured trophoblast stem cells
Source: Commun Biol. 2026 Jun 10;9:1234. doi: 10.1038/s42003-026-10460-0 (PMC13594312; doi:10.1038/s42003-026-10460-0)
Supplement: Supplementary file 3 [file 42003_2026_10460_MOESM3_ESM.pdf]

**Description of Additional Supplementary File**

File name: Supplementary data 1
Description: Sample and sequencing metrics.

File name: Supplementary data 2
Description: Top 200 marker genes in each cluster in the first trimester placenta samples.

File name: Supplementary data 3
Description: Marker genes after removal of non-trophoblast clusters, reclustering, and annotation of clusters.

File name: Supplementary data 4
Description: Cell numbers of each cluster by gestational age for UMAP shown in Fig. S2d.

File name: Supplementary data 5
Description: Top 200 marker genes in each cluster group of the merged primary placenta dataset shown in Fig. 1e.

File name: Supplementary data 6
Description: Genes highly expressed (adj. p-value < 0.05 and log fold change > 1) in CTB3 (primarily composed of late first trimester CTB) vs CTB1 and CTB2 (primarily composed of early first trimester CTB) (Top) and genes highly expressed (> 1 Log2 fold change and adj. p-value < 0.05) in CTB1 and CTB2 (primarily composed of early first trimester CTB) vs CTB3 (primarily composed of late first trimester CTB) (Bottom). Log fold changes of early (CTB4 cluster) vs. late (CTB6 cluster) first trimester CTB from a similar comparison using data from (Shannon et al.)25 are shown in the right-most columns in each table.

File name: Supplementary data 7
Description: Genes highly expressed (> 1.5 Log2 fold change and adj. p-value < 0.05) in CTB1 vs CTB2 (both primarily composed of early first trimester CTB).

File name: Supplementary data 8
Description: Genes highly expressed (> 1.5 Log2 fold change and adj. p-value < 0.05) in CTB2 vs CTB3 (both primarily composed of early first trimester CTB).

File name: Supplementary data 9
Description: Genes that best explain the RNA velocity vector field in Fig. 2a. These genes, called rank velocity genes, may play a role in driving the differentiation of CTB. Rank velocity genes with a minimum r2 value of 0.5 and a minimum correlation coefficient between spliced and unspliced genes of 0.3 by cluster group.

File name: Supplementary data 10
Description: Genes highly expressed (adj. p-value < 0.05 and log fold change > 1) in initial state cells (n=30) vs all other cells in the early and late first trimester integrated placental dataset.

File name: Supplementary data 11
Description: Genes highly expressed (adj. p-value < 0.05 and log fold change > 1) in either the basal plate ROIs or chorionic plate ROIs in GeoMx spatial WTA dataset.

File name: Supplementary data 12
Description: Top 200 marker genes of all clusters after integration of early first trimester trophoblast and TSC single cell data (Fig. 4b).

File name: Supplementary data 13
Description: Correlation values and p-values from permutation test (n=1000) along with UMAP distance statistics between each of Okae et al’s (2018)7 bulk TSC RNA-seq samples and the early first trimester and TSC integrated scRNA-seq dataset.

File name: Supplementary data 14
Description: Top 200 genes enriched (adj. p-value < 0.05 and log fold change > 1) in initial state cells (n=30) vs TSC, in the early first trimester and TSC integrated dataset (Fig. 4b).

File name: Supplementary data 15
Description: Differential expression between sequential timepoints during TSC derivation (adj. p-value < 0.05 and log fold change > |1|).

File name: Supplementary data 16
Description: Genes significantly correlated with TSC derivation pseudotime (r > |0.3|).

File name: Supplementary data 17
Description: Transcriptional regulators significantly correlated with TSC derivation pseudotime (r > |0.2|)

File name: Supplementary data 18
Description: KEGG pathways enriched during sequential timepoints in TSC derivation (adj. p-value < 0.05)

File name: Supplementary data 19
Description: qPCR relative expression values to create the bar charts in Fig. S6a.

File name: Supplementary data 20
Description: Antibody information.
