# Supplementary material for: Single-cell transcriptomics reveals differences between chorionic and basal plate cytotrophoblasts and 2D-cultured trophoblast stem cells
Source: Commun Biol. 2026 Jun 10;9:1234. doi: 10.1038/s42003-026-10460-0 (PMC13594312; doi:10.1038/s42003-026-10460-0)
Supplement: Supplementary file 4 [file 42003_2026_10460_MOESM5_ESM.pdf]

nature portfolio

Corresponding author(s): Mana Parast

Last updated by author(s): Robert Morey - May 14, 2026

Reporting Summary

Nature Portfolio wishes to improve the reproducibility of the work that we publish. This form provides structure for consistency and transparency in reporting. For further information on Nature Portfolio policies, see our Editorial Policies and the Editorial Policy Checklist.

Statistics

For all statistical analyses, confirm that the following items are present in the figure legend, table legend, main text, or Methods section.

n/a	Confirmed	
☐	☒	The exact sample size (*n*) for each experimental group/condition, given as a discrete number and unit of measurement
☐	☒	A statement on whether measurements were taken from distinct samples or whether the same sample was measured repeatedly
☐	☒	The statistical test(s) used AND whether they are one- or two-sided<br>*Only common tests should be described solely by name; describe more complex techniques in the Methods section.*
☐	☒	A description of all covariates tested
☐	☒	A description of any assumptions or corrections, such as tests of normality and adjustment for multiple comparisons
☐	☒	A full description of the statistical parameters including central tendency (e.g. means) or other basic estimates (e.g. regression coefficient) AND variation (e.g. standard deviation) or associated estimates of uncertainty (e.g. confidence intervals)
☐	☒	For null hypothesis testing, the test statistic (e.g. *F*, *t*, *r*) with confidence intervals, effect sizes, degrees of freedom and *P* value noted<br>*Give P values as exact values whenever suitable.*
☐	☒	For Bayesian analysis, information on the choice of priors and Markov chain Monte Carlo settings
☐	☒	For hierarchical and complex designs, identification of the appropriate level for tests and full reporting of outcomes
☐	☒	Estimates of effect sizes (e.g. Cohen's *d*, Pearson's *r*), indicating how they were calculated

Our web collection on statistics for biologists contains articles on many of the points above.

Software and code

Policy information about availability of computer code

Data collection	Illumina NovaSeq 6000, 10x Genomics Chromium Controller, GeoMx digital spatial profiler (Nanostring), FortessaX20 or Fortessa (BDLSRFortessa) flow cytometer (BD Biosciences), Olympus BX43 microscope (Olympus), et al.
Data analysis	STAR (v.2.7.10a), CellBender's (v. 0.3.0), Scanpy (v. 1.10.1),scvi-tools (v. 1.1.2), scVelo (v. 0.3.2), GSEApy (v1.1.11), CellRank2 (v. 2.0.4), FlowJo, GraphPad Prism, GeoMx NGS pipeline (v.2.0.0.16), GeoMx DSP software, et al.

For manuscripts utilizing custom algorithms or software that are central to the research but not yet described in published literature, software must be made available to editors and reviewers. We strongly encourage code deposition in a community repository (e.g. GitHub). See the Nature Portfolio guidelines for submitting code & software for further information.

Data

Policy information about availability of data

All manuscripts must include a data availability statement. This statement should provide the following information, where applicable:

- Accession codes, unique identifiers, or web links for publicly available datasets
- A description of any restrictions on data availability
- For clinical datasets or third party data, please ensure that the statement adheres to our policy

Single-cell RNA-seq data and GeoMx digital spatial profiler data have been deposited at GEO and are publicly available as of the date of publication. Accession

numbers are under GSE270174. Microscopy data reported in this paper will be shared by the lead contact upon request. Code used for data processing and analyses are available https://github.com/remorey/Placental_Single_Cell_Analyses as of date of publication.

Research involving human participants, their data, or biological material

Policy information about studies with human participants or human data. See also policy information about sex, gender (identity/presentation), and sexual orientation and race, ethnicity and racism.

Reporting on sex and gender	Sex of each sample is listed in Table S1 but the biological sex of the placental tissues used in this manuscript was not considered in the analyses presented here.
Reporting on race, ethnicity, or other socially relevant groupings	Not available
Population characteristics	All samples were collected from pregnant individuals, undergoing elective termination of pregnancy, providing written informed consent. Gestational age was determined based on crown-rump length, measured on ultrasound, and was stated in weeks/days from the first day of the last menstrual period. “Normal” sample was defined as a singleton pregnancy without any detectable fetal abnormalities on ultrasound.
Recruitment	All samples were collected from pregnant individuals, undergoing elective termination of pregnancy, providing written informed consent.
Ethics oversight	Human placental tissue samples for this study were collected under a UC San Diego Institutional Review Board-approved protocol; all patients gave informed consent for collection and use of these tissues.

Note that full information on the approval of the study protocol must also be provided in the manuscript.

Field-specific reporting

Please select the one below that is the best fit for your research. If you are not sure, read the appropriate sections before making your selection.

☒ Life sciences ☐ Behavioural & social sciences ☐ Ecological, evolutionary & environmental sciences

For a reference copy of the document with all sections, see nature.com/documents/nr-reporting-summary-flat.pdf

Life sciences study design

All studies must disclose on these points even when the disclosure is negative.

Sample size	Table S1 lists the sample sizes for each of the analyses. Our early first trimester single cell primary placental data consisted of samples from four patients and our late first trimester primary samples came from two patients. Two of the early patients contributed both basal plate and chorionic plate samples. TSC single cell samples were derived from six different patients and our GeoMx spatial profiling was performed on three different patient samples. Flow cytometry was performed on early first trimester placenta from three patients.
Data exclusions	Data not meeting standards spelled out in the methods were filtered out before performing analyses.
Replication	Replicates were used for every experiment and are stated in the manuscript.
Randomization	Samples were selected and grouped based gestational age and availability.
Blinding	Blinding was not relevant to our study because we were interested in differences between early and late first trimester placenta, basal and chorionic plate placenta, and differences between 2D TSC and in vivo placenta samples.

Behavioural & social sciences study design

All studies must disclose on these points even when the disclosure is negative.

Study description	N/A
Research sample	N/A
Sampling strategy	N/A
Data collection	N/A
Timing	N/A

Data exclusions	N/A
Non-participation	N/A
Randomization	N/A

Ecological, evolutionary & environmental sciences study design

All studies must disclose on these points even when the disclosure is negative.

Study description	N/A
Research sample	N/A
Sampling strategy	N/A
Data collection	N/A
Timing and spatial scale	N/A
Data exclusions	N/A
Reproducibility	N/A
Randomization	N/A
Blinding	N/A

Did the study involve field work? ☐ Yes ☒ No

Reporting for specific materials, systems and methods

We require information from authors about some types of materials, experimental systems and methods used in many studies. Here, indicate whether each material, system or method listed is relevant to your study. If you are not sure if a list item applies to your research, read the appropriate section before selecting a response.

Materials & experimental systems

n/a	Involved in the study
☐	☒ Antibodies
☐	☒ Eukaryotic cell lines
☒	☐ Palaeontology and archaeology
☒	☐ Animals and other organisms
☒	☐ Clinical data
☒	☐ Dual use research of concern
☒	☐ Plants

Methods

n/a	Involved in the study
☒	☐ ChIP-seq
☐	☒ Flow cytometry
☒	☐ MRI-based neuroimaging

Antibodies

Antibodies used	PE-conjugated anti-ITGA6 antibody (Biolegend #313612), APC-conjugated anti-EGFR antibody (Biolegend #352906), ITGA5-FITC antibody (Biolegend #328008), EGFR-APC (Biolegend #352906), HLAG-PE (ExBIO #1P-292-C100), ITGA6-AF647 (Biolegend #313610), ITGA5-FITC (Biolegend #328008), ITGA2-AF594 (R&D #FAB1233T), EGFR-PE/Cy7 (Biolegend #352910), HLAG-APC (ExBIO #1A-292-C100), ITGA1-PE (Biolegend #328304), ITGA2-AF700 (R&D #FAB1233N), ITGA5-FITC (Biolegend #328008), ITGA6-BV421 (Biolegend #313624), mouse anti-SDC1 (Abcam # ab34164), rabbit anti-ITGA5 (Abcam, #ab150361), mouse anti-CYP19A1 (Novus Biologicals, #NBP3-07826), Rabbit anti-HLAG (Abcam #ab283260), mouse anti-HLAG (Abcam, #ab283270), rabbit anti-ITGA2 (Bioss, #bsm-52613R), rabbit anti-ITGA5 (Abcam, #ab150361), CONFIRM® anti-EGFR (5B7) Rabbit Monoclonal Primary Antibody (Ventana #790-4347), anti-HLA-G (4H48) mouse primary antibody (Abcam #ab52455), Alexa Fluor anti-rabbit-647 (Life Technologies), Alexa Fluor anti-mouse-594 (Life Technologies), and SYTO13 (Nanostring). See supplementary data table for more antibody information.
Validation	See validation on websites of companies listed alongside above antibodies.

Eukaryotic cell lines

Policy information about cell lines and Sex and Gender in Research

Cell line source(s)	Trophoblast stem cell lines were isolated from first trimester (GA 6-8 weeks; n=8) CTB were MACS-purified with either a PE-conjugated anti-ITGA6 antibody (Biolegend #313612) or an APC-conjugated anti-EGFR antibody (Biolegend #352906), except for the ten day TSC derivation experiment for which no MACS-purification was used. MACS-purified CTB were then plated for TSC derivation as previously described and cultured with modified TSC media.
Authentication	Cell lines were authenticated by testing for expression of various surface markers, including EGFR, HLAG, and a panel of integrins by flow cytometry.
Mycoplasma contamination	All cell lines tested negative for mycoplasma contamination
Commonly misidentified lines (See ICLAC register)	N/A

Palaeontology and Archaeology

Specimen provenance	N/A
Specimen deposition	N/A
Dating methods	N/A

☐ Tick this box to confirm that the raw and calibrated dates are available in the paper or in Supplementary Information.

Ethics oversight	N/A

Note that full information on the approval of the study protocol must also be provided in the manuscript.

Animals and other research organisms

Policy information about studies involving animals; ARRIVE guidelines recommended for reporting animal research, and Sex and Gender in Research

Laboratory animals	N/A
Wild animals	N/A
Reporting on sex	N/A
Field-collected samples	N/A
Ethics oversight	N/A

Note that full information on the approval of the study protocol must also be provided in the manuscript.

Clinical data

Policy information about clinical studies

All manuscripts should comply with the ICMJE guidelines for publication of clinical research and a completed CONSORT checklist must be included with all submissions.

Clinical trial registration	N/A
Study protocol	N/A
Data collection	N/A
Outcomes	N/A

Dual use research of concern

Policy information about dual use research of concern

Hazards

Could the accidental, deliberate or reckless misuse of agents or technologies generated in the work, or the application of information presented in the manuscript, pose a threat to:

No	Yes	
☒	☐	Public health
☒	☐	National security
☒	☐	Crops and/or livestock
☒	☐	Ecosystems
☒	☐	Any other significant area

Experiments of concern

Does the work involve any of these experiments of concern:

No	Yes	
☒	☐	Demonstrate how to render a vaccine ineffective
☒	☐	Confer resistance to therapeutically useful antibiotics or antiviral agents
☒	☐	Enhance the virulence of a pathogen or render a nonpathogen virulent
☒	☐	Increase transmissibility of a pathogen
☒	☐	Alter the host range of a pathogen
☒	☐	Enable evasion of diagnostic/detection modalities
☒	☐	Enable the weaponization of a biological agent or toxin
☒	☐	Any other potentially harmful combination of experiments and agents

Plants

Seed stocks	N/A
Novel plant genotypes	N/A
Authentication	N/A

ChIP-seq

Data deposition

☐ Confirm that both raw and final processed data have been deposited in a public database such as GEO.

☐ Confirm that you have deposited or provided access to graph files (e.g. BED files) for the called peaks.

Data access links<br>*May remain private before publication.*	N/A
Files in database submission	N/A
Genome browser session<br>(e.g. UCSC)	N/A

Methodology

Replicates	N/A
Sequencing depth	N/A
Antibodies	N/A
Peak calling parameters	N/A

Data quality: N/A

Software: N/A

Flow Cytometry

Plots

Confirm that:

☒ The axis labels state the marker and fluorochrome used (e.g. CD4-FITC).

☒ The axis scales are clearly visible. Include numbers along axes only for bottom left plot of group (a 'group' is an analysis of identical markers).

☒ All plots are contour plots with outliers or pseudocolor plots.

☒ A numerical value for number of cells or percentage (with statistics) is provided.

Methodology

Sample preparation	Isolated CTB and derived TSCs were stained, either individually with EGFR-APC (Biolegend #352906), HLAG-PE (ExBIO #1P-292-C100), ITGA6-AF647 (Biolegend #313610), ITGA5-FITC (Biolegend #328008) and ITGA2-AF594 (R&D #FAB1233T), or using a multi-color panel of EGFR-PE/Cy7 (Biolegend #352910), HLAG-APC (ExBIO #1A-292-C100), ITGA1-PE (Biolegend #328304), ITGA2-AF700 (R&D #FAB1233N), ITGA5-FITC (Biolegend #328008) and ITGA6-BV421 (Biolegend #313624), with relative isotype control antibodies, for 1 hour in flow cytometer wash buffer (1% FBS, 2%BSA and 0.03% Sodium Azide in PBS) in the dark. After staining, cells were washed three times with wash buffer and fixed with 2% PFA.
Instrument	FortessaX20 or Fortessa (BDLSRFortessa) flow cytometer (BD Biosciences)
Software	FlowJo
Cell population abundance	Not applicable
Gating strategy	See data deposited in https://github.com/remorey/Placental_Single_Cell_Analyses

☒ Tick this box to confirm that a figure exemplifying the gating strategy is provided in the Supplementary Information.

Magnetic resonance imaging

Experimental design

Design type	N/A
Design specifications	N/A
Behavioral performance measures	N/A

Acquisition

Imaging type(s)	N/A
Field strength	N/A
Sequence & imaging parameters	N/A
Area of acquisition	N/A

Diffusion MRI ☐ Used ☒ Not used

Preprocessing

Preprocessing software	N/A
Normalization	N/A
Normalization template	N/A
Noise and artifact removal	N/A

Volume censoring | N/A

Statistical modeling & inference

Model type and settings | N/A

Effect(s) tested | N/A

Specify type of analysis: ☐ Whole brain ☐ ROI-based ☐ Both

Statistic type for inference | N/A

(See Eklund et al. 2016)

Correction | N/A

Models & analysis

n/a	Involved in the study
☒	☐ Functional and/or effective connectivity
☒	☐ Graph analysis
☒	☐ Multivariate modeling or predictive analysis
